# Estimation of tumor cell total mRNA expression in 15 cancer types predicts disease progression

**DOI:** 10.1038/s41587-022-01342-x

**Published:** 2022-06-13

**Authors:** Shaolong Cao, Jennifer R. Wang, Shuangxi Ji, Peng Yang, Yaoyi Dai, Shuai Guo, Matthew D. Montierth, John Paul Shen, Xiao Zhao, Jingxiao Chen, Jaewon James Lee, Paola A. Guerrero, Nicholas Spetsieris, Nikolai Engedal, Sinja Taavitsainen, Kaixian Yu, Julie Livingstone, Vinayak Bhandari, Shawna M. Hubert, Najat C. Daw, P. Andrew Futreal, Eleni Efstathiou, Bora Lim, Andrea Viale, Jianjun Zhang, Matti Nykter, Bogdan A. Czerniak, Powel H. Brown, Charles Swanton, Pavlos Msaouel, Anirban Maitra, Scott Kopetz, Peter Campbell, Terence P. Speed, Paul C. Boutros, Hongtu Zhu, Alfonso Urbanucci, Jonas Demeulemeester, Peter Van Loo, Wenyi Wang

**Affiliations:** 1 Department of Bioinformatics and Computational Biology, The University of Texas MD Anderson Cancer Center, Houston, TX USA; 2 Department of Head and Neck Surgery, The University of Texas MD Anderson Cancer Center, Houston, TX USA; 3 Department of Statistics, Rice University, Houston, TX USA; 4 Baylor College of Medicine, Houston, TX USA; 5 Department of Gastrointestinal Medical Oncology, The University of Texas MD Anderson Cancer Center, Houston, TX USA; 6 Sheikh Ahmed Center for Pancreatic Cancer Research, The University of Texas MD Anderson Cancer Center, Houston, TX USA; 7 Department of Translational Molecular Pathology, The University of Texas MD Anderson Cancer Center, Houston, TX USA; 8 Department of Surgical Oncology, The University of Texas MD Anderson Cancer Center, Houston, TX USA; 9 Department of Genitourinary Medical Oncology, The University of Texas MD Anderson Cancer Center, Houston, TX USA; 10 Department of Tumor Biology, Institute for Cancer Research, Oslo University Hospital, Oslo, Norway; 11 Faculty of Medicine and Health Technology, Tampere University and Tays Cancer Center, Tampere, Finland; 12 Department of Biostatistics, The University of Texas MD Anderson Cancer Center, Houston, TX USA; 13 Department of Human Genetics, University of California, Los Angeles, Los Angeles, CA USA; 14 Department of Urology, University of California, Los Angeles, Los Angeles, CA USA; 15 Institute for Precision Health, University of California, Los Angeles, Los Angeles, CA USA; 16 Jonsson Comprehensive Cancer Center, University of California, Los Angeles, Los Angeles, CA USA; 17 Department of Medical Biophysics, University of Toronto, Toronto ON, Canada; 18 Department of Thoracic Head Neck Medical Oncology, The University of Texas MD Anderson Cancer Center, Houston, TX USA; 19 Department of Pediatrics, The University of Texas MD Anderson Cancer Center, Houston, TX USA; 20 Department of Genomic Medicine, The University of Texas MD Anderson Cancer Center, Houston, TX USA; 21 Department of Breast Medical Oncology, The University of Texas MD Anderson Cancer Center, Houston, TX USA; 22 Department of Pathology, The University of Texas MD Anderson Cancer Center, Houston, TX USA; 23 Department of Clinical Cancer Prevention, The University of Texas MD Anderson Cancer Center, Houston, TX USA; 24 The Francis Crick Institute, London, UK; 25 Cancer Genome Project, Wellcome Trust Sanger Institute, Hinxton, UK; 26 Bioinformatics Division, Walter and Eliza Hall Institute of Medical Research, Parkville, VC Australia; 27 School of Mathematics and Statistics, The University of Melbourne, Melbourne, VC Australia; 28 Cancer Genomics Laboratory, The Francis Crick Institute, London, UK; 29 Department of Human Genetics, KU Leuven Leuven, Belgium; 30 Department of Genetics, The University of Texas MD Anderson Cancer Center, Houston, TX USA

**Keywords:** Computational models, Tumour heterogeneity

## Abstract

Single-cell RNA sequencing studies have suggested that total mRNA content correlates with tumor phenotypes. Technical and analytical challenges, however, have so far impeded at-scale pan-cancer examination of total mRNA content. Here we present a method to quantify tumor-specific total mRNA expression (TmS) from bulk sequencing data, taking into account tumor transcript proportion, purity and ploidy, which are estimated through transcriptomic/genomic deconvolution. We estimate and validate TmS in 6,590 patient tumors across 15 cancer types, identifying significant inter-tumor variability. Across cancers, high TmS is associated with increased risk of disease progression and death. TmS is influenced by cancer-specific patterns of gene alteration and intra-tumor genetic heterogeneity as well as by pan-cancer trends in metabolic dysregulation. Taken together, our results indicate that measuring cell-type-specific total mRNA expression in tumor cells predicts tumor phenotypes and clinical outcomes.

## Abstract (web summary)

Total mRNA expression in cancer cells is a marker for disease progression and death.

## Main

Reprogramming of the transcriptional landscape is a critical hallmark of cancer, which accompanies cancer progression, metastasis and resistance to treatment^1,2^. Recent single-cell studies revealed that expansion of cell state heterogeneity in cancer cells arises largely independently of genetic variation^3–9^, bringing new conceptual insights into longstanding topics of cancer cell plasticity^10^ and cancer stem cells^11,12^. Assessing these clinically relevant topics^13,14^ in large patient cohorts, however, has been difficult due to the high cost and sample quality requirements associated with single-cell technologies. As bulk tumor RNA and DNA sequencing data are already available from large patient series with clinical outcomes, in silico approaches to analyze human tissues may expedite our understanding of tumor heterogeneity.

Some features of transcriptional diversity are more easily quantified in bulk tissues than others. For example, previous approaches to build cellular differentiation hierarchies are not suitable for large-scale human tissue studies where the individual cell identify is lost. These approaches also further require known cell-type-specific genetic markers^15^. Single-cell studies recently demonstrated that the total number of expressed genes per cell can be more predictive of cellular phenotype, such as developmental status, than alterations in any specific genes or pathways^16,17^. Total number of expressed genes in single cells enabled insights in tumorigenesis of breast^16^, colon^18^, pancreas^19^ and blood^20^. In bulk tissues, variation in total mRNA amount—that is, the sum of detectable mRNA transcripts across all genes per cell—has been indirectly linked to cancer progression and de-differentiation as a result of *MYC* activation^21,22^ or aneuploidy^23,24^. With current limitations in our knowledge of marker genes across cancers, total mRNA expression per tumor cell may represent a robust and measurable pan-cancer feature that warrants a systematic evaluation in patient cohorts.

Measuring such a feature in human tissues at-scale poses several analytical challenges, as total tumor cell mRNA expression information is masked during standard bulk data analysis, thus requiring deconvolution. Variation in total mRNA transcript levels is removed by routine normalization, together with technical biases, including read depth and library preparation^25–28^. DNA and RNA sequencing data generated from cancer studies contain reads from both tumor and admixed normal cells. Furthermore, copy number aberrations, such as gain or loss of chromosomal copies (that is, ploidy) in tumor cells, affect gene expression through dosage effects^24^.

In this study, building upon prior work in bulk transcriptome deconvolution^29–31^ and in modeling tumor ploidy^32,33^, we created a measure of tumor-specific total mRNA expression (TmS), which captures the ratio of total mRNA expression per haploid genome in tumor cells versus surrounding non-tumor cells. We first scrutinized total mRNA expression using single-cell data from ten patients across four cancer types^34–36^ and then calculated TmS in matching bulk RNA and DNA data from 6,580 patients across 15 cancer types from four large independent cohorts: The Cancer Genome Atlas (TCGA), the International Cancer Genome Consortium (ICGC)^37^, the Molecular Taxonomy of Breast Cancer International Consortium (METABRIC)^38^ and Tracking Non-Small-Cell Lung Cancer Evolution through Therapy (TRACERx)^39,40^. Our analyses revealed that variation in total mRNA expression is a robust and prognostic feature across cancers.

## Results

### Diversity in total mRNA expression across cancer cells

**Fig. 1:**
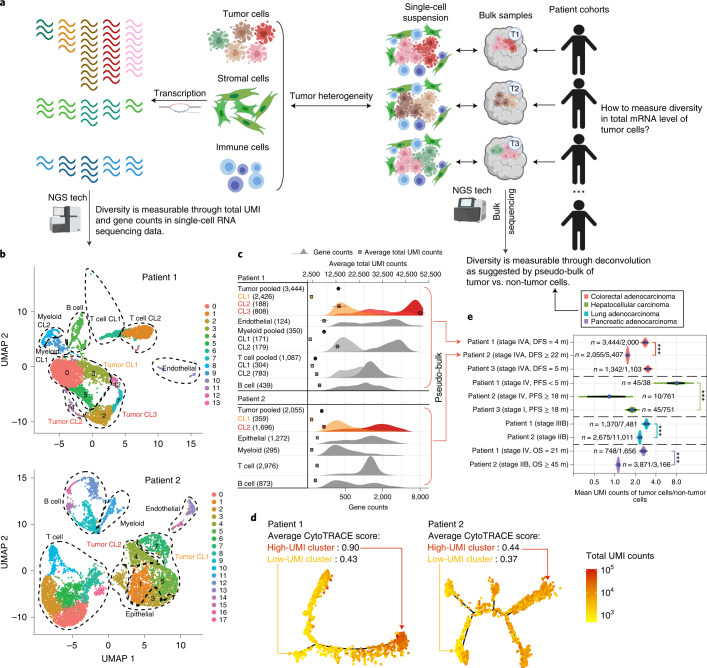
High diversity of total mRNA expression in cancer cells. **a**, Illustration of diversity in total mRNA levels in tumor cells versus other cell types. **b**, UMAP plots of scRNA-seq data from two patients with colorectal cancer. Tumor cell clusters are bolded in both samples. Dashed circles indicate groups of cells that are similar in total UMI and gene counts, which are merged for simplicity. **c**, Distributions of gene counts and total UMI counts by cell type in scRNA-seq data from the two patients shown in **b**. The top *x* axis annotates total UMI counts (with mean and 95% CI). The bottom *x* axis annotates gene count distribution (density). Density curves are colored for tumor cells and shown in grayscale for non-tumor cells. Clusters with higher gene counts are shown in darker shades. Numbers of cells analyzed are indicated in parentheses. Tumor cell clusters are highlighted by the same colors as those in **b**. **d**, Monocle-inferred trajectories for tumor cells from the two patients. Cells on the trees are colored by total UMI counts. Average differentiation scores by CytoTRACE for high-UMI and low-UMI clusters are provided. **e**, Ratios of mean total UMI counts of tumor cells to non-tumor cells (*n* = number of tumor cells / number of non-tumor cells) and 95% CIs in pooled scRNA-seq data (pseudo-bulk) from ten patients with colorectal (*n* = 3, including patients 1 and 2 shown in **b**–**d**), hepatocellular (*n* = 3), lung (*n* = 2) and pancreatic (*n* = 2) cancers. DFS, disease free survival; PFS, progression-free survival; OS, overall survival. The Benjamini–Hochberg-adjusted *P* values for two-sided Wilcoxon rank-sum tests comparing the ratios between patient samples are indicated by asterisks (*P < 0.05, **P < 0.01 and ***P < 0.001). UMAP, uniform manifold approximation and projection. Source data

**Extended Data Fig. 1:**
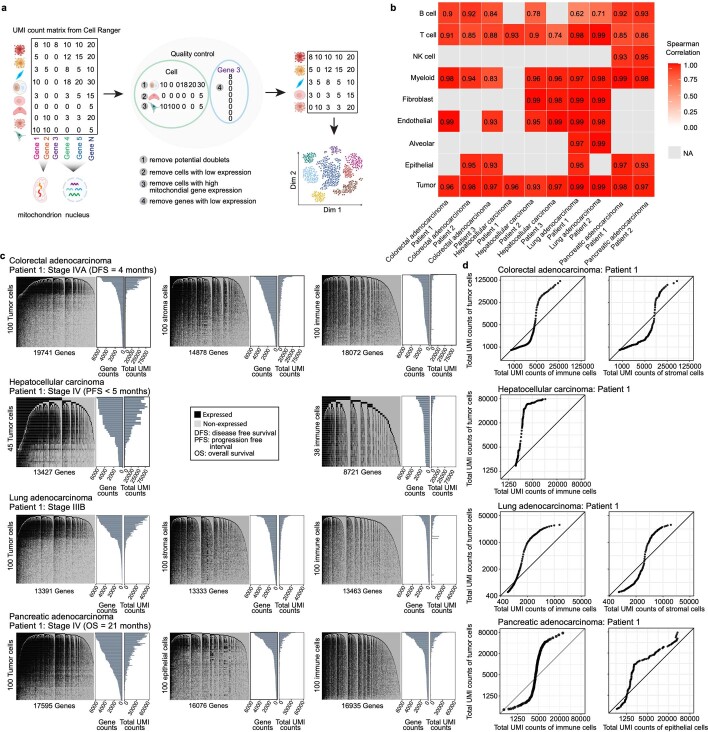
High diversity of total mRNA expression in tumor cells. **a**, Flowchart of scRNA-seq data preprocessing. **b**, Heatmap showing the Spearman correlations between gene counts and total UMI counts across cell types in the ten patient samples. **c**, Illustration of expressed genes in tumor cells (left panels) compared to non-tumor cells: epithelial and stromal cells (middle panels) and immune cells (right panels). The data shown are based on cells randomly selected from each of the four ‘patient 1’ samples with colorectal, hepatocellular, lung and pancreatic cancers, who presented worse prognosis or advanced disease. In each heatmap, expressed genes (UMI count > 0) are shown in black, and non-expressed genes (UMI count = 0) are shown in gray. Cells in the rows and genes in the columns are ordered from high to low by the total numbers of expressed genes and the number of cells with detected expression of each gene, respectively. Barplots provide the corresponding distributions of gene counts and total UMI counts. **d**, Q-Q plots of total UMI counts in tumor cells compared to non-tumor cells for the same four ‘patient 1’ samples that were used as in **c**. For each patient, the log_2_ transformed total UMI counts of immune cells (left) or stromal/epithelial cells (right) are used as the theoretical quantiles, respectively. Source data

**Extended Data Fig. 2:**
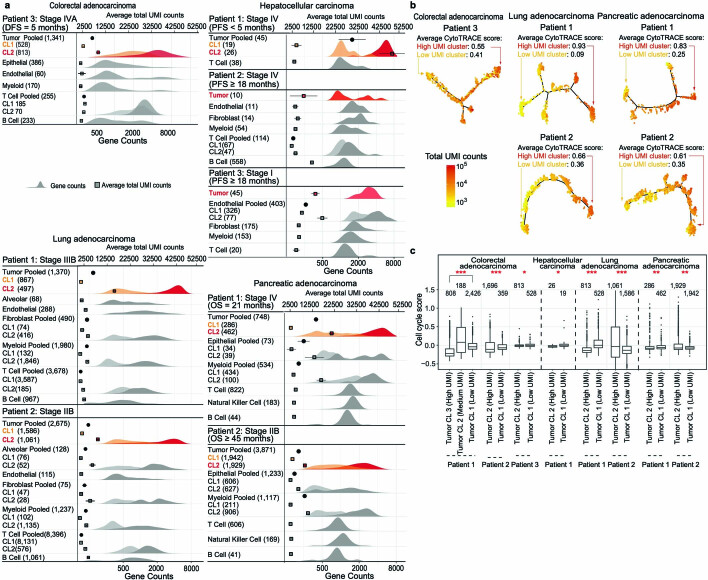
Using total UMI counts and gene counts to measure global gene expression heterogeneity. **a**, Distributions of gene counts and total UMI counts by cell type in scRNA-seq data from eight remaining patients with colorectal, hepatocellular, lung or pancreatic cancers (in relation to Fig. 1). The top x-axis annotates total UMI counts (means and 95% CIs). The bottom x-axis annotates gene count distribution (density). Density curves are shown in color for tumor cells and in grayscale for non-tumor cells. Clusters with higher gene counts are shown in darker shades. Numbers in the parentheses indicate the number of cells analyzed. **b**, Monocle-inferred trajectories for tumor cells from five patients with colorectal, lung and pancreatic cancers. Cells on the trees are colored by total UMI counts. Average differentiation scores by CytoTRACE for high- and low-UMI count tumor cell clusters are labelled. **c**, Distribution of cell cycle scores in tumor cell clusters from eight scRNA-seq patient samples where multiple tumor cell clusters were presented. Cell cycle score is the sum of the S and G2/M scores as estimated by Seurat. *P* values of two-sided Wilcoxon rank-sum tests comparing the cell cycle scores across clusters are indicated by asterisks (* *P* < 0.05, ** < 0.01, *** < 0.001). In the boxplots, whiskers represent the maximum and minimum values of cell cycle scores, the middle line in the box denotes median, and the bounds of the box stand for upper and lower quartiles. Source data

To motivate a model-based quantification of total mRNA expression in bulk tissue, we first analyzed single-cell RNA sequencing (scRNA-seq) data generated from 48,913 cells of ten patients with colorectal (*n* = 3), liver (*n* = 3)^34^, lung (*n* = 2)^35^ or pancreatic (*n* = 2)^36^ cancers (Fig. 1a, Extended Data Fig. 1a, Methods and Supplementary Note 1.1). Total unique molecular identifier (UMI) counts of a cell can be modeled as total mRNA molecule counts multiplied by transcript capture efficiency^41^. Following recent studies^9,16^, demonstrating gene counts as important markers of cellular differentiation, we further propose to use UMI counts to study tumor behavior in human cancers. We observed strong correlations between total UMI counts and gene counts (the number of detectably expressed genes per cell) across all cell types in the ten tumor samples (median Spearman *r* = 0.95 and median absolute deviation (MAD) = 0.04; Extended Data Fig. 1b), in agreement with a prior study in non-cancerous tissues^16^. This supports total UMI counts having a similar utility as gene counts in characterizing tumor cellular phenotype. By investigating the difference of total UMI count distributions in different cell types, we observed a larger variability in tumor cells compared to non-tumor cells (epithelial, stromal and immune cells) (*F*-test for variances, adjusted *P* values < 0.02; Extended Data Fig. 1c,d). Consistent with previous reports^35,42^, we found multiple clusters within tumor and non-tumor cells presenting distinct total UMI and gene counts (Fig. 1b,c, Extended Data Fig. 2a, Methods and Supplementary Note 1.2). High-UMI tumor cells generally demonstrate lower cell cycle activity—that is, non-cycling cells^43^—compared to low-UMI tumor cells (Extended Data Fig. 2c and Supplementary Note 1.2.3). Hence, UMI count is not a surrogate measure for proliferation. Trajectory inference using Monocle^44–46^ shows distinct gene expression states among these clusters (Fig. 1d and Extended Data Fig. 2b). Tumor cells of high-UMI cluster show a less differentiated state^16^ (adjusted *P* values < 0.001; Fig. 1d, Extended Data Fig. 2b and Methods). For instance, in patients with a worse survival outcome (colon, liver and pancreas cancers) or advanced-stage disease (lung cancer), the high-UMI tumor cell clusters present a stem-like cell state as predicted by CytoTRACE^16^ (Fig. 1c,d, Extended Data Fig. 2b and Supplementary Table 1) and demonstrate an enrichment for stemness and the epithelial–mesenchymal transition (EMT) genes (out of 18,617 gene sets^47,48^ investigated; Supplementary Table 2 and Methods). The above observations support the significance of measuring total UMI counts and mRNA content across tumor cells^9,16^.

To support the feasibility of quantifying tumor-specific total mRNA expression in bulk tissues, we pooled the scRNA-seq data to generate pseudo-bulks. As single-cell identity is lost in bulk tissues, we introduce the average total UMI counts per cell for each cell type. To allow for inter-patient comparisons and remove potential technical artifacts still contained in the UMI count measure, we further introduce the ratio of the average total UMI counts for tumor versus non-tumor cells for each sample. Using this bulk-level metric, we observed increased tumor mRNA content in the four patients with advanced disease and worse survival outcomes, as compared to other samples within each cancer type (Fig. 1e; adjusted *P* values < 0.001). This led us to hypothesize that quantification of average tumor-specific total mRNA expression in bulk sequencing data may track tumor phenotype and clinical behavior.

### Estimating tumor-specific total mRNA expression

**Fig. 2:**
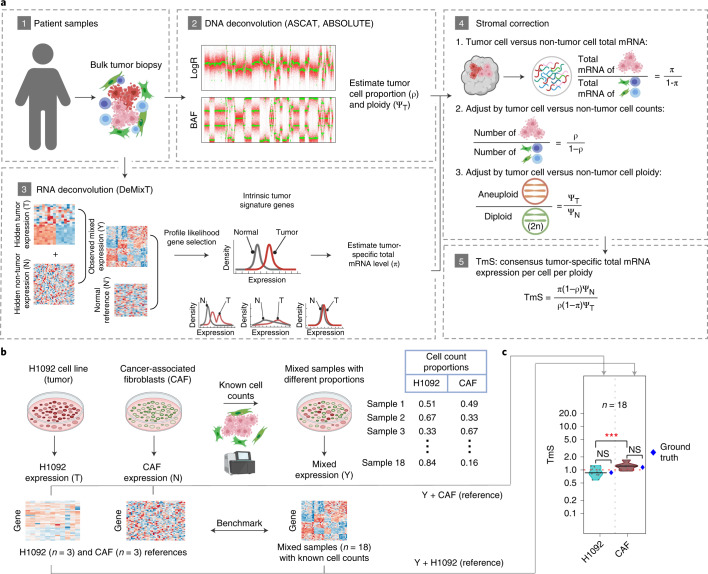
Analysis workflow to measure tumor-specific total mRNA expression and benchmarking. **a**, Calculation of TmS begins with deconvolution using matched DNA sequencing and RNA sequencing data. ASCAT and/or ABSOLUTE are used to estimate tumor purity and ploidy from the DNA sequencing data, whereas DeMixT estimates tumor-specific mRNA proportion from the RNA sequencing. **b**, Benchmarking using bulk RNA sequencing data from in vitro cell lines containing tumor and non-tumor cells, H1092 (human lung cancer) and cancer-associated fibroblasts (CAFs). **c**, Distribution of TmS in 18 mixed cell line samples estimated under two scenarios using DeMixT: (1) three pure CAF samples as known reference and (2) three pure H1092 samples as known reference. The true TmS values for H1092 and CAF are provided in blue dots. They are measured as the ratio of total RNA amount (in ng µl^−1^) in 1 million cells: 0.87 for H1092 and 1.2 for CAF. The median estimates of TmS are (0.86, 1.2), with MADs of (0.24, 0.18) for H1092 and CAF cells, using the other cell line as the baseline. The *P* values for two-sided Wilcoxon rank-sum tests comparing TmS between groups are indicated (not significant (NS): *P* > 0.05; ***P < 0.001). Source data

**Extended Data Fig. 3:**
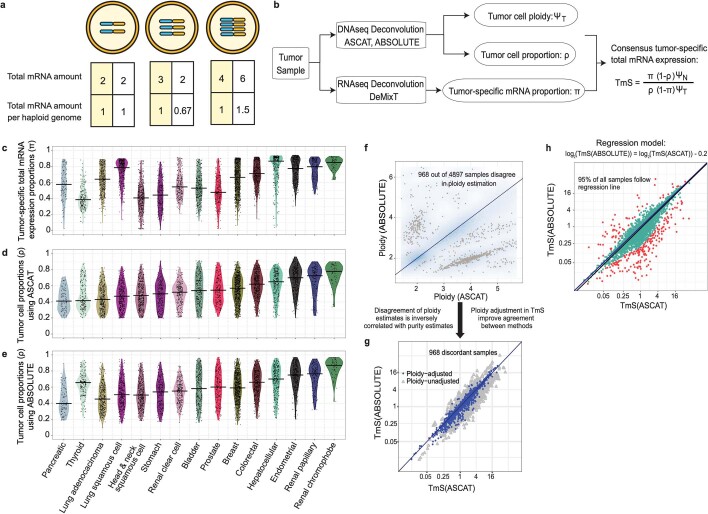
Consensus estimation of TmS from matched RNAseq and DNAseq data in TCGA. **a**, Illustrative relationship between cells, ploidy and mRNA content. Three examples with ploidy of 2, 3, or 4 are given. Under the scenario of linear dosage effects, as shown in the boxes with a yellow background, if cellular total mRNA amounts are 2, 3, and 4, then the ploidy-adjusted, or per haploid genome, total mRNA amount would be 1, 1, and 1, respectively. Under the scenario of dosage compensation, that is, more chromosomal copies but maintaining the same total dose, the second cell has a total mRNA amount of 2 and a per haploid genome value of 0.67. Under the scenario of dosage transgression, *that is*, more chromosomal copies with more dose per copy, the third cell has a total mRNA amount of 6 and a per haploid genome value of 1.5. **b**, Definition of TmS and its analytic pipeline. **c**, Distribution of tumor-specific mRNA proportions estimated by DeMixT across cancer types. **d-e**, Distributions of tumor cell proportions estimated by (**d**) ASCAT or (**e**) ABSOLUTE across cancer types. **f**, Smoothed scatter plot of tumor ploidy estimates from ABSOLUTE *vs*. ASCAT across all samples. Gray points correspond to 968 samples that presented inconsistent tumor ploidy (and purity) estimates between the two methods. **g**, TmS estimates using either ABSOLUTE or ASCAT-derived purity and ploidy estimates with or without ploidy adjustment for the 968 discordant samples from (**f**). Blue and gray points correspond to TmS prior to and after ploidy adjustment, respectively. Ploidy adjustment improved consistency between the ABSOLUTE and ASCAT results. **h**, Scatter plot of TmS calculated using the two methods. A linear regression model was fitted using log_2_(TmS estimated by ABSOLUTE) as the predicted variable and log_2_(TmS estimated by ASCAT) as the predictor variable. Red points are outliers with a Cook’s distance ≥ 4/*n*, where *n* = 5,295 for the total number of TCGA samples. Cyan points are the remaining samples (95%) that showed a good fit for the model and hence their TmS estimates are consistent and robust across two DNAseq deconvolution methods. Source data

**Extended Data Fig. 4:**
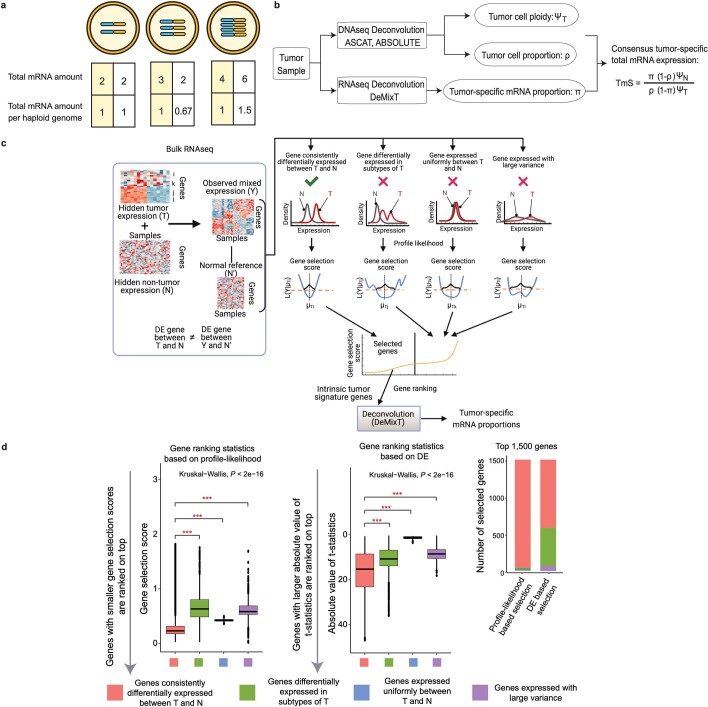
Profile likelihood-based gene selection for RNAseq deconvolution. **a-b**, same as Extended Data Fig. 3a-b. **c**, Illustration of the RNAseq deconvolution workflow with intrinsic tumor signature genes selected using a profile-likelihood based gene selection approach. Three scenarios where genes with undesirable properties are included, leading to large estimation biases, are illustrated with red ‘x’ on top. Their corresponding gene selection scores are expected to be larger than genes with the desirable property for the DeMixT model-based deconvolution (illustrated with a green check on top). Therefore, when genes are ranked based on the gene selection score, as derived using profile likelihoods, selecting the top-ranked genes will reduce the biases in estimating tumor-specific mRNA proportions. **d**, Distributions of gene selection scores across four types of genes in a simulation study (**Supplementary Note 2.2**). For the profile-likelihood based gene selection, genes are ranked from the smallest to the largest score (left). For the DE based gene selection, genes are ranked from the largest to the smallest absolute t-statistics (middle). *P* values of Kruskal-Wallis (one-way ANOVA) test across all four gene groups are shown on top. *P* values of two-sided Wilcoxon rank-sum tests within pairs of gene groups are indicated by asterisks (* *P* < 0.05, ** < 0.01, *** < 0.001). The types of genes among the top 1,500 selected genes are shown (right) for the two rankings. Ideally only genes consistently differentially expressed between tumor (*T*) and normal (*N*), annotated in red, should be selected, corresponding demonstrating the lowest values in both panels as compared to genes annotated in other colors. This is achieved by the profile-likelihood method but not the DE method. In the boxplots, whiskers represent the maximum and minimum values of gene selection scores, the middle line in the box stands for median, and the bounds of the box stand for upper and lower quartiles. Source data

To quantify the average tumor-specific total RNA expression across a large number of patient samples, we employ three steps in a sequential deconvolution of matched DNA/RNA sequencing data (Fig. 2a, Methods and Supplementary Note 2.1). (1) We estimate the ratio of total RNA expression between two cellular populations, tumor versus non-tumor cells, to cancel out technical effects. This ratio can be estimated as an odds of transcript proportions (*π*), based on a set of robust intrinsic tumor signature genes. (2) We divide the total RNA expression by their relative cell fractions to calculate a per-cell total RNA content for tumor and non-tumor cells separately. This step requires matched DNA data from which the tumor cell proportion—that is, purity (*ρ*)—as well as ploidy (Ψ_*T*_) are estimated. (3) We divide the above metric by ploidy (for both components), thereby adjusting for the dosage effect of chromosomal copies on gene expression. We thus calculate our final quantitative metric: the per-cell, per-haploid genome total RNA expression for tumor—that is, TmS—as $[\pi \left( {1 - \rho } \right)\Psi _N]/[\rho \left( {1 - \pi } \right)\Psi _T]$. The parameters *ρ* and Ψ_*T*_ can be derived using DNA sequencing or single-nucleotide polymorphism (SNP) array data (for example, using ASCAT^32^, ABSOLUTE^33^ or Sequenza^49^; Extended Data Fig. 3a–h and Methods). The parameter *π* can be derived using RNA sequencing or microarray data (for example, using DeMixT^31^). A major challenge in estimating *π* is that the unobserved tumor-specific and non-tumor-specific expression levels of many genes present multimodal distributions across tumor subtypes, which would introduce large estimation biases (Extended Data Fig. 4a–d and Methods). To address this issue and obtain more robust *π* estimates, we introduce a profile likelihood of the DeMixT model to rank genes for each study cohort and identify top-ranked genes as an intrinsic tumor signature gene set, where genes follow a unimodal distribution with low variance across the hidden tumor component and are differentially expressed from the non-tumor component (Extended Data Fig. 4c,d, Methods and Supplementary Note 2.2). Simulation studies confirmed more robust *π* estimation when only the intrinsic tumor signature genes are used to perform transcriptome deconvolution (Supplementary Note 2.2).

**Extended Data Fig. 5:**
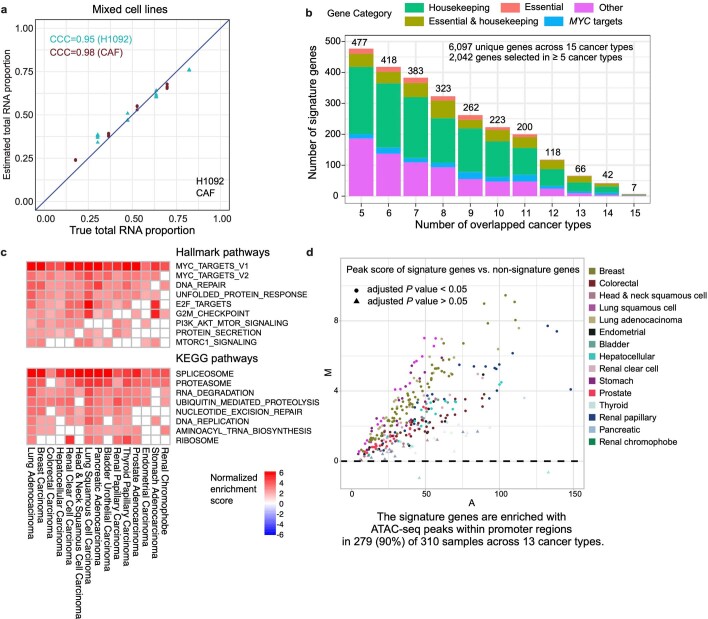
Validating the TmS measure through benchmarking and evaluating the biological relevance of intrinsic tumor signature genes in TCGA. **a**, Total mRNA proportion estimation for H1092 and CAF using DeMixT in the benchmarking study (*n* = 18). The concordance correlation coefficient (CCC) for two variables x (true tumor-specific RNA proportions) and y (estimated tumor-specific RNA proportions) is expressed as $\frac{{2\rho \sigma _x\sigma _y}}{{\sigma _x^2 + \sigma _y^2 + (\mu _x - \mu _y)^2}}$, where *μ* and *σ*^2^ represent the mean and variance, and *ρ* is the Pearson correlation coefficient. **b**, Histogram of the number of overlapping genes across cancer types and their annotation categories. The y axis represents the total number of genes and the x axis represents the number of cancer types for which a gene was selected. **c**, Heatmap of normalized enrichment scores of top cancer hallmark pathways and KEGG pathways. Only pathways with a BH adjusted *P* value < 0.05 are colored. **d**, M-A plot comparing ATAC-seq peak scores of intrinsic tumor signature genes (signature) *vs*. other genes (non-signature) from matched tumor samples in each cancer type. Samples above the dashed line have higher ATAC-seq peak scores in intrinsic tumor signature genes compared to those in non-signature genes. Samples with BH adjusted *P* values < 0.05 from per-sample permutation tests are shown as circles. Source data

We benchmarked the performance of TmS estimation using total RNA sequencing data generated from mixed cell populations with known proportions^31^, resulting in accurate separation of the H1092 lung cancer cell transcriptome from that of cancer-associated fibroblasts (CAFs) (Fig. 2b,c, Supplementary Table 3, Extended Data Fig. 5a and Methods).

### TmS as a measure of tumor-specific total mRNA expression

**Fig. 3:**
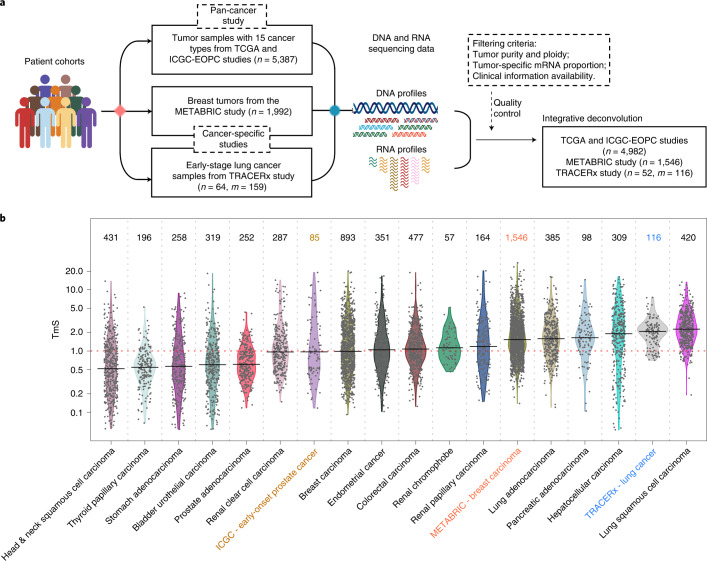
Estimation of tumor-specific total mRNA expression in bulk sequencing data. **a**, Diagram for the TmS calculation in TCGA, ICGC-EOPC, METABRIC and TRACERx datasets. The number of patients is denoted by *n*. When there are more than one tumor sample for each patient, the number of tumor samples is denoted by *m*. **b**, Distribution of TmS in 6,644 tumor samples from 6,580 patients across 15 cancer types in TCGA, ICGC-EOPC, METABRIC and TRACERx. The number of tumor samples for each cancer type is indicated above each violin plot. Source data

We calculated TmS across 15 TCGA cancer types, the early-onset prostate cancer (EOPC) cohort from the ICGC, the METABRIC study and the TRACERx study (Fig. 3a,b, Methods and Supplementary Note 2.3). The intrinsic tumor signature genes selected for TmS estimation largely overlap across cancers (Extended Data Fig. 5b) and are enriched in housekeeping, essential^50,51^, cancer hallmark^47^ and transcriptional regulation pathway genes (RNA splicing and degradation and protein degradation; Extended Data Fig. 5c). As expected, selected genes also demonstrated increased chromatin accessibility^52^ versus non-selected genes (Extended Data Fig. 5d). These pan-cancer consistencies support the biological underpinning of TmS as well as our profile-likelihood-based approach for selecting stably and differentially expressed genes in tumor cells. Moreover, all cancer types studied demonstrated a much wider TmS range in patient samples compared to the variance of TmS derived using a homogeneous tumor cell population in the benchmarking study (Fig. 2c versus Fig. 3b; *F*-test for variances, adjusted *P* values < 0.001 for all cancer types). These findings suggest that considerable variation in tumor-specific total mRNA expression exists among patient samples (Fig. 3b, Supplementary Table 4, Methods and Supplementary Note 2.3).

**Extended Data Fig. 6:**
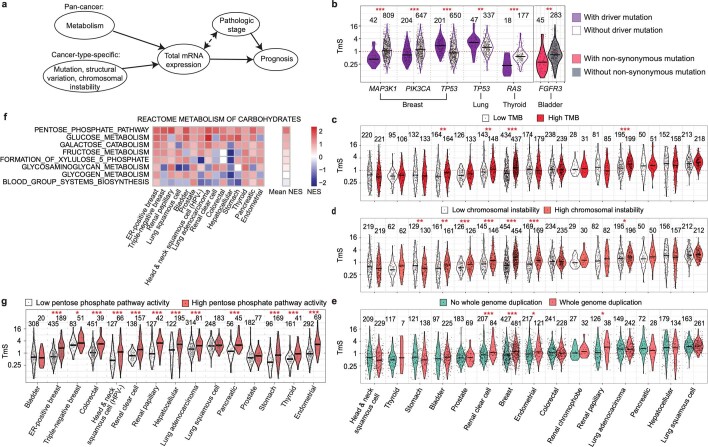
TmS is associated with tumor genomic features and metabolic pathway activities across cancer types. **a**, Contributors to tumor-specific total mRNA expression. **b**, Distributions of TmS for TCGA samples with or without specific mutations in six cancer-gene pairs. The number of samples is indicated on the top. We performed an agnostic association analysis of TmS with all non-synonymous mutations (32,894 cancer-gene pairs, using logistic regression models), and concurrently a driver mutation-specific association analysis of TmS (24 cancer-gene pairs). We find 5 overlapping pairs out of 6 statistically significant pairs produced from each interrogation (BH adjusted *P* values < 0.01). The additional pair found through the agnostic search (*FGFR3* in bladder carcinoma in TCGA) was not identified in the driver mutation analysis due to a limited sample size. These associations in breast, lung, thyroid, and bladder cancers show that TmS can capture changes in tumor phenotypes induced by driver mutations in a cancer type-specific manner. Our observation also supports previous findings that the same driver mutations may not have the same prognostic effect across cancers, and their effects may be modified by additional tumor and/or treatment-related factors. **c-e**, Distribution of TmS for patient samples with (**c**) high or low tumor mutation burden (TMB); (**d**) high or low chromosomal instability score; (**e**) with or without a whole genome duplication event. Patient groups are categorized as high *vs*. low based on the median values of TMB and chromosomal instability scores in (**c**) and (**d**) respectively. **f**, Heatmap of normalized enrichment scores (NES) of Reactome metabolism of carbohydrates pathways across 15 cancer types in TCGA. Pathways are ordered by the mean NES across 15 cancer types, from high to low. **g**, Distribution of TmS for patient samples with high or low for pentose phosphate pathway activity, where patient groups are defined by hierarchical clustering of expression levels from 13 genes. For **b**-**d** and **g**, the BH adjusted *P* values for two-sided Wilcoxon rank-sum tests comparing TmS between corresponding groups are indicated by asterisks (* *P* < 0.05, ** < 0.01, *** < 0.001). Source data

To serve as a meaningful measure, we expect TmS to capture alterations in tumor-specific total mRNA expression attributable to a variety of interacting biological processes (Extended Data Fig. 6a). We evaluated biological correlates of tumor-specific total mRNA expression across 4,982 patients from 15 cancer types in TCGA. Because *MYC* dysregulation is a known mechanism of global transcriptional amplification across cancers, we first evaluated the relationship between TmS and *MYC* expression and found a positive correlation in several cancer types^53^, including breast carcinoma and renal papillary carcinoma (Spearman *r* = 0.17 and 0.21, respectively; Supplementary Note 2.3.2). We further examined genetic alterations, which may affect transcriptional activity, including driver mutations, tumor mutation burden (TMB), chromosomal instability (CIN) and whole-genome duplication (WGD) status (Methods and Supplementary Note 2.3.2). Significant associations were identified in some cancer types, suggesting that these genetic features may contribute to tumor-specific total mRNA expression in certain cancers but are not pan-cancer determinants (Extended Data Fig. 6b–e and Supplementary Note 2.3.2). Although we did not identify other pan-cancer genetic determinants of TmS, we found a pervasive upregulation of metabolic pathways in high-TmS samples across cancers. Specifically, the pentose phosphate pathway is the most frequently upregulated (significant in 12 of 15 cancers), followed by the glucose metabolism pathway (significant in seven of 15 cancers) (Extended Data Fig. 6f,g), in line with their roles in nucleotide synthesis and tumor metabolic reprogramming^54,55^, respectively. These findings further validate the TmS metric in measuring tumor-specific total mRNA expression and support that the large inter-patient variation observed in TmS may be an important feature of tumor cells.

### Tumor cell total mRNA expression refines prognostication

**Fig. 4:**
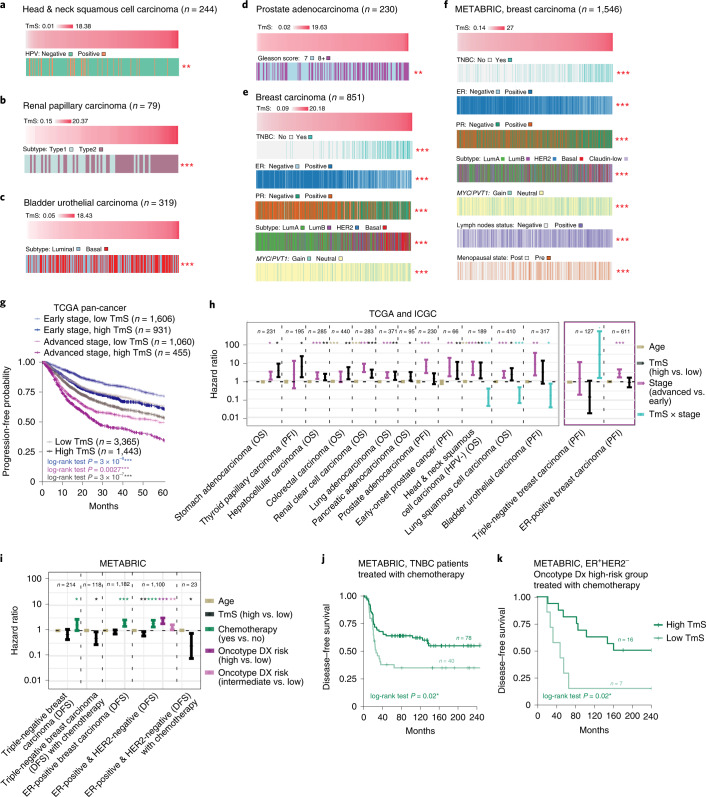
TmS is associated with known prognostic characteristics and refines prognostication in addition to stage. **a**–**f**, Clinicopathologic annotations for TCGA head and neck (**a**); TCGA renal papillary (**b**); TCGA bladder urothelial (**c**); TCGA prostate (**d**); TCGA breast (**e**); and METABRIC breast (**f**) cancers. Receptor status is indicated as follows: ER, estrogen; PR, progesterone; TNBC, triple-negative. Tumor samples are ordered by TmS from low to high. Benjamini–Hochberg-adjusted *P* values for Kruskal–Wallis tests comparing TmS across clinicopathologic subgroups are indicated by asterisks. For *MYC*/*PVT1* copy number status, ‘Gain’ indicates either *MYC* or *PVT1* amplification, and ‘Neutral’ indicates that no copy number alterations were detected. **g**, Kaplan–Meier curves of PFI for TCGA samples. Gray lines denote summary Kaplan–Meier curves of patients with high versus low TmS across all cancer types. Kaplan–Meier curves are further grouped into four groups by TmS and pathologic stage. *P* values of log-rank tests between high- versus low-TmS groups are indicated by asterisks. **h**, Forest plot of HRs (center points) and 95% CIs (error bars) of multivariate Cox proportional hazard models for OS or PFI in TCGA. Models are adjusted for age, TmS (high versus low), stage (advanced versus early) as well as an interaction term of TmS × stage, where applicable (see details in Supplementary Table 5). **i**, Forest plot of HRs (center points) and 95% CIs (error bars) of multivariate Cox proportional hazard models with age, TmS (high versus low), chemotherapy (yes versus no), Oncotype Dx risk classification (high versus intermediate versus low) as predictors for DFS in METABRIC (see details in Supplementary Table 7). For **h** and **i**, *P* values of two-sided Wald tests for the covariates are indicated by asterisks. Kaplan–Meier curves of DFS grouped by TmS (high versus low) for METABRIC TNBC (**j**) and ER^+^HER2^−^ (**k**) patients treated with chemotherapy. *P* values of log-rank tests between high- versus low-TmS groups are indicated by asterisks. For all *P* values, significance levels are denoted as follows: *P < 0.05, **P < 0.01 and ***P < 0.001. HPV, human papillomavirus. Source data

To understand the significance of TmS variation across patient samples, we first examined TmS in the context of histopathologic and molecular subtypes across cancers. Although many tumor subtypes have been described across cancers, we specifically examined five cancers where these subtypes have been most unequivocally shown to harbor differential biology and clinical significance. We observed consistent trends across subtypes of head and neck squamous cell carcinoma, renal papillary carcinoma^56^, bladder urothelial carcinoma^57–59^ and prostate adenocarcinoma, where prognostically favorable subtypes are enriched in tumors with lower TmS and vice versa (Fig. 4a–d and Methods). Similarly, in breast carcinoma, triple-negative receptor status is associated with higher TmS, in keeping with this subtype’s known propensity for aggressive behavior (TCGA: adjusted *P* = 5 × 10^−36^, Fig. 4e; METABRIC: adjusted *P* = 9 × 10^−28^, Fig. 4f). However, we found that TmS is not a surrogate for histopathologic or molecular subtype, tumor cellular proliferation or pluripotency genes^60^ (Supplementary Note 2.3.2.5), suggesting that variation in TmS captures unique aspects of tumor biology that affects aggressiveness.

**Extended Data Fig. 7:**
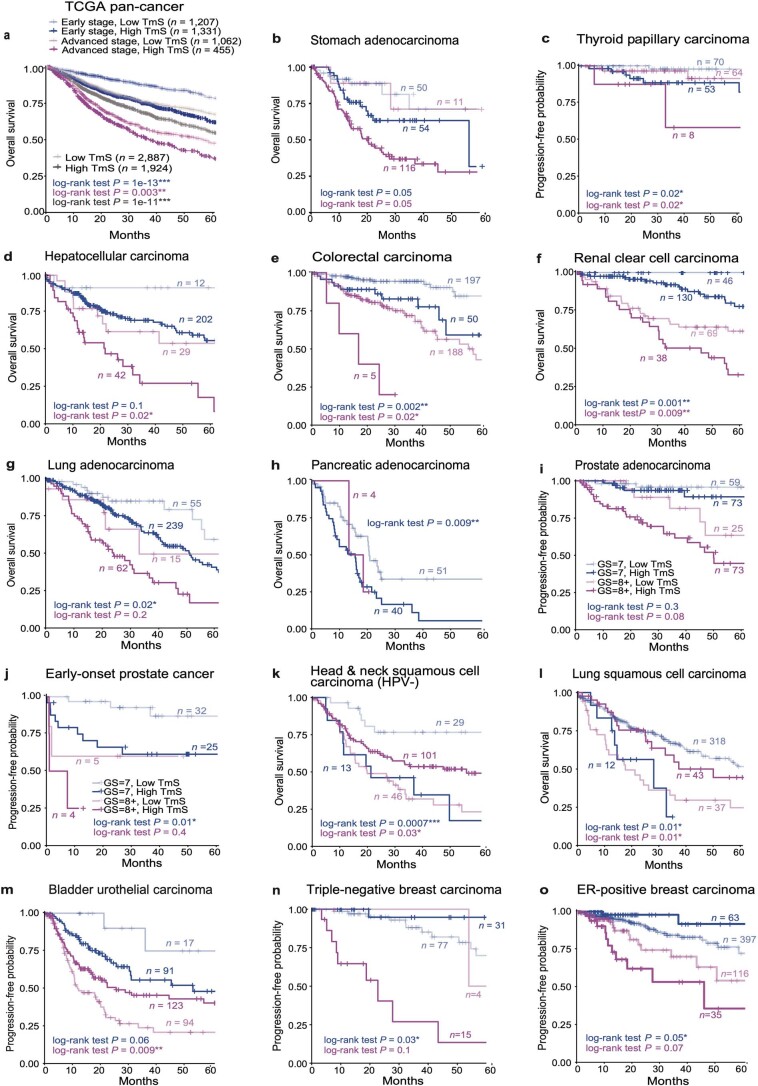
TmS refines prognostication on pathological stages. **a**, KM curves of OS for TCGA pan-cancer. Gray lines denote summary KM curves of patients with high *vs*. low TmS across all cancer types. KM curves are further grouped by TmS and pathological stages into four groups. *P* values of log-rank tests between high *vs*. low TmS groups are indicated by asterisks (* *P* < 0.05, ** < 0.01, *** < 0.001). **b-o**, KM survival curves for individual cancer types. Source data

**Extended Data Fig. 8:**
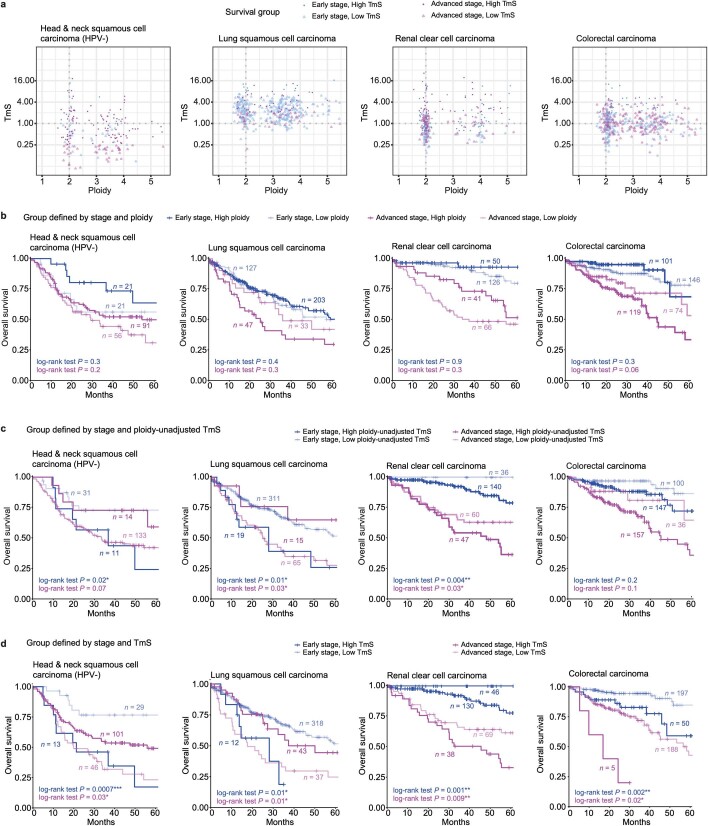
Prognostication using ploidy or ploidy-unadjusted TmS on pathological stages. **a**, Scatter plots of TmS (y axis) *vs*. tumor ploidy (x axis) for samples from TCGA patient cohorts with head-and-neck squamous cell carcinoma (HPV negative), lung squamous cell carcinoma, renal clear cell carcinoma, and colorectal carcinoma. The samples were grouped into high *vs*. low TmS within early or advanced pathological stages, with different groups shown in distinct colors. TmS shows no correlation with tumor ploidy, with Spearman correlation coefficients *r* = −0.12, 0.01, 0.08 and −0.02 for the four cancer types. **b**, KM survival curves of OS in four cancer types according to patient groups defined by ploidy and stage. We grouped patients into high *vs*. low ploidy based on a cutoff of 2.5 within early or advanced pathological stage. **c**, KM survival curves of overall survival in four cancer types over patient groups defined by ploidy-unadjusted TmS and stage. **d**, KM survival curves of OS in four cancer types for patient groups defined by TmS and stage. *P* values of log-rank tests between pairs of patient groups are shown with matching colors and are indicated by asterisk (* *P* < 0.05, ** < 0.01, *** < 0.001). Source data

To further evaluate the potential utility of TmS to enable clinically relevant patient stratification, we examined the association of TmS with survival outcomes in TCGA and ICGC-EOPC (Methods and Supplementary Notes 3.1 and 3.2). In pan-cancer analyses, high TmS is associated with reduced overall survival (OS) and progression-free interval (PFI) (Fig. 4g, Extended Data Fig. 7a and Supplementary Table 5), which is robust to sample size differences across cancer types (Supplementary Note 3.2). TmS is independent of other clinical characteristics, including age and sex (Supplementary Note 2.3.2.5). Although TmS correlates with tumor-node-metastasis (TNM) stage in some cancer types, this relationship is not consistently observed across cancers (Supplementary Note 2.3.2.5). After feature selection and adjusting for known prognostic characteristics, including tumor subtype, stage and age (Methods), TmS was independently significantly associated with survival outcomes in all evaluable cancer types, except for estrogen receptor (ER)-positive breast carcinoma (Fig. 4h, Extended Data Fig. 7b–o, Supplementary Table 5 and Supplementary Notes 3.1 and 3.2). This association is retained, but weaker, when genome ploidy adjustment of TmS is omitted (Extended Data Fig. 8).

**Extended Data Fig. 9:**
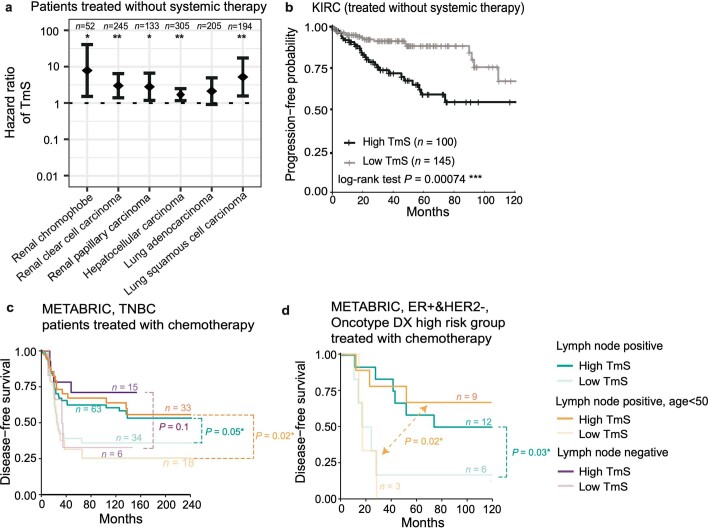
TmS refines prognostication in cancer patients with and without systemic therapy. **a**, Forest plot of hazard ratios and 95% of CIs of TmS as predictor in patients treated without systemic therapy across 6 TCGA cancer types. *P* values of two-sided Wald tests are indicated by asterisks (* *P* < 0.05, ** < 0.01, *** < 0.001). **b**, KM curves of PFI for renal clear cell carcinoma patients without systemic therapy. **c**, KM curves of DFS for METABRIC triple negative breast cancer patients who are treated with chemotherapy. KM curves are further grouped by TmS, Lymph node status and age into six groups. **d**, KM curves of DFS for METABRIC estrogen receptor (ER) positive and human epidermal growth receptor-2 (HER2) negative breast cancer patients who are classified as high risk by Oncotype Dx risk score and treated with chemotherapy. KM curves are further grouped by TmS and age under 50. For **b**, **c** and **d**, *P* values of log-rank tests between pairs of patient groups are shown with matching colors and are indicated by asterisk (* *P* < 0.05, ** < 0.01, *** < 0.001). Source data

When patients are stratified by TNM stage classification, the prognostic effect of TmS differs between early (I/II) and advanced (III/IV) stage. Because early-stage versus advanced-stage tumors are generally treated using different therapeutic modalities, we hypothesized that the prognostic effect of TmS is modified by treatment. Given that the TCGA and ICGC studies did not consistently include chemotherapy and radiotherapy information^61^, we identified a cohort of patients where chemotherapy and/or radiotherapy are generally not indicated (https://www.nccn.org/guidelines/category_1; Supplementary Table 6). Among these patients treated without systemic therapy, high TmS remains associated with worse PFI (Extended Data Fig. 9a,b).

In METABRIC, where treatment information is well-annotated, high TmS is associated with improved disease-free survival (DFS) in patients with early-stage triple-negative breast carcinoma (TNBC) treated with chemotherapy (*n* = 118, hazard ratio (HR) = 0.5, 95% confidence interval (CI): 0.28, 0.89, log-rank *P* = 0.02; Fig. 4i,j, Extended Data Fig. 9c and Supplementary Table 7). This is consistent with prior observations that high-risk breast tumors may respond better to chemotherapy^62,63^. This inversed relationship between high TmS and improved survival can be appreciated across all patients with TNBC in METABRIC with marginal significance (*n* = 214, HR = 0.7, 95% CI: 0.44, 1.12, log-rank *P* = 0.1; Fig. 4i and Supplementary Table 7), likely reflecting that most of these patients received systemic therapy. The same inversed relationship is observed in TNBC in TCGA (Fig. 4h and Supplementary Table 5).

Furthermore, in METABRIC, we found that high TmS is associated with improved DFS for patients with ER^+^HER2^−^ breast cancer, after adjusting for chemotherapy and Oncotype Dx risk status (*n* = 1,100, HR = 0.74, 95% CI: 0.60, 0.91, log-rank *P* = 0.004; Fig. 4i and Supplementary Table 7). Oncotype Dx risk score is routinely used clinically as a biomarker to estimate the risk of ER^+^HER2^−^ tumors^64^. Within patients who were classified as high risk by Oncotype Dx and treated with chemotherapy, high TmS remains associated with better survival (*n* = 23, HR = 0.25, 95% CI: 0.08, 0.77, log-rank *P* = 0.02; Fig. 4i,k and Extended Data Fig. 9d). Patients with low TmS appeared to not have benefited from chemotherapy, suggesting the potential need for alternative therapy for this subgroup of patients. In summary, our findings suggest a unique utility of TmS in identifying and stratifying high-risk patients for treatment selection in breast cancer, which may be expandable to other cancer types.

### Intra-tumor and inter-tumor heterogeneity in total mRNA expression

**Fig. 5:**
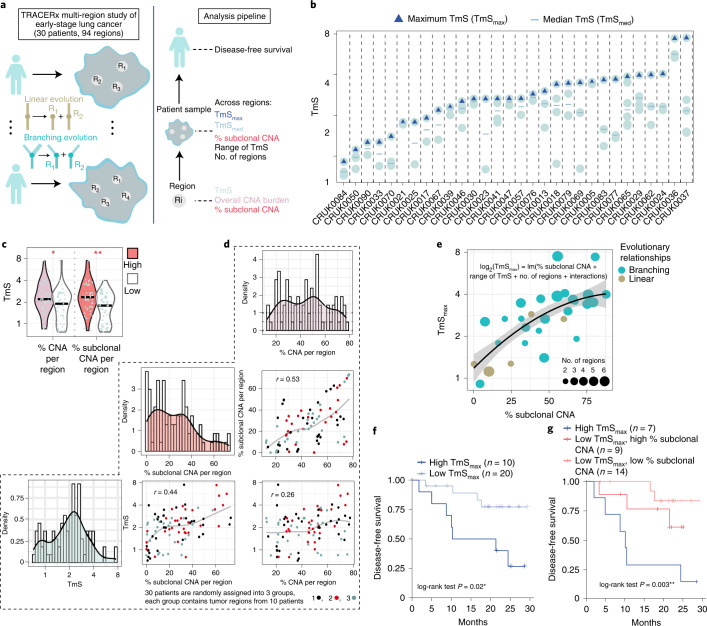
Regional estimation of TmS identifies spatial heterogeneity and refines prognostication in early-stage lung cancer. **a**, Illustration of the TRACERx multi-region study and a multi-level analysis pipeline. **b**, Distribution of TmS for 94 tumor regions from 30 TRACERx patients with at least two regions sampled. Blue triangles denote the maximum TmS for each patient. Blue ‘-’ denotes the median TmS for each patient. **c**, Distributions of TmS per region with high or low % CNA burden (left) and % subclonal CNA per region (right). The number of regions is 47 for each group. Benjamini–Hochberg-adjusted *P* values of two-sided Wilcoxon rank-sum tests are indicated by asterisks. **d**, Pairwise scatter plots and histograms of % CNA, % subclonal CNA and TmS per region across 94 regions. Different colors annotate three randomly assigned patient groups, demonstrating that the correlation between TmS and % subclonal CNA per region is not driven by a subset of patients. Spearman correlation coefficient *r* values are shown, and the gray lines represent a LOESS fit. **e**, Scatter plot showing TmS_max_ versus total % subclonal CNA in each patient (*n* = 30). The regression line and its 95% confidence band are colored in black and gray, respectively. Patients are colored by the evolutionary relationship. **f**, Kaplan–Meier survival curves of DFS stratified by TmS_max_. **g**, Kaplan–Meier survival curves of DFS stratified by both TmS_max_ and % subclonal CNA. *P* values are obtained by log-rank tests between high- versus low-TmS groups. For all *P* values, significance levels are denoted as follows: *P < 0.05, **P < 0.01 and ***P < 0.001. Source data

Intra-tumor heterogeneity serves as a reservoir for tumor evolution, treatment resistance and progression. Although intra-tumor heterogeneity can be identified using scRNA-seq (Fig. 1b,c and Extended Data Fig. 1a), the evolutionary relationships of tumor cell subpopulations cannot be readily inferred from scRNA-seq data alone. We, therefore, used TRACERx, a multi-region study of early-stage lung cancer evolution^39^, to evaluate the potential utility of TmS for quantifying transcriptomic intra-tumor heterogeneity (Fig. 5a).

**Extended Data Fig. 10:**
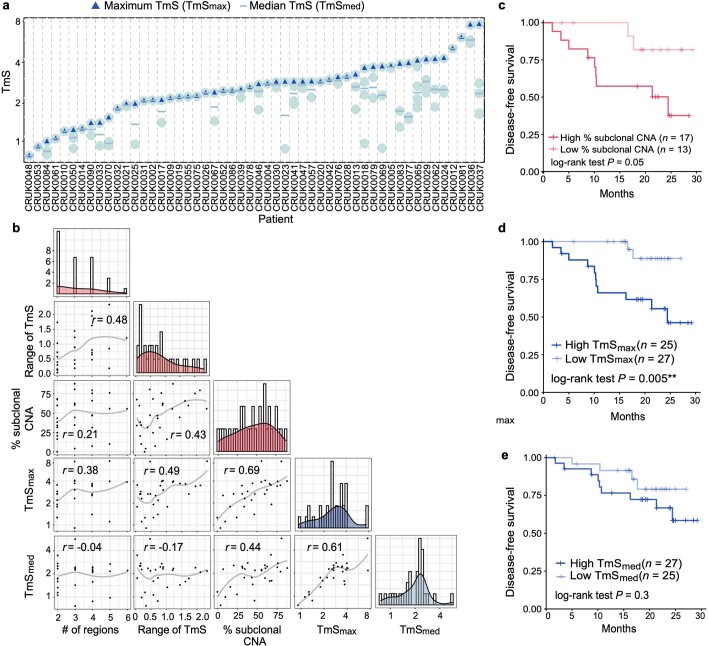
Regional TmS identifies spatial heterogeneity and refines prognostication in patients with early-stage lung cancer. **a**, Distribution of TmS values for 116 tumor regions from 52 patients of the TRACERx study. Blue triangles denote the maximum TmS for a patient. Blue ‘-‘ denote the median TmS for a patient. **b**, Pairwise scatter plots and histograms of number of regions, range of TmS, % subclonal CNA, maximum of TmS across regions (TmS_max_), and median of TmS across regions (TmS_med_) per patient. The number of evaluated patients with at least 2 regions is 30. Spearman correlation coefficient *r*’s are shown, and the gray lines represent a loess fit. **c**, KM survival curves of DFS for the 30 patients stratified by % subclonal CNA: high versus low. **d-e**, KM survival curves of DFS for all 52 patients stratified into two groups by TmS_max_ (**d**) and (**e)** TmS_med_, respectively. *P* values obtained by log-rank tests between high *vs*. low TmS groups are indicated by asterisks (* *P* < 0.05, ** < 0.01, *** < 0.001). Source data

We calculated TmS using matched whole-exome sequencing (WES) and RNA sequencing data generated from 116 evolutionarily and spatially distinct regions across 52 patients, 30 of whom have two or more regions sampled (94 regions total) (Figs. 3b and 5b and Extended Data Fig. 10a). Subclonal copy number alterations (CNAs) and phylogenetic relationships of cancer subclones have been determined for these regions^39^. We first investigated the relationship between TmS and subclonal CNA, as determined by TRACERx. Across all 94 regions, TmS correlates better with the fraction of CNAs that are subclonal—that is, CNAs identified in only some regions of the tumor—than the fraction of the genome affected by CNA events (difference in Spearman *r* = 0.20, 95% CI: 0.04, 0.37; Fig. 5c,d and Methods). This suggests that TmS tracks ongoing chromosomal instability^65^, reflecting intra-tumor heterogeneity, rather than the total CNA burden. To summarize across regions, we calculated the median and maximum of TmS, TmS_med_ and TmS_max_, as well as the range of TmS (maximum – minimum TmS across regions) per patient (Extended Data Fig. 10a). As expected, TmS_med_ is highly correlated with TmS_max_ across patients (Spearman *r* = 0.61). However, TmS_max_ shows a higher correlation with the total fraction of subclonal CNAs than TmS_med_ or the range of TmS (Spearman *r* = 0.69 versus 0.44 and 0.49; Extended Data Fig. 10b). Furthermore, TmS_max_ can be best explained, in a multiple linear regression, by the total fraction of subclonal CNAs (coefficient = 2.9, *P* < 0.001, regression goodness-of-fit *R*^2^ = 0.7; Fig. 5e, Methods and Supplementary Note 3.3). Additionally, in a logistic regression model, a smaller range of TmS per patient is predictive of linear evolutionary relationship between the regions sampled (area under the curve (AUC) = 0.83; Supplementary Note 3.3). These findings support the utility of measuring TmS per tumor region to quantify transcriptomic intra-tumor heterogeneity and, more specifically, its variation over evolutionary relationships.

Following the multi-cohort single-sample analyses, we hypothesized that the tumor region harboring subclones with highest TmS is most predictive of prognosis in early-stage lung cancer. Confirming this hypothesis, we observed that high TmS_max_ is associated with worse DFS (log-rank *P* = 0.02; Fig. 5f), which is also consistent with our findings from TCGA in lung cancer. Patient stratification using both TmS_max_ and fraction subclonal CNA allows further discrimination of clinical outcomes (log-rank *P* = 0.003; Fig. 5g), with a Cox regression concordance index of 0.75 (TmS_max_ and fraction subclonal CNA) versus 0.66 (fraction subclonal CNA only; Extended Data Fig. 10c). When 22 additional patients with a single region per tumor are included, high TmS_max_ remains associated with higher risk of recurrence or death (log-rank *P* = 0.005; Extended Data Fig. 10d). High TmS_med_ shows a similar trend, although not statistically significant (log-rank *P* = 0.3; Extended Data Fig. 10e).

In summary, variation in tumor total mRNA expression appears to be synergistic with recently acquired DNA alterations during evolution. A multi-region design, by measuring average tumor-specific total mRNA expression for each region, can improve the resolution of the TmS quantification, thus enabling assessment of transcriptomic intra-tumor heterogeneity and further prognostication of early-stage lung cancer.

## Discussion

Our study identifies TmS, a robust and measurable feature of tumor phenotype, from bulk tumor tissues. TmS is clinically and molecularly relevant across cancer types. Although single-cell technology can depict tumor cell populations with distinct gene expression states (a microscopic view), questions remain on how these populations coexist and interact to affect patient outcomes^10^. Average signals across all tumor cells summarize the magnitudes and fractions of each tumor cell population. It is known, mathematically, that in distributions such as Poisson and Exponential, the mean and the variance are highly correlated. In such scenarios, the average measures provide essential information for the entire distribution. Here we demonstrate that, indeed, the average value of tumor-specific total mRNA expression is informative when used to investigate both inter-tumor and intra-tumor heterogeneity and is also predictive of clinical outcomes in patients with cancer (a macroscopic view).

Using the lens of diversity in total mRNA expression, our study sheds light on cancer cell plasticity, previously evaluated in only a few tumors or in model systems^14^. To achieve a pan-cancer analysis that complements single-cell-based studies^16,18–20^, we developed and calculated TmS, as an integrative RNA and DNA deconvolution metric for bulk tissues, in 6,580 patient samples from 15 cancer types. Association of TmS with transcriptional regulators, genetic features, metabolism as well as evolutionary relationships supports a consistent and biologically meaningful measurement of a bulk-level feature of tumor phenotype. We further report the ability of TmS to refine prognostication within each of the 12 cancer types with staging information and sufficient sample size.

Although high tumor cell total mRNA expression is generally associated with high-risk disease, clinical context remains important to evaluate its prognostic implications, as the direction of the prognostic effect was inverted by stage in four of 12 cancer types examined. Given that different tumor types and stages are often treated using distinct modalities, the inverted effect may, in part, be underpinned by a differential response of tumors with low versus high total mRNA expression to treatment. We validated the inverted effect in breast cancer subtypes in TCGA using the METABRIC cohort study in which treatment information was well-documented. Our findings are consistent with prior reports describing subsets of patients with aggressive cancer subtypes that respond favorably to systemic therapy^63,66,67^. Identifying which patients may benefit from specific systemic therapies remains a challenge, and TmS may serve to identify these patients as well as others requiring alternative treatments. Additional studies incorporating data from clinical trials will be needed to elucidate how stage-specific and treatment-related factors interact with tumor cell total mRNA expression to determine patient outcome and to help select the most effective treatments for low- and high-TmS tumors.

Conceptually, analogous to DNA ploidy measuring the average number of haploid genomes in tumors, the average total mRNA content per haploid genome can be considered the ‘ploidy of the transcriptome’. Total mRNA content is a key parameter of tumor heterogeneity and phenotype plasticity, previously hidden in most RNA-based assays. Although our current work focuses on interpretation of mRNA, the methodology developed here can readily be applied to the quantification of other RNA species (for example, rRNA, miRNA and piRNA), further illuminating the cancer transcriptome. Enhanced attention to ‘transcriptome ploidy’ will enable better phenotypic characterization and a deeper biological understanding of transcriptional dysregulation in cancer and other diseases.

## Methods

Additional details and results are described in the Supplementary Notes. Here, we summarize the key aspects of the analysis.

### Total mRNA expression in scRNA-seq data

#### Dataset

We collected scRNA-seq data from ten patients, comprising three with colorectal adenocarcinoma, three with hepatocellular carcinoma, two with lung adenocarcinoma and two with pancreatic adenocarcinoma (Supplementary Table 1). A full description is provided in Supplementary Note 1.1. The three colorectal adenocarcinoma patient samples were obtained with informed consent and were approved by the Human Subjects Protection Office, the Clinical Research Committee as well as five separate institutional review boards at MD Anderson Cancer Center, in accordance with the Declaration of Helsinki.

#### Quality control, clustering, cell type annotation and normalized UMI

For each sample, we first filtered out cells based on number of genes expressed, total UMI counts and proportion of total UMI counts derived from mitochondrial genes. We also removed cells that were detected as doublets. After the quality control, 48,913 cells remained from the ten human tumor samples. Within each patient sample, highly variable genes were detected and used for principal component analysis (PCA). Cells were then clustered with the Seurat package^68^. Cell type was annotated using known marker genes^34,35,69–71^. Tumor cells were identified based on the inferred presence of somatic CNAs by inferCNV^72^. We further merged Seurat^68^-identified clusters that were not significantly different in gene counts, which is the total number of expressed genes (Wilcoxon rank-sum test, α = 0.001; Fig. 1b). A full description is provided in Supplementary Note 1.2.1.

To enable comparison among different scRNA-seq samples within the same study, we performed scale normalization to ensure that the total UMI count per cell was comparable across different samples from the same study. A full description is provided in Supplementary Note 1.2.2.

#### Trajectory and gene set enrichment analyses

We applied Monocle 2 (version 2.14.0)^44–46^ to construct single-cell trajectories and used the CytoTRACE (version 0.3.3) score to measure the differentiation state of tumor cells^16^. To compare CytoTRACE scores among the tumor cell clusters from patient samples within the same cancer type, we integrated tumor cells from patients 1, 2 and 3 from colorectal cancer and patients 1 and 2 from each of the lung and pancreatic cancers using ComBat (version 3.20.0)^73^ embedded in CytoTRACE, which corrects for batch effects. We quantified gene set enrichment for the high-UMI versus low-UMI tumor cell clusters using the GeneOverlap R package (version 1.24.0)^74^. A comprehensive set of signatures with 18,617 human gene sets (containing at least four genes) was compiled from the Molecular Signatures Database (version 6.2)^47^ and CellMarker^48^. A full description is provided in Supplementary Note 1.2.4.

#### Pseudo-bulk analysis

We pooled normalized scRNA-seq data to form pseudo-bulk samples and estimated the ratio of the mean total UMI counts of tumor cells to that of the non-tumor cells for each sample. The 95% CIs were constructed by bootstrapping the same numbers of tumor and non-tumor cells with 1,000 repetitions.

### Tumor-specific total mRNA expression in bulk sequencing data

#### A mathematical model for tumor-specific total mRNA expression estimation

For any group of cells, we use *S* to denote the average global mRNA transcript level per cell per haploid genome, which follows $S = \mathop {\sum}\nolimits_{c = 1}^C {\left( {\mathop {\sum}\nolimits_{g = 1}^G {u_{gc}/p_c} } \right)/C}$. Here, *u*_*gc*_ denotes the number of mRNA transcripts of gene *g* in cell *c*; *G* is the total number of genes; *C* is the number of cells; and *p*_*c*_ is the ploidy—that is, the number of copies of the haploid genome in cell *c*. However, the cell-level ploidy *p*_*c*_ is usually not measurable. Hence, in practice, we use average ploidy Ψ of the corresponding cell group to approximate it: $S \approx \mathop {\sum}\nolimits_{c = 1}^C {\mathop {\sum}\nolimits_{g = 1}^G {u_{gc}/(C\Psi )} }$. For non-tumor cells, which are commonly diploid, this assumption is assured.

In the analysis of bulk RNA sequencing data from mixed tumor samples, we are interested in comparing tumor to non-tumor cell groups. We let *T* denote tumor cells and *N* denote non-tumor cells. Therefore, we define a TmS to reflect the ratio of total mRNA transcript level per haploid genome of tumor cells to that of the surrounding non-tumor cells—that is, TmS_tumor_ = *S*_*T*_ / *S*_*N*_, simplified as TmS from here forward. It is necessary to calculate this ratio to cancel out technical effects presented in sequencing data that confound with both *S*_*T*_ and *S*_*N*_. Let $T_g = \mathop {\sum}\nolimits_{c = 1}^{C_T} {u_{gc}}$ and $N_g = \mathop {\sum}\nolimits_{c = 1}^{C_N} {u_{gc}}$ denote the total number of mRNA transcripts of gene *g* across all cells from tumor and non-tumor cells; let $T_ + = \mathop {\sum}\nolimits_{g = 1}^G {T_g} ,N_ + = \mathop {\sum}\nolimits_{g = 1}^G {N_g} ,$ *C*_*T*_ and *C*_*N*_ denote the total number of tumor and non-tumor cells; and let Ψ_*T*_ and Ψ_*N*_ represent the average ploidy of tumor and non-tumor cells, respectively. Under the assumption that the tumor cells have a similar ploidy, we can derive TmS without using single-cell-specific parameters as

1 $${\mathrm{TmS}} = [T_ + /(C_T\Psi _T)]/[N_ + /(C_N\Psi _N)] = [T_ + /N_ + ]/[(C_T\Psi _T)/(C_N\Psi _N)]$$

We further introduce the proportion of total bulk mRNA expression derived from tumor cells (hereafter ‘tumor-specific mRNA proportion’) $\pi = \left( {\mathop {\sum}\nolimits_{g = 1}^G {T_g} } \right)/\left( {\mathop {\sum}\nolimits_{g = 1}^G {T_g} + \mathop {\sum}\nolimits_{g = 1}^G {N_g} } \right)$ and the tumor cell proportion (hereafter ‘tumor purity’) *ρ* = *C*_*T*_ /(*C*_*T*_ + *C*_*N*_). We, thus, have

2 $$\begin{array}{*{20}{l}} {\mathrm{TmS}} \hfill & = \hfill & {\left[ {\pi /(1 - \pi )} \right]/\left[ {\left( {\rho /\left( {1 - \rho } \right)} \right)\left( {\mathop {\Psi }\nolimits_T /\mathop {\Psi }\nolimits_N } \right)} \right]} \hfill \\ {} \hfill & = \hfill & {\left[ {\pi \left( {1 - \rho } \right)\mathop {\Psi }\nolimits_N } \right]/\left[ {\rho \left( {1 - \pi } \right)\mathop {\Psi }\nolimits_T } \right]} \hfill \end{array}$$

The tumor-specific mRNA proportion *π* derived from the tumor can be estimated using DeMixT^31^ as $\hat \pi$; the tumor purity *ρ* and ploidy Ψ_*T*_ can be estimated using ASCAT^32^, ABSOLUTE^33^ or Sequenza^49^ based on the matched DNA sequencing data as $\hat \rho$ and $\widehat {{\Psi }}_T$, respectively; and the ploidy of non-tumor cells Ψ_*N*_ was assumed to be 2 (refs. ^32,33^). Hence, we have

3 $$\widehat {\mathrm{TmS}} = \frac{{\hat \pi (1 - \hat \rho )\Psi _N}}{{\hat \rho (1 - \hat \pi )\hat \Psi _T}}$$

In what follows, we use TmS to represent ${\widehat {\mathrm{TmS}}}$ for simplicity. A full description is provided in Supplementary Note 2.1.

#### Consensus of tumor purity and ploidy estimation

For DNA-based deconvolution methods such as ASCAT and ABSOLUTE, there could be multiple tumor purity *ρ* and ploidy Ψ_*T*_ pairs that have similar likelihoods. Both ASCAT and ABSOLUTE can accurately estimate the product of purity and ploidy *ρ*Ψ_*T*_; however, they sometimes lack power to identify *ρ* and Ψ_*T*_ separately. TmS is derived from the product of tumor ploidy and the odds of tumor purity. Hence, it is potentially more robust to ambiguity in the tumor purity and ploidy estimation, ensuring the robustness of the TmS calculation. We illustrate this robustness by showing that the agreement between TmS values calculated from ASCAT and ABSOLUTE are substantially improved, as compared to the agreement between the ploidy values calculated from the two methods that was low among 20% of TCGA samples (Extended Data Fig. 3f,g). To calculate one final set of TmS values for a maximum number of samples, we take a consensus strategy. We first calculate TmS values with tumor purity and ploidy estimates derived from both ABSOLUTE and ASCAT and then fit a linear regression model on the log_2_-transformed TmS_ASCAT_ by using the log_2_-transformed TmS_ABSOLUTE_ as a predictor variable. We remove samples with Cook’s distance ≥4 / *n* (*n* *=* 5,295; Extended Data Fig. 3h) and calculate the final ${\mathrm{TmS}} = \sqrt {{{\mathrm{TmS}}_{\mathrm{ASCAT}}} \times {{\mathrm{TmS}}_{\mathrm{ABSOLUTE}}}}$.

#### Improved estimation of tumor-specific mRNA proportion

The identifiability of model parameters is a major issue for high-dimensional models. With the DeMixT model, there is hierarchy in model identifiability in which the cell-type-specific mRNA proportions are the most identifiable parameters, requiring only a subset of genes with identifiable expression distributions. Therefore, our goal is to select an appropriate set of genes as input to DeMixT that optimizes the estimation of the tumor-specific mRNA proportions (*π*). In general, genes expressed at different numerical ranges can affect estimation of *π*. We found that including genes that are not differentially expressed between the tumor and non-tumor components, differentially expressed across tumor subtypes in different samples or with large variance in expression within the non-tumor component can introduce large biases to the estimated *π*. On the other hand, the tumor component is hidden in the mixed tumor samples, hence preventing a differential expression analysis between mixed and normal samples from finding the best genes. By applying a profile-likelihood-based approach to detect the identifiability of model parameters^75^, we systematically selected the top-ranking identifiable genes for the estimation of *π*. As a general method, the profile-likelihood-based gene selection strategy can be extended to any method that uses maximum likelihood estimation. We also employed a virtual ‘normal’ spike-in strategy to balance proportion distributions, which further improved the deconvolution performance. A full description is provided in Supplementary Note 2.2.

#### Profile-likelihood-based gene selection

In brief, in the DeMixT model, for sample $i \in (1,2, \ldots ,M)$ and gene $g \in (1,2, \ldots ,G)$, we have

4 $$Y_{ig} = \pi _iT_{ig}^\prime + \left( {1 - \pi _i} \right)N_{ig}^\prime$$

where *Y*_*ig*_ represents the scale-normalized expression count matrix observed from mixed tumor samples, and *T*′_*ig*_ and *N*′_*ig*_ represent the normalized relative expression of gene *g* within tumor and surrounding non-tumor cells, respectively. The estimated tumor-specific mRNA proportion $\hat \pi$ is the desirable quantity for Eq. 3. We assume each hidden component follows the log_2_-normal distribution—that is, $T_{ig}^\prime \sim LN\!\left( {\mu _{Tg},\sigma _{Tg}^2} \right)$ and $N_{ig}^\prime \sim LN\!\left( {\mu _{Ng},\sigma _{Ng}^2} \right)$. We will use notation *T* and *N* and drop the ′ sign from now on. The identifiability of a gene *k* in the DeMixT model is measured by the CI $[\mu _{Tk}^ - ,\mu _{Tk}^ + ]$ around the mean expression *μ*_*Tk*_. The definition of the profile likelihood function of *μ*_*Tk*_ is

5 $$\begin{array}{lll}l_{\mu _{Tk}}\!\left( {\mu _{Tk} = x|\pi ,\mu _T,\sigma _T} \right) \\= \mathop {{\max }}\limits_{\pi _i,\mu _{Tg},\sigma _{Tg},\sigma _{Tk}} \left\{ {\mathop {\sum}\limits_{i = 1}^M {\left[ {\mathop {\sum}\limits_{g \ne k}^G {\log \left( {f\left( {\pi _i,\mu _{Tg},\sigma _{Tg}} \right)} \right) + \log \left( {f\left( {\pi _i,\mu _{Tk} = x,\sigma _{Tk}} \right)} \right)} } \right]} } \right\}\end{array}$$

where

$$\begin{array}{lll}f\!\left( {Y_{ig}|\pi _i,\mu _{Tg},\sigma _{Tg}} \right) = \frac{1}{{2\pi \sigma _{Ng}\sigma _{Tg}}}\\ \times {\int}_0^{Y_{ig}} {\frac{1}{{t(Y_{ig} - t)}}} \exp \! \left( { - \frac{{\left( {\log 2\left( t \right) - \mu _{Ng} - \log 2\left( {1 - \pi _i} \right)} \right)^2}}{{2\sigma _{Ng}^2}} - \frac{{\left( {\log 2(Y_{ig} - t) - \mu _{Tg} - \log 2(\pi _i)} \right)^2}}{{2\sigma _{Tg}^2}}} \right)dt\end{array}$$

is the likelihood function of the DeMixT model.

The CI of a profile likelihood function can be constructed through inverting a likelihood-ratio test^76^. However, calculating the actual profile likelihood function of all genes (~20,000) is generally infeasible due to computational limits. We adopted an asymptotic approximation to quickly evaluate the profile likelihood function^75^, using the observed Fisher information of the log-likelihood, denoted as $H(\hat \pi ,\hat \mu _T,\hat \sigma _T)$. Then, the asymptotic *α-*level CI of *μ*_*Tk*_ can be written as^75^

6 $$\mu _{Tk}^ \pm = \widehat {\mu _{Tk}} \pm \sqrt {2\chi _{1 - \alpha }^2(1)H\left( {\hat \pi ,\hat \mu _T,\hat \sigma _T} \right)_{k,k}^{ - 1}}$$

We hereby introduce a gene selection score to represent the length of an asymptotic profile-likelihood-based 95% CI of *μ*_*Tk*_ for gene *k*,

7 $${\mathrm{gene}}\,{\mathrm{selection}}\,{\mathrm{score}}_k = 2\sqrt {2\chi _{0.05}^2(1)H\left( {\hat \pi ,\hat \mu _T,\hat \sigma _T} \right)_{k,k}^{ - 1}}$$

Genes with a lower score have a smaller CI, hence higher identifiability for their corresponding parameters in the DeMixT. Genes are ranked based on the gene selection scores from the smallest to the largest. A subset of genes that are ranked on top will be used for parameter estimation. In the DeMixT R package, our proposed profile-likelihood-based gene selection approach is included as function ‘DeMixT_GS’. A full description is provided in Supplementary Note 2.2.2. We performed a simulation study, mimicking the TCGA prostate adenocarcinoma dataset, to validate the proposed gene selection method. A full description is provided in Supplementary Note 2.2.3. The implementation of virtual ‘normal’ spike-ins and a simulation study is provided in Supplementary Note 2.2.4.

### TmS validation using bulk RNA sequencing data from mixed cell lines

We validated TmS estimates using an experimental dataset from a previous mixed cell line study (GSE121127)^31^ and selected a subset of 18 mixed samples with negligible RNA content from the immune component. Lung adenocarcinoma in humans (H1092) and CAF cells were mixed at different cell count proportions (Supplementary Table 3) to generate each bulk sample, plus three additional samples of 100% H1092 or 100% CAF. The raw reads were generated from paired-end total RNA Illumina sequencing and mapped to the human reference genome build 37.2 from the National Center of Biotechnology Information through TopHat^77^. SAMtools^78^ was applied to remove improperly mapped and duplicated reads. Picard tools were used to sort the cleaned SAM files according to their reference sequence names and create an index for the reads. The gene-level expression was quantified using the R packages GenomicFeatures and GenomicRanges.

For each cell line, we measured total RNA amount (in ng µl^−1^) for 1 million cells in three repeats using the Qubit RNA Broad Range Assay Kit (Life Technologies). The true TmS values of H1092 or CAF were then derived as a ratio of the total RNA amount per cell between the two cell types—specifically, ${\mathrm{TmS}}_{{\mathrm{H}}1092} = \frac{{{{{\mathrm{total}}}}\,{{{\mathrm{RNA}}}}\,{{{\mathrm{amount}}}}\,{{{\mathrm{per}}}}\,{{{\mathrm{cell}}}}\,{{{\mathrm{of}}}}\,{{{\mathrm{H}}}}1092}}{{{{{\mathrm{total}}}}\,{{{\mathrm{RNA}}}}\,{{{\mathrm{amount}}}}\,{{{\mathrm{per}}}}\,{{{\mathrm{cell}}}}\,{{{\mathrm{of}}}}\,{{{\mathrm{CAF}}}}}} = 0.87$ and ${\mathrm{TmS}}_{\mathrm{CAF}} = \frac{{{{{\mathrm{total}}}}\,{{{\mathrm{RNA}}}}\,{{{\mathrm{amount}}}}\,{{{\mathrm{per}}}}\,{{{\mathrm{cell}}}}\,{{{\mathrm{of}}}}\,{{{\mathrm{CAF}}}}}}{{{{{\mathrm{total}}}}\,{{{\mathrm{RNA}}}}\,{{{\mathrm{amount}}}}\,{{{\mathrm{per}}}}\,{{{\mathrm{cell}}}}\,{{{\mathrm{of}}}}\,{{{\mathrm{H}}}}1092}} = 1.2$. We estimated the RNA proportion of H1092 and CAFs using DeMixT (DeMixT_GS function with 4,000 genes selected) under two scenarios: (1) three pure CAFs samples were used as reference; and (2) three pure H1092 samples were used as reference. To estimate TmS values, we used the known cell counts to calculate *ρ* values.

### TmS estimation in patient cohorts

A full description of all datasets is provided in Supplementary Note 2.3.1.

#### TCGA datasets

Raw read counts of high-throughput mRNA sequencing data, clinical data and somatic mutations from 7,054 tumor samples across 15 TCGA cancer types (breast carcinoma, bladder urothelial carcinoma, colorectal cancer (colon adenocarcinoma + rectum adenocarcinoma), head and neck squamous cell carcinoma, kidney chromophobe, kidney renal clear cell carcinoma, kidney renal papillary cell carcinoma, liver hepatocellular carcinoma, lung adenocarcinoma, lung squamous cell carcinoma, pancreatic adenocarcinoma, prostate adenocarcinoma, stomach adenocarcinoma, thyroid carcinoma and uterine corpus endometrial carcinoma) were downloaded from the Genomic Data Commons Data Portal (https://portal.gdc.cancer.gov/). ATAC-seq data^52^, tumor purity and ploidy data^79,80^ and annotations of driver mutation and indels^81^ were downloaded for these samples.

#### Estimation of tumor-specific mRNA proportions from RNA sequencing data

For each cancer type, we filtered out poor-quality tumor and normal samples that were likely misclassified. We then selected available adjacent normal samples as reference for the tumor deconvolution using DeMixT. Based on simulation studies (Supplementary Note 2.2.3) and observed distributions of gene selection scores in real data, we chose the top 1,500 or 2,500 genes (varies across cancer types) to estimate tumor-specific mRNA proportions (*π*). For each cancer type, the selected 1,500 or 2,500 genes are defined as intrinsic tumor signature genes. We added varying numbers of virtual spike-in samples depending on cancer types. We additionally removed samples with extreme estimates of *π*, >85% or ranked at the top 2.5 percentile of all samples within each cancer type to mitigate the remaining underestimation when *π* is close to 1. A full description is provided in Supplementary Note 2.3.2.1.

#### Consensus TmS estimation

We calculated a consensus TmS as ${\mathrm{TmS}} = \sqrt {{\mathrm{TmS}}_{\mathrm{ASCAT}} \times {\mathrm{TmS}}_{\mathrm{ABSOLUTE}}}$ and removed 264 of 5,295 TCGA samples that deviated from our consensus model, as described previously. A full description on sample exclusions is provided in Supplementary Note 2.3.2.2.

#### Intrinsic tumor signature genes

For each cancer type, the selected genes used for estimating *π* are called intrinsic tumor signature genes. We conducted gene set enrichment analyses (GSEAs) on hallmark pathways and KEGG pathways^47^ for these genes ranked with their gene selection scores from small to large using GSEA^82^ and g:Profiler^83^. We further evaluated the chromatin accessibility of intrinsic tumor signature genes using ATAC-seq data from TCGA samples^52^. For each sample, we calculated the mean of the peak scores of selected genes and compared it with the corresponding permuted null distribution for each cancer type. A full description is provided in Supplementary Note 2.3.2.3.

#### Association of TmS with genetic alterations and metabolism

We searched among driver mutations (including nonsense, missense and splice-site single-nucleotide variants (SNVs) and indels)^81^ as well as all non-synonymous mutations (including SNVs and indels) over all genes for the 15 cancer types to identify those that were significantly associated with TmS. We investigated 24 cancer–gene pairs for the driver mutation analysis and 32,894 cancer–gene pairs for the non-synonymous mutation analysis. We applied a Wilcoxon rank-sum test to each candidate gene to compare the distributions of TmS of the samples with mutations versus without mutations. We also fitted a linear regression model on TmS to adjust for TMB. The *P* values of each gene were adjusted for multiple testing using Benjamini–Hochberg correction across all candidate genes within the corresponding cancer type. See Supplementary Note 2.3.2.4 for further details.

TMB was calculated by counting the total number of somatic mutations based on the consensus mutation calls (MC3)^84^. Chromosomal instability (CIN) scores were calculated as the ploidy-adjusted percent of genome with an aberrant copy number state. ASCAT was used to calculate allele-specific copy numbers^32^. For samples present in both TCGA and Pan-Cancer Analysis of Whole Genomes (PCAWG), the consensus copy number was derived from published results^85^. Tumor samples that had undergone whole-genome duplication (WGD) were identified based on homologous copy number information^33^.

For each cancer type from TCGA, we conducted GSEAs^82^ on the metabolism of carbohydrate pathways (the Reactome database^86^). The genes were ranked by the Spearman correlation coefficient between their expression levels and TmS across samples; they were then put through GSEA in the ‘pre-ranked’ mode. For GSEA, we adopted permutation tests (1,000 times) to generate a normalized enrichment score (NES) for each candidate pathway. A hierarchical clustering on the expression levels of the Reactome pentose phosphate pathway (15 genes total, of which two genes were removed due to high-frequency zero counts across samples) for the tumor samples was performed using Euclidean distance and Ward linkage. The samples were then separated into two groups using the ‘cutree’ function. For each cancer type, a Wilcoxon rank-sum test was used to compare the distributions of TmS estimates between the two tumor sample groups. *P* values were adjusted for multiple testing using Benjamini–Hochberg correction across all cancer types.

#### ICGC-EOPC dataset

In this cohort, matched mRNA sequencing data and whole-genome sequencing data, as well as clinical data including biochemical recurrence, Gleason score and pathologic stage, from 121 tumor samples and nine adjacent normal samples from 96 patients (age at treatment <55 years) were downloaded from Gerhauser et al.^37^ We used the nine available adjacent normal samples as the normal reference. The mRNA sequencing data came from three batches: batch 1 (17 patients and 25 samples), batch 2 (42 patients and 52 samples) and batch 3 (37 patients and 44 samples). We observed consistency and robustness of DeMixT results with or without batch effect correction. See Supplementary Notes 2.3.1 and 2.3.3 for further details.

#### METABRIC dataset

This dataset included 1,992 pairs of expression arrays and Affymetrix SNP 6.0 arrays profiled for tumor samples from 1,992 patients, which was divided into a discovery set (997 patients) and a validation set (995 patients)^38^. A total of 144 expression arrays for adjacent normal tissues were provided.

We applied the DeMixT deconvolution pipeline to the expression arrays of the combined discovery and validation sets, after batch effect correction, to estimate tumor-specific proportions using the adjacent normal samples as the reference. Affymetrix CEL files were processed by PennCNV^87^ to obtain the LogR and B allele frequency (BAF) data, followed by both ASCAT^32^ and Sequenza^49^ to estimate tumor purity and ploidy for each sample. The consensus TmS strategy was applied to obtain robust TmS estimations. In total, 1,664 patient samples with TmS remained after the above steps. We additionally removed 118 patient samples due to missing follow-up information of biochemical recurrence intervals or the PAM50 subtypes. A final cohort of 1,546 patient samples from both the discovery and validation sets was kept for downstream analyses. See Supplementary Notes 2.3.1 and 2.3.4 for further details.

#### TRACERx dataset

A total of 159 tumor samples from 64 patients with matched RNA sequencing data and WES data were downloaded^39,40,88^ (see Supplementary Note 2.3.1 for further details). Tumor purity and ploidy were estimated from WES data by Sequenza^49^. We used RNA sequencing data from normal lung samples without significant pathology in the corresponding tissue types in the GTEx study as the reference for the deconvolution of tumor samples in this dataset (see Supplementary Note 2.3.5 for further details). Focusing on tumor samples with tumor purity > 0.15, we calculated TmS for 116 regions from 52 patient samples, among which 30 patients have at least two regions. We further performed association analysis of regional and sample-specific TmS with measures of chromosomal instability. We defined the subclonal CNA as a CNA presented only in a subset of regions. We further define the evolutionary relationship in two regions from the same patient as either linear or branched. For each evolutionary relationship per patient, we defined the ‘range of TmS’ as *log*_*2*_*(TmS*_*max*_*) − log*_*2*_*(TmS*_*min*_*)* across regions. We fitted linear regression models by taking *log*_*2*_*(TmS*_*max*_*)* as the response variable and the percentage of subclonal CNA, number of regions, range of TmS, evolutionary relationship and their interactions as predictors. The best model was selected by stepwise selection based on the Bayesian information criterion (BIC)^89^. See Supplementary Note 3.3 for further details.

### Statistical analysis

#### Batch effect correction

For RNA sequencing data from multiple batches, we applied batch effect correction using ComBat^73^ and limma^90^ to combine RNA sequencing data in one pool before estimating tumor-specific mRNA proportions. See Supplementary Note 3.1 for further details on the robustness of TmS estimation.

#### Association with clinical variables

Kruskal–Wallis tests were used to compare the distribution of TmS between subgroups defined by each clinical variable. The *P* values from the Kruskal–Wallis tests were adjusted using Benjamini–Hochberg correction across all available clinical variables within the corresponding cancer type.

#### Association with survival outcomes

Associations with TmS were assessed in terms of OS, PFI and DFS depending on cancer type and study cohort. For TCGA, we used outcome measures that are recommended by Liu et al.^61^. If both OS and PFI were recommended, we used the more clinically relevant outcomes for an individual cancer type. We dichotomized pathologic stages into two categories: early (I/II) and advanced (III/IV). For prostate cancers, we used the Gleason score (Gleason score = 7 versus 8+) instead of early and advanced stages. Furthermore, we followed clinical guidelines and physician recommendations to identify tumor samples that were treated without systemic therapy (surgery only) in TCGA and used the corresponding meaningful outcome measures for the selected populations. For all association analyses with clinical outcomes across datasets, we used a recursive partitioning survival tree model, rpart^91^, to find the optimal TmS cutoff (high versus low) separating different survival outcomes within each of the two stages defined above in each cancer type. Splits were assessed using the Gini index, and the maximum tree depth was set to 2. Log-rank tests between high- and low-TmS groups within early or advanced pathologic stages were performed. We performed sensitivity analysis on the TmS cutoff to confirm that a similar trend can be observed with other values. See Supplementary Note 3.2 for further details on the survival analysis and the identification of patients without systemic therapy.

#### Cox regression with model selection

We fitted multivariate Cox proportional hazard models with age, stage, TmS (high versus low) and other variables as predictors of OS, PFI or DFS for each dataset and calculated HRs and 95% CIs. We use the stepwise model selection method with BIC^89^, where the baseline model includes age, stage and TmS predictors, and additional variables to select include the interaction term of TmS × stage.

### Reporting summary

Further information on research design is available in the Nature Research Reporting Summary linked to this article.

## Online content

Any methods, additional references, Nature Research reporting summaries, source data, extended data, supplementary information, acknowledgements, peer review information; details of author contributions and competing interests; and statements of data and code availability are available at 10.1038/s41587-022-01342-x.

## Supplementary information

Supplementary Information Supplementary Notes, Supplementary Note Figs. 1–27 and Supplementary Note Tables 1–9

Reporting Summary

Supplementary Table 1 Clinical information of the ten patients in the scRNA-seq data analysis

Supplementary Table 2 Single-cell GSEAs for high-UMI and low-UMI tumor cells in each of the ‘patient 1’ samples with colorectal, lung and pancreatic cancers

Supplementary Table 3 Benchmarking results using a mixed cell line experiment

Supplementary Table 4 Summary of the distributions of TmS across 15 cancer types in TCGA, ICGC-EOPC, METABRIC and TRACERx

Supplementary Table 5 Multivariate Cox proportional hazard models with age, TmS, stage and TmS × stage as candidate predictors for OS and PFI analysis across cancer types in TCGA and ICGC-EOPC

Supplementary Table 6 Summary of patients without systemic therapy across cancers

Supplementary Table 7 Multivariate Cox proportional hazard models with age, TmS, chemotherapy and Oncotype risk as predictors for DFS analysis across breast cancer subtypes in the METABRIC study

## Data Availability

The UMI counts of the hepatocellular carcinoma scRNA-seq data were downloaded from ﻿the Gene Expression Omnibus under accession number GSE125449. The UMI counts and cell type annotations of the lung adenocarcinoma scRNA-seq data were downloaded from the ﻿ArrayExpress under accession number E-MTAB-6149. The UMI counts of the colorectal adenocarcinoma scRNA-seq data are available at http://crcmoonshot.org/?page_id=189. FASTQ files of scRNA-seq data from pancreatic cancer is publicly available on the Gene Expression Omnibus under accession number GSE156405. Raw read counts from the mixed cell line study were downloaded from the Gene Expression Omnibus under accession number GSE121127. Raw read counts of RNA sequencing data, clinical data and somatic mutations from 7,054 tumor samples across 15 TCGA cancer types are available for download from the Genomic Data Commons Data Portal (https://portal.gdc.cancer.gov/). ATAC-seq data for TCGA samples were downloaded from https://science.sciencemag.org/content/362/6413/eaav1898/tab-figures-data. Clinical information of ICGC-EOPC was downloaded from https://www.sciencedirect.com/science/article/pii/S1535610818304823?via%3Dihub#gs1. All primary METABRIC data, including Affymetrix SNP 6.0 CEL files and Illumina HT-12 gene expression arrays, are available at the European Genome-phenome Archive (EGAS00000000083) and may be downloaded from https://ega-archive.org/studies/EGAS00000000083. Clinical information of METABRIC was downloaded from https://www.cbioportal.org/study/clinicalData?id=brca_metabric. Clinical information of TRACERx was downloaded from https://www.nejm.org/doi/full/10.1056/NEJMoa1616288#article_supplementary_material. WES data of TRACERx were downloaded from https://ega-archive.org/studies/EGAS00001002247. RNA sequencing data of TRACERx were downloaded from https://ega-archive.org/studies/EGAS00001003458. TmS values of all samples and the identified intrinsic tumor signature genes for this study are available for download at https://github.com/wwylab/TmS. All other relevant data are available from the corresponding author upon reasonable request. Source data are provided with this paper.

## Source data

Source Data Fig. 1 Statistical Source Data
Source Data Fig. 2 Statistical Source Data
Source Data Fig. 3 Statistical Source Data
Source Data Fig. 4 Statistical Source Data
Source Data Fig. 5 Statistical Source Data
Source Data Extended Data Fig. 1 Statistical Source Data
Source Data Extended Data Fig. 2 Statistical Source Data
Source Data Extended Data Fig. 3 Statistical Source Data
Source Data Extended Data Fig. 4 Statistical Source Data
Source Data Extended Data Fig. 5 Statistical Source Data
Source Data Extended Data Fig. 6 Statistical Source Data
Source Data Extended Data Fig. 7 Statistical Source Data
Source Data Extended Data Fig. 8 Statistical Source Data
Source Data Extended Data Fig. 9 Statistical Source Data
Source Data Extended Data Fig. 10 Statistical Source Data

## Code availability

DeMixT used for estimating tumor-specific mRNA expression proportion is freely available as an R package and can be downloaded from https://github.com/wwylab/DeMixT. DeMixT version 1.2.2 was used to generate the results in this work. A tutorial for estimating TmS based on the DeMixT output is available at https://github.com/wwylab/TmS.
