# Supplementary material for: Estimation of tumor cell total mRNA expression in 15 cancer types predicts disease progression
Source: Nat Biotechnol. 2022 Jun 13;40(11):1624–33. doi: 10.1038/s41587-022-01342-x (PMC9646498; doi:10.1038/s41587-022-01342-x)
Supplement: Supplementary file 1 — Supplementary Notes, Supplementary Note Figs. 1–27 and Supplementary Note Tables 1–9 [file 41587_2022_1342_MOESM1_ESM.pdf]

nature portfolio
https://doi.org/10.1038/s41587-022-01342-x

Supplementary information

Estimation of tumor cell total mRNA expression in 15 cancer types predicts disease progression

In the format provided by the authors and unedited

Supplementary Note

Table of Contents

1. Total mRNA expression in single-cell RNA sequencing data 2

1.1. Datasets 2

1.2. Single-cell RNA sequencing data processing 2

1.2.1. Quality control, clustering and cell type annotation 3

1.2.2. Normalized total UMI counts 6

1.2.3 Cell cycle states of tumor cells 11

2. Tumor-specific total mRNA expression in bulk sequencing data 12

2.1. A mathematical model for tumor-specific total mRNA expression 12

2.1.1. Model 13

2.1.2. Estimation 13

2.2. Improved estimation using DeMixT 16

2.2.1. Likelihood model for DeMixT 16

2.2.2. Optimized model identifiability 17

2.2.3. A simulation study for profile-likelihood based gene selection 19

2.2.4. Virtual spike-ins to improve identifiability and a simulation study 22

2.3. Tumor-specific total mRNA expression in patient samples 22

2.3.1. Datasets 23

2.3.2. TCGA 25

2.3.2.1. Data processing 25

2.3.2.2. Consensus TmS estimation 28

2.3.2.3. Intrinsic tumor signature genes 29

2.3.2.4 Association of TmS with genetic alterations 31

2.3.2.5 Association of TmS with expressions of pluripotency and proliferation genes 32

2.3.3. ICGC-EOPC 35

2.3.4. METABRIC 35

2.3.5. TRACERx 36

3. Statistical analysis 39

3.1. Robustness of TmS 39

3.1.1. Batch effect correction 39

3.1.2. Adjustment for focal copy number alterations 41

3.2. Survival analysis 43

3.2.1. Association analysis of TmS in survival outcomes 43

3.2.2. Identification of patients treated without systemic therapy in TCGA 48

3.3. Regional TmS analysis in TRACERx 48

1. TOTAL MRNA EXPRESSION IN SINGLE-CELL RNA SEQUENCING DATA

1.1. Datasets

Colorectal cancer single-cell RNA sequencing data

Three fresh colorectal adenocarcinoma samples of primary tumor were collected from patients who were receiving chemotherapies by surgical resection at the University of Texas MD Anderson Cancer Center (**Supplementary Table 1**). Single-cell data was generated using the Chromium Single Cell 3' Library, Gel Bead & Multiplex Kit, and Chip Kit (v3, 10x Genomics). Libraries were sequenced on an Illumina NovaSeq6000. Alignment, tagging, and gene and transcript counting were conducted using the 10x Genomic Cell Ranger pipeline (version 3.0).

Liver cancer single-cell RNA sequencing data[1]

Three fresh hepatocellular carcinoma samples of primary tumor were collected at the NIH Clinical Center for immune checkpoint inhibition studies (NCT01313442) (**Supplementary Table 1**). Two of them (patient 1 and patient 2) were from patients who were receiving immunotherapies by needle biopsy, and the other was collected from an untreated patient by surgical resection. Single-cell data was generated using the Chromium Single Cell 3' Library, Gel Bead & Multiplex Kit, and Chip Kit (v2, 10x Genomics). Libraries were sequenced on an Illumina NextSeq500. Alignment, tagging, and gene and transcript counting were conducted using the 10x Genomic Cell Ranger pipeline (version 2.0.2).

Lung cancer single-cell RNA sequencing data[2]

Two fresh lung adenocarcinoma samples of primary, non-metastatic lung tumor were collected from untreated patients by surgical resection at University Hospital Leuven (**Supplementary Table 1**). Single-cell data was generated using the Chromium Single Cell 3' Library, Gel Bead & Multiplex Kit, and Chip Kit (v1, 10x Genomics). Libraries were sequenced on Illumina HiSeq4000. Alignment, tagging, and gene and transcript counting were conducted using the 10x Genomic Cell Ranger pipeline (version 2.0.0).

Pancreatic cancer single-cell RNA sequencing data[3]

Two untreated patients with primary pancreatic cancer were recruited at the University of Texas MD Anderson Cancer Center and informed written consents following institutional review board approval were obtained (Lab00-396 and PA15-0014). Fresh biopsies were collected from the tumors by fine needle aspiration (**Supplementary Table 1**). Single-cell data was generated using the Chromium Single Cell 3' Library, Gel Bead & Multiplex Kit, and Chip Kit (v1, 10x Genomics). Libraries were sequenced on an Illumina NextSeq500. Alignment, tagging, and gene and transcript counting were conducted by using the 10x Genomic Cell Ranger pipeline (version 3.1).

1.2. Single-cell RNA sequencing data processing

In this section, we first introduce the preprocessing for the single-cell RNA sequencing (scRNA-seq) datasets described above (including quality control, cell clustering, cell type annotation), followed by the introduction of a method to group cell clusters within a cell type based on gene counts, *i.e.*, the total number of expressed genes, to simplify the characterization of heterogeneity within the cell type. We then introduce a scale normalization method to correct for sequencing or experimental biases on total UMI counts, and finally the trajectory and cell cycle analyses for the scRNA-seq data.

Supplementary Note Table 1. Marker genes used to annotate cell types in scRNA-seq patient samples from four cancer types.

	Colorectal adenocarcinoma[5]	Hepatocellular carcinoma[1]	Lung adenocarcinoma[2]	Pancreatic adenocarcinoma[3,7]
B cell	*CD79A*, *CD38*	*CD79A*, *SLAMF7*, *BLNK*	*CD79A*, *IGKC*, *IGLC3*	*CD79A*, *CD38*
T cell	*CD2*, *CD3E*, *CD3D*	*CD2*, *CD3E*, *CD3D*	*CD3D*, *TRBC1*, *TRBC2*	*CD2*, *CD3D*
NK cell				*NKG7*, *KLRF1*
Myeloid	*CD14*, *CD68*, *ITGAX*	*CD14*, *CD163*, *CD68*	*LYZ*, *MARCO*, *CD68*	*CD14*, *CD68*
Fibroblast	*COLA1A*, *COL1A2*, *COL3A1*	*COL1A2*, *FAP*, *PDPN*	*COLA1A*, *DCN*, *COL1A2*	*COLA1A*, *COL1A2*
Endothelial	*PECAM1*, *VWF*, *ENG*	*PECAM1*, *VWF*, *ENG*	*CLDN5*, *FLT1*, *CDH5*	
Alveolar			*FOLR1*, *AQP4*, *PEBP4*	
Epithelial	*EPCAM*, *KRT18*, *KRT20*		*CAPS*, *TEME190*, *PIFO*, *SNTN*	*EPCAM*, *KRT18*, *KRT20*
Tumor cell			*LCN2*, *CCL20*, *PTTG1*	

1.2.1. Quality control, clustering and cell type annotation

For each of the three colorectal adenocarcinoma scRNA-seq samples generated at MD Anderson, genes expressed in less than three cells were removed. Cells with either fewer than 500 total UMIs, below 200 expressed genes, or more than 50% total UMI counts derived from mitochondrial genes were excluded. The total number of transcripts in each cell was normalized to 10,000, which was followed by a natural log transformation. Highly variable genes were detected and used for principal component analysis (PCA). Cells were then clustered with the Seurat package[4]. The cell type for each cell was annotated based on known marker genes[5] (**Supplementary Note Figure 1**, **Supplementary Note Table 1**). Initial somatic copy number variation (CNV) estimates were made using inferCNV[6], which was used to calculate CNV scores and CNV correlation scores[1]. The CNV score of a single cell was defined as the sum of the squared copy number variants across all gene positions. The CNV correlation score was calculated as the correlation between the copy number variations of a single cell and the average copy number variation of the top 2% cells ranked by CNV scores from the same sample. Tumor cells were identified as epithelial cells with an average CNV score greater than 0.0015. The three samples from patient 1, patient 2 and patient 3 had 5,444, 7,462 and 2,445 cells remaining, respectively, after data pre-processing.

The quality control of the three hepatocellular carcinoma scRNA-seq patient samples was conducted following the method described by Ma, L. *et al*[1]. For each sample, genes expressed in less than 0.1% of

the cells were removed. Cells with fewer than 700 total UMIs, fewer than 500 expressed genes, or more than 20% total UMI counts derived from mitochondrial genes were excluded. An additional quality control step of doublet removal was performed based on the number of cells loaded and recovered. The total number of transcripts in each cell was normalized to 10,000, followed by a natural log transformation. Highly variable genes were detected and used for PCA. Cells were then clustered with the Seurat package[4]. The cell type for each cell was annotated based on known marker genes[1] (**Supplementary Note Figure 1**, **Supplementary Note Table 1**). Tumor cells were identified as epithelial cells with CNV scores above the 80th percentile and CNV correlation scores above 0.4. The three samples of patient 1, patient 2 and patient 3 had 83, 761 and 796 cells remaining, respectively, after data pre-processing.

The quality control of the two lung adenocarcinoma scRNA-seq patient samples was conducted following the method described by Lambrechts, D. *et al*[2]. For each sample, genes expressed in less than 0.5% of the cells were removed. Any cell with either fewer than 201 total UMI counts, below 101 or over 6,000 expressed genes, or more than 10% total UMI counts derived from mitochondrial genes were filtered out from downstream analysis. The total number of transcripts in each cell was normalized to 10,000, followed by a natural log transformation. Highly variable genes were detected and used for PCA. Cells were then clustered with the Seurat package[4]. Cell type (including tumor cell) of each cell was annotated based on known marker genes[2] (**Supplementary Note Figure 1**, **Supplementary Note Table 1**). The two samples (patient 1 and patient 2) had 8,845 and 13,658 cells remaining, respectively, after data pre-processing.

For each of the two pancreatic adenocarcinoma scRNA-seq samples, genes expressed in less than three cells were removed. Cells with either fewer than 500 total UMIs, below 200 expressed genes, or more than 50% total UMI counts derived from mitochondrial genes were filtered out. The total number of transcripts in each cell was normalized to 10,000, followed by a natural log transformation. Highly variable genes were detected and used for PCA. Cells were then clustered with the Seurat package[4]. Cell type of each cell was annotated based on known marker genes[7] (**Supplementary Note Figure 1**, **Supplementary Note Table 1**). Tumor cells were identified as epithelial cells with CNV score above 0.015 and CNV correlation above 0.4. The two samples (patient 1 and patient 2) had 2,404 and 7,037 cells remaining after QC, respectively, after data pre-processing.

Within each cell type, we further merged clusters that did not significantly differ in gene counts (two-sided Wilcoxon rank-sum test, α=0.001, **Supplementary Note Figure 2**).

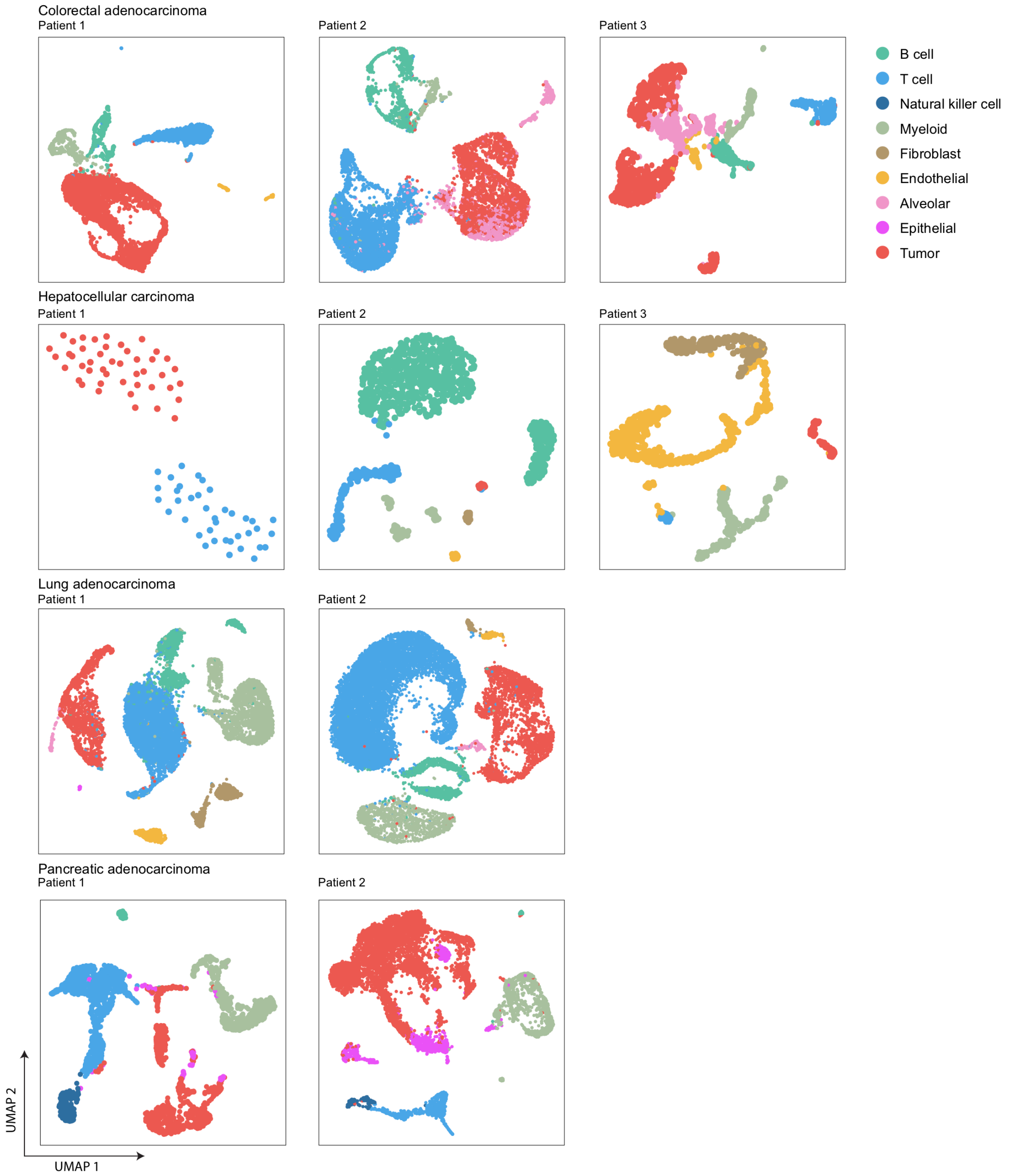

Supplementary Note Figure 1. UMAPs of scRNA-seq data from four cancer types.

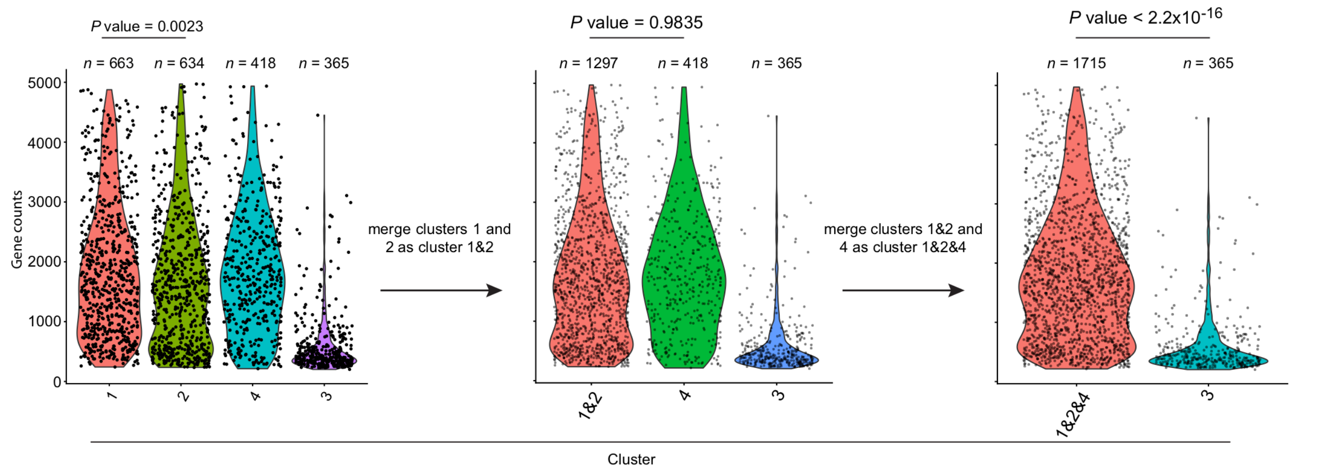

Supplementary Note Figure 2. **An example of merging cell clusters by gene counts.** Tumor cells in patient 2 of colorectal adenocarcinoma are used. The initial 4 clusters were determined by Seurat clustering (resolution=0.5). Two-sided Wilcoxon rank-sum tests comparing gene counts were performed between clusters and those that did not pass the significance level of 0.001 were merged. The resulting two tumor cell clusters had *n*=1,715 cells (high UMI cluster, e.g. 1, 2, and 4) and *n*=365 cells (low UMI cluster, e.g., 3), respectively. We repeated this process based on the initial Seurat clustering with resolution=1.0. There were still two tumor cell clusters after merging. The differences of tumor cells in the high UMI cluster and in the low UMI cluster based on the two resolutions were only *n*=12 cells and *n*=13 cells, respectively.

1.2.2. Normalized total UMI counts

We performed scale normalization on the raw count data to ensure the total UMI count per cell across all cells are comparable for different samples. Specifically, let $UMI_i = \{UMI_{igc}\}_{GxC_i}$ be a matrix of raw UMI counts for the scRNA-seq data for sample *i* being investigated, with genes *g* on the rows and cells *c* on the columns. *G* denotes the total number of genes, C_i is the number of cells in sample *i*. Then, the normalized UMI matrix UMI_i, denoted as UMI_i^{norm}, is calculated as $UMI_i^{norm}=UMI_i/r_i$, where, $r_i = \frac{UMI_i^{sum}/C_i}{baseline}$, $baseline = median\{UMI_1^{sum}/C_1, UMI_2^{sum}/C_2, \ldots, UMI_n^{sum}/C_n\}$, $UMI_i^{sum} = \sum_{c=1}^{C_i}\sum_{g=1}^{G} UMI_{igc}$.

Given a cell cluster**,** we let u_{gc} denote the amount of mRNA of gene *g* in cell *c*. The average total mRNA amount per cell is $\sum_{c=1}^{C}(\sum_{g=1}^{G} u_{gc})/C$. For scRNA-seq data, we assume the UMI_{gc} from gene *g*, cell *c* is proportional to the total mRNA u_{gc} of gene *g* in that cell, with a constant k_g that represents technical effects: $UMI_{gc}=k_g * u_{gc}$. The constant k_g is introduced because every single-cell sequencing platform presents a <100% capture efficiency for mRNA, and such efficiency varies across different platforms[9]. Under the assumption that the technical effect k_g remains constant across cells and is often evaluated as an average effect across genes within the same platform, we can evaluate total mRNA expression in the scRNA-seq data using the average total UMI counts, which is $\sum_{c=1}^{C}(\sum_{g=1}^{G} UMI_{gc})/C$. Notably, we observed

strong correlations between gene counts and total UMI across cells in each cell cluster across all cell types and cancer types (**Supplementary Note Figure 3**). This observation supports our assumption of a stable technical effect k_g within each study, and that the average total UMI counts serve as a reasonable surrogate to compare total mRNA expression across cells that are generated from the same experiment.

The average gene counts and average total UMI counts for both individual cell clusters and all the clusters pooled within a cell type are summarized in **Supplementary Note Table 2**.

The observed fold changes in total UMI counts between tumor cell clusters were significantly higher than those expected from expression dosage response from genome ploidy changes alone (at 2-3 folds[10,11]) among tumor cells (**Supplementary Note Table 3**). For the two tumor cell clusters in each patient across four cancer types, our null hypothesis is that there is no difference between the distribution of the total UMI counts from the tumor cell high-UMI cluster and the distribution of the total UMI counts from the tumor cell low-UMI cluster multiplied by three. For each patient, the *P* value was obtained with a t-test and adjusted by the Benjamini-Hochberg (BH) method[12]; the 95% confidence intervals were calculated using bootstrapping with 1,000 iterations.

Supplementary Note Table 2. The average gene counts and average total UMI counts for both individual cell clusters and all the clusters pooled. The 95% CI was estimated using bootstrapping with 1,000 iterations.

Cancer type	Patient id	Cell cluster	Cell type									
			Tumor		Epithelial		Alveolar		Endothelial		Fibroblast	
			Average gene counts (95% CI)	Average total UMI counts (95% CI)	Average gene counts (95% CI)	Average total UMI counts (95% CI)	Average gene counts (95% CI)	Average total UMI counts (95% CI)	Average gene counts (95% CI)	Average total UMI counts (95% CI)	Average gene counts (95% CI)	Average total UMI counts (95% CI)
Colorectal adenocarcinoma	Patient 1 (Stage IVA, DFS = 4 months)	Cluster 3	5,649 (5,526, 5,779)	48,706 (46,811, 50,691)	NA	NA	NA	NA	NA	NA	NA	NA
		Cluster 2	2,438 (2,325, 2,561)	13,787 (12,661, 15,063)	NA	NA	NA	NA	NA	NA	NA	NA
		Cluster 1	646 (620, 675)	1,929 (1,805, 2,058)	NA	NA	NA	NA	NA	NA	NA	NA
		Pooled	1,926 (1,785, 2,074)	13,626 (11,990, 15,228)	NA	NA	NA	NA	2,135 (2,041, 2,239)	7,378 (6,845, 7,899)	NA	NA
	Patient 2 (Stage IVA, DFS ≥ 22 months)	Cluster 2	1,782 (1,710, 1,848)	7,307 (6,910, 7,700)	NA	NA	NA	NA	NA	NA	NA	NA
		Cluster 1	604 (573, 638)	1,964 (1,800, 2,142)	NA	NA	NA	NA	NA	NA	NA	NA
		Pooled	1,576 (1,506, 1,642)	6373 (5988, 6781)	1,272 (1,225, 1,326)	4,455 (4,199, 4,721)	NA	NA	NA	NA	NA	NA
	Patient 3 (Stage IVA, DFS = 5 months)	Cluster 2	2,286 (2205, 2368)	15877 (15046, 16786)	NA	NA	NA	NA	NA	NA	NA	NA
		Cluster 1	1042 (1008, 1077)	4986 (4713, 5258)	NA	NA	NA	NA	NA	NA	NA	NA
		Pooled	1,796 (1,725, 1,873)	11,589 (10,874, 12,351)	730 (686, 776)	3097 (2774, 3485)	NA	NA	960 (897, 1031)	3997 (3609, 4369)	NA	NA
Hepatocellular carcinoma	Patient 1 (Stage IV, PFS < 5 months)	Cluster 2	5,338 (5,252, 5,425)	48,457 (47,098, 49,861)	NA	NA	NA	NA	NA	NA	NA	NA
		Cluster 1	1,364 (1,325, 1,405)	4,670 (4,402, 4,954)	NA	NA	NA	NA	NA	NA	NA	NA
		Pooled	3,660 (3,524, 3,800)	29,969 (28,109, 31,625)	NA	NA	NA	NA	NA	NA	NA	NA
	Patient 2* (Stage IV, PFS ≥ 18 months)	Pooled	1,871 (1,787, 1,949)	7,961 (7,471, 8,460)	NA	NA	NA	NA	1,760 (1,734, 1,787)	4,150 (4,037, 4,259)	1,947 (1,906, 1,986)	5,255 (5,089, 5,415)
	Patient 3 (Stage I, PFS ≥ 18 months)	Cluster 2	NA	NA	NA	NA	NA	NA	4,149 (4,064, 4,241)	16,410 (15,796, 17,043)	NA	NA
		Cluster 1	NA	NA	NA	NA	NA	NA	2,368 (2,306, 2,429)	7,131 (6,813, 7,462)	NA	NA
		Pooled	2,921 (2,876, 2,966)	13,289 (12,897, 13,647)	NA	NA	NA	NA	2,708 (2,634, 2,788)	8,904 (8,447, 9,380)	1,961 (1,918, 2,002)	5,699 (5,491, 5,922)
Lung adenocarcinoma	Patient 1 {Stage IIIB}	Cluster 2	3,999 (3,921, 4,073)	15,664 (15,180, 16,128)	NA	NA	NA	NA	NA	NA	1,663 (1,612, 1,713)	4,179 (3,995, 4,375)
		Cluster 1	649 (616, 682)	1,230 (1,132, 1,341)	NA	NA	NA	NA	NA	NA	717 (675, 767)	1,680 (1,498, 1,869)
		Pooled	1,869 (1,761, 1,979)	6,489 (5,952, 7,050)	3,233 (3,156, 3,314)	9,846 (9,502, 10,176)	724 (692, 754)	1,479 (1,394, 1,569)	1,371 (1,314, 1,432)	3,200 (2,989, 3,417)	1,520 (1,464, 1,574)	3,801 (3,612, 3,993)
	Patient 2 {Stage IIB}	Cluster 2	2,778 (2,680, 2,871)	8,458 (8,041, 8,868)	NA	NA	2,097 (2,039, 2,160)	6,123 (5,856, 6,411)	NA	NA	1,898 (1,836, 1,968)	5,179 (4,899, 5,486)
		Cluster 1	831 (784, 880)	1,703 (1,535, 1,897)	NA	NA	586 (561, 612)	1,091 (1,024, 1,168)	NA	NA	649 (623, 676)	1,255 (1,190, 1,321)
		Pooled	1612 (1515, 1703)	4,411 (4,058, 4,763)	NA	NA	1200 (1134, 1266)	3,135 (2,917, 3,360)	684 (652, 715)	1,316 (1,232, 1,401)	1,128 (1,071, 1,188)	2,760 (2,549, 2,985)
Pancreatic adenocarcinoma	Patient 1 (Stage IV, OS = 21 months)	Cluster 2	4,315 (4,205, 4,421)	21,718 (20,860, 22,550)	2,549 (2,437, 2,654)	11,066 (10,341, 11,818)	NA	NA	NA	NA	NA	NA
		Cluster 1	1,510 (1,439, 1,578)	4,631 (4,314, 4,938)	616 (588, 645)	1,491 (1,393, 1,599)	NA	NA	NA	NA	NA	NA
		Pooled	3,323 (3,193, 3,458)	15,675 (14,823, 16,549)	1,423 (1,334, 1,527)	5,489 (4,933, 6,075)	NA	NA	NA	NA	NA	NA
	Patient 2 (Stage IIB, OS ≥ 45 months)	Cluster 2	2,235 (2,160, 2,306)	8,896 (8,437, 9,317)	1,382 (1,324, 1,442)	6,017 (5,635, 6,386)	NA	NA	NA	NA	NA	NA
		Cluster 1	997 (959, 1,039)	3,381 (3,173, 3,581)	779 (728, 831)	2,496 (2,246, 2,765)	NA	NA	NA	NA	NA	NA
		Pooled	1,614 (1,542, 1,682)	6,129 (5,715, 6,486)	1,086 (1,028, 1,142)	4,286 (3,965, 4,644)	NA	NA	NA	NA	NA	NA

Supplementary Note Table 2. (Continued)

Cancer type	Patient id	Cell cluster	Cell type							
			Myeloid		T cell		Natural killer cell		B cell	
			Average gene counts (95% CI)	Average total UMI counts (95% CI)	Average gene counts (95% CI)	Average total UMI counts (95% CI)	Average gene counts (95% CI)	Average total UMI counts (95% CI)	Average gene counts (95% CI)	Average total UMI counts (95% CI)
Colorectal adenocarcinoma	Patient 1 (Stage IVA, DFS = 4 months)	Cluster 3	NA	NA	NA	NA	NA	NA	NA	NA
		Cluster 2	2,984 (2,920, 3,054)	13,050 (12,518, 13,573)	NA	NA	NA	NA	NA	NA
		Cluster 1	535 (523, 548)	1,415 (1,372, 1,457)	NA	NA	NA	NA	NA	NA
		Pooled	1,787 (1,691, 1,877)	7,365 (6,853, 7,920)	1,211 (1,177, 1,243)	3,600 (3,473, 3,743)	NA	NA	1,102 (1,058, 1,149)	5,422 (5,012, 5,873)
	Patient 3 (Stage IVA, DFS ≥ 22 months)	Cluster 2	NA	NA	NA	NA	NA	NA	NA	NA
		Cluster 1	NA	NA	NA	NA	NA	NA	NA	NA
		Pooled	662 (627, 700)	2,054 (1,881, 2,231)	1,203 (1,177, 1,230)	4,377 (4,250, 4,505)	NA	NA	963 (911, 1,011)	3,633 (3,330, 3,936)
	Patient 2 (Stage IVA, DFS = 5 months)	Cluster 2	NA	NA	1470 1453, 1487	6184 (6086, 6285)	NA	NA	NA	NA
		Cluster 1	NA	NA	1077 (1050, 1106)	5,354 (5,164, 5,566)	NA	NA	NA	NA
		Pooled	659 (612, 709)	3086 (2776, 3381)	1,185 (1,157, 1,213)	5,582 (5,405, 5,774)	NA	NA	685 (655, 715)	3,643 (3,369, 3,950)
Hepatocellular carcinoma	Patient 1 (Stage IV, PFS < 5 months)	Cluster 2	NA	NA	NA	NA	NA	NA	NA	NA
		Cluster 1	NA	NA	NA	NA	NA	NA	NA	NA
		Pooled	NA	NA	1,318 (1,285, 1,350)	3,720 (3,561, 3,879)	NA	NA	NA	NA
	Patient 2 (Stage IV, PFS ≥ 18 months)	Pooled	1,406 (1,376, 1,434)	4,228 (4,085, 4,368)	1,303 (1,272, 1,340)	3,221 (3,079, 3,366)	NA	NA	1,105 (1,088, 1,122)	11,686 (11,426, 11,965)
	Patient 3 (Stage I, PFS ≥ 18 months)	Cluster 2	NA	NA	NA	NA	NA	NA	NA	NA
		Cluster 1	NA	NA	NA	NA	NA	NA	NA	NA
		Pooled	1,602 (1,571, 1,634)	5,879 (5,711, 6,057)	1,410 (1,373, 1,448)	4,590 (4,412, 4,782)	NA	NA	NA	NA
Lung adenocarcinoma	Patient 1 {Stage IIIB}	Cluster 2	1,293 (1,253, 1,329)	3,936 (3,776, 4,102)	NA	NA	NA	NA	NA	NA
		Cluster 1	393 (381, 407)	876 (838, 917)	NA	NA	NA	NA	NA	NA
		Pooled	1,233 (1,191, 1,271)	3,732 (3,573, 3,889)	584 (566, 602)	1,050 (1,011, 1,090)	NA	NA	544 (520, 568)	2,569 (2,278, 2,858)
	Patient 2 {Stage IIB}	Cluster 2	1,207 (1,164, 1,251)	3,504 (3,302, 3,721)	NA	NA	NA	NA	NA	NA
		Cluster 1	361 (352, 370)	728 (699, 754)	NA	NA	NA	NA	NA	NA
		Pooled	1,137 (1,089, 1,183)	3,275 (3,080, 3,482)	765 (745, 789)	1362 (1310, 1417)	NA	NA	762 (732, 796)	4,396 (4,083, 4,746)
Pancreatic adenocarcinoma	Patient 1 (Stage IV, OS = 21 months)	Cluster 2	3,213 (3,131, 3,292)	16,221 (15,618, 16,851)	NA	NA	NA	NA	NA	NA
		Cluster 1	1,460 (1,420, 1,504)	3,840 (3,688, 3,980)	NA	NA	NA	NA	NA	NA
		Pooled	1,788 (1,726, 1,851)	6,158 (5,746, 6,568)	1,531 (1,508, 1,554)	4,814 (4,716, 4,922)	1,651 (1,630, 1,671)	4,064 (4,001, 4,126)	1,352 (1,327, 1,378)	4,119 (4,022, 4,213)
	Patient 2 (Stage IIB, OS ≥ 45 months)	Cluster 2	2,210 (2,155, 2,271)	10,285 (9,860, 10,748)	NA	NA	NA	NA	NA	NA
		Cluster 1	890 (857, 926)	2,523 (2,344, 2,711)	NA	NA	NA	NA	NA	NA
		Pooled	1,960 (1,891, 2,030)	8,818 (8,327, 9,245)	936 (919, 953)	2,526 (2,467, 2,586)	1,169 (1,148, 1,191)	2,855 (2,763, 2,950)	927 (904, 950)	2,684 (2,586, 2,775)

DFS: disease free survival, PFS: progression free survival, OS: overall survival.
*: for a patient, if all cell types have one cluster each, only the results from the pooled cells of each cell type are shown.
NA: due to no cells or only one cell cluster in the corresponding cell type; for the latter, the results of gene counts and total UMI counts are shown in the "Pooled" position.

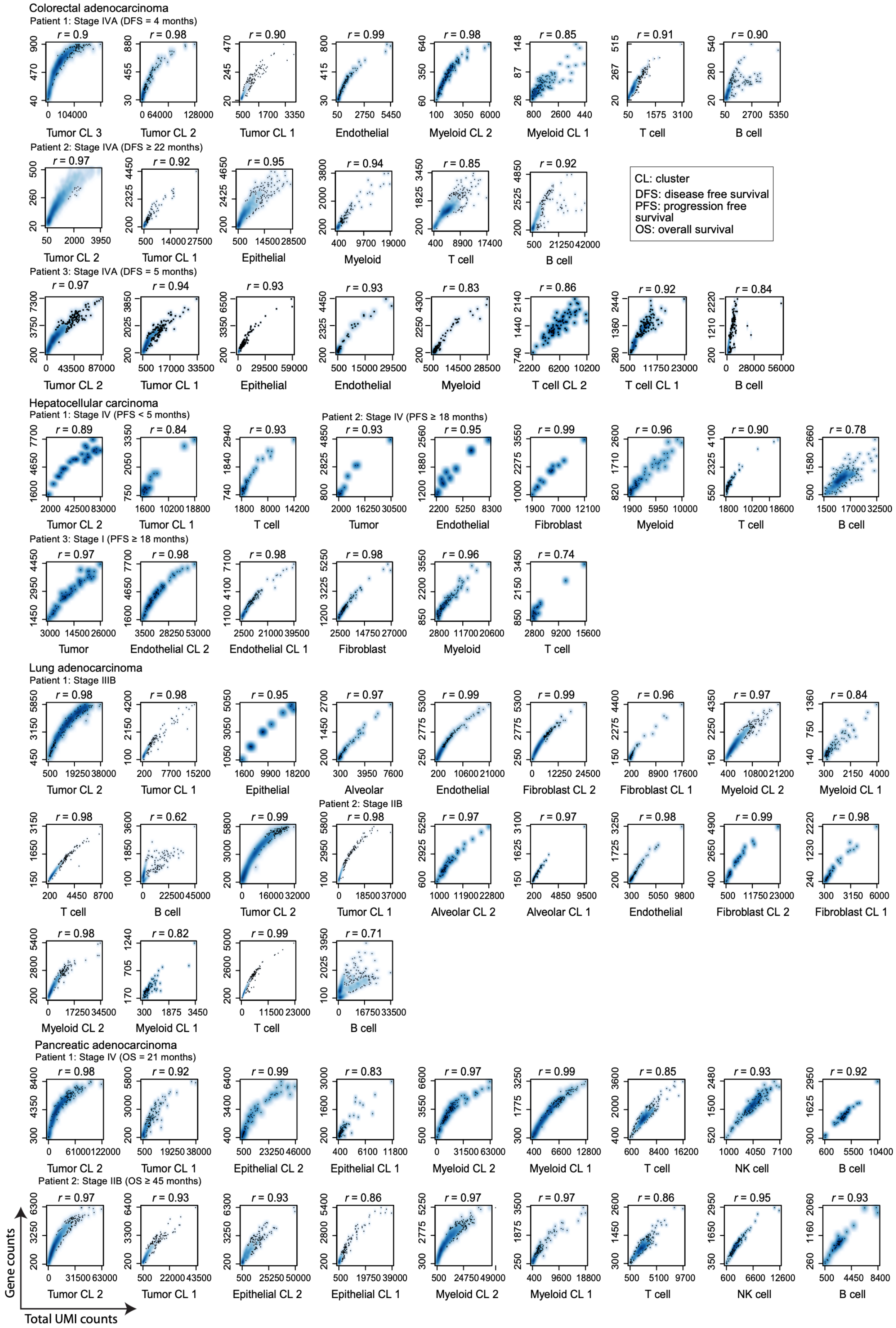

Supplementary Note Figure 3. Correlations between gene counts and total UMI counts. Smoothed scatter plots show the correlations between gene counts and total UMI counts in cell clusters from each patient sample. In each smoothed scatter plot, the Spearman correlation coefficient is labeled on the top (*r*).

Supplementary Note Table 3. Two-sided t-tests between the total UMI counts of high UMI tumor cell cluster and 3 times of the total UMI counts of low UMI tumor cluster within each patient across four cancer types. *P* values are adjusted by the Benjamini-Hochberg (BH) method.

Cancer type	Patient 1			Patient 2			Patient 3		
	No. of tumor cells	*P* value	μ_2/μ_1(95% CI)*	No. of tumor cells	*P* value	μ_2/μ_1(95% CI)	No. of tumor cells	*P* value	μ_2/μ_1(95% CI)
Colorectal adenocarcinoma	High UMI: 808<br>Low UMI: 2,426	$< 2x10^{-16}$	25 (23, 27)	High UMI: 1,696<br>Low UMI: 359	$9x10^{-13}$	3.7 (3.4, 4.2)	High UMI: 813<br>Low UMI: 528	0.22	3.2 (2.9, 3.4)
Hepatocellular carcinoma	High UMI: 26<br>Low UMI: 19	$9x10^{-7}$	10 (10,11)	NA	NA	NA	NA	NA	NA
Lung adenocarcinoma	High UMI: 497<br>Low UMI: 867	$< 2x10^{-16}$	13 (12, 14)	High UMI: 1,061<br>Low UMI: 1,586	$< 2x10^{-16}$	5.0 (4.4, 5.6)	NA	NA	NA
Pancreatic adenocarcinoma	High UMI: 462<br>Low UMI: 286	$6x10^{-10}$	4.7 (4.3, 5.0)	High UMI: 1,929<br>Low UMI: 1,942	$1x10^{-5}$	2.6 (2.4, 2.8)	NA	NA	NA

*μ_2 and μ_1 are the means of the total UMI counts from the tumor cell high-UMI cluster and tumor cell low-UMI cluster, respectively.

1.2.3 Cell cycle states of tumor cells

For each patient sample, we calculated the S score and G2M score for each tumor cell using the CellCycleScoring function in the Seurat package. Cells with either S score or G2M score > 0.2 are considered as cycling cells. Among the rest, cells with either 0 < S score ≤ 0.2 or 0 < G2M score ≤ 0.2 are defined as intermediate cells, and all the remaining cells are considered as non-cycling cells[13]. We examine if the high UMI tumor cell cluster is enriched with non-cycling cells by calculating an Odds Ratio (OR) = $\frac{No.of\ cycling\&intermediate\ cells\ in\ high\ UMI\ tumor\ cluster/\ No.of\ non{-}cycling\ cells\ in\ high\ UMI\ tumor\ cluster}{No.of\ cycling\&intermediate\ cells\ in\ low\ UMI\ tumor\ cluster/\ No.of\ non{-}cycling\ cells\ in\ low\ UMI\ tumor\ cluster}$ (also see the distribution of S and G2M scores in **Supplementary Note Figure 4**). An OR < 1 suggests enrichment of non-cycling cells. The results show that in all four patient samples (colorectal, liver, lung and pancreatic cancers) with worse survival outcomes, the high UMI tumor cells are enriched with non-cycling cells.

1.2.4 Gene set enrichment analysis in scRNA-seq data

We performed gene set enrichment analyses for the high and low UMI tumor cell clusters in scRNA-seq data. We first compiled a comprehensive set of signatures with 18,617 human gene sets (containing at least 4 genes) from the Molecular Signatures Database (MSigDB, v6.2)[47] and CellMarker[48]. Among them, 341 gene sets were annotated as stemness signatures based on the key word '_stem_' in their names. We quantified enrichment for high and low UMI tumor cells using the GeneOverlap R package (v1.24.0)[75]. GeneOverlap took the DE genes, a gene set and the background genome size (number of expressed genes in the scRNA-seq data expressed in ≥ 10 cells) as input, and gave a *P* value for the enrichment significance and Jaccard Index, which was calculated by the number of common genes in the DE gene list and the signature gene set divided by the union of them. *P* values were adjusted for multiple comparisons using the Benjamini-Hochberg (BH) correction. The DE genes between high UMI and low

tumor cells were obtained by the “FindMarkers” function from Seurat with Wilcoxon rank-sum test, based on criteria of adjusted P value < 0.1, genes expressed in ≥ 10 cells, and absolute $\log_2$(fold change) > 0.585 (1.5 fold change).

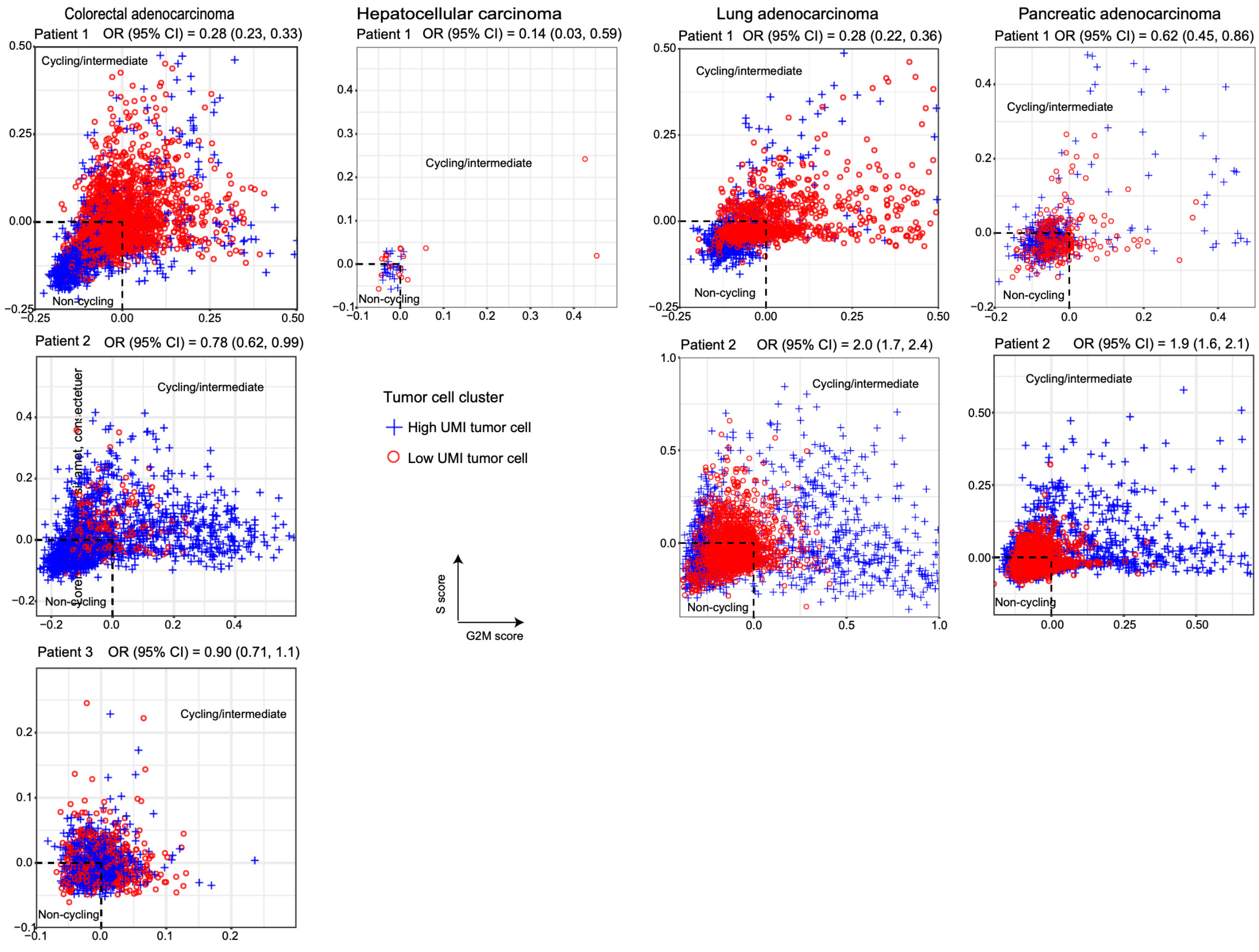

Supplementary Note Figure 4. Distribution of cycling/intermediate and non-cycling cells in tumor cells. Dashed lines separate cycling/intermediate and non-cycling cells. OR for high UMI vs. low UMI tumor cell clusters in cycling state and 95% confidence interval (CI) are labeled on the top.

2. TUMOR-SPECIFIC TOTAL MRNA EXPRESSION IN BULK SEQUENCING DATA

2.1. A mathematical model for tumor-specific total mRNA expression

For any group of cells, we use S to denote the average global mRNA transcript level per cell per haploid genome, which follows $S = \sum_{c=1}^{C} (\sum_{g=1}^{G} u_{gc} / p_c) / C$. Here p_c is the ploidy, *i.e.*, the number of copies of the

haploid genome in cell c. However, the cell level ploidy p_c is usually not measurable. Hence, in practice, we use average ploidy Ψ of the corresponding cell group to approximate it: $S \approx \sum_{c=1}^{C} \sum_{g=1}^{G} u_{gc} /(C\Psi)$. For non-tumor cells, which are commonly diploid, this assumption is assured.

2.1.1. Model

In the analysis of bulk RNAseq data from mixed tumor samples, we are interested in comparing tumor with non-tumor cell groups. We denote tumor cells by group T, and non-tumor cells by group N. Therefore, we define a tumor-specific total mRNA expression score (TmS) to reflect the ratio of total mRNA transcript level per haploid genome of tumor cells to that of the surrounding non-tumor cells, *i.e.*, $TmS_{tumor} = S_T / S_N$, simplified as TmS from here forward. It is necessary to calculate this ratio in order to cancel out technical effects presented in sequencing data that confound with both S_T and S_N. Let $T_g = \sum_{c=1}^{C_T} u_{gc}$ and $N_g = \sum_{c=1}^{C_N} u_{gc}$ denote the total number of mRNA transcripts of gene g across all cells from tumor and non-tumor cells, and $T_+ = \sum_{g=1}^{G} T_g$ and $N_+ = \sum_{g=1}^{G} N_g$, let C_T and C_N denote the total number of tumor and non-tumor cells, and let Ψ_T and Ψ_N represent the average ploidy of tumor and non-tumor cells, respectively. Under the assumption that the tumor cells have a similar ploidy, we can derive TmS without using single-cell-specific parameters as

$$TmS = \frac{T_+/(C_T\Psi_T)}{N_+/(C_N\Psi_N)}. \qquad \text{Eq.S1}$$

Here we further introduce a tumor-specific mRNA proportion $\pi = (\sum_{g=1}^{G} T_g) / (\sum_{g=1}^{G} T_g + \sum_{g=1}^{G} N_g)$ and a tumor cell proportion of tumor cells within a sample (termed "tumor purity") $\rho = C_T / (C_T + C_N)$. Note that deconvolution of just the gene expression data will not provide information on the total number of tumor cells and non-tumor cells, but only the sum of total mRNA expression across all cells of each cell type.

Using these parameters, we rewrite Eq.S1 as

$$TmS = \frac{\pi(1-\rho)\Psi_N}{(1-\pi)\rho\Psi_T}. \qquad \text{Eq.S2}$$

Additionally, we can define a ploidy-unadjusted *TmS* by removing the ploidy terms.

2.1.2. Estimation

It is common practice to assume the ploidy of non-tumor cells Ψ_N equals to 2[14,15]. Hence, we have

$$\widehat{TmS} = \frac{\hat{\pi}(1-\hat{\rho})2}{\hat{\rho}(1-\hat{\pi})\hat{\Psi}_T}. \qquad \text{Eq.S3}$$

In what follows, we use *TmS* to represent $\widehat{TmS}$, for the sake of simplicity.

Estimation of tumor-specific mRNA proportion π using high-throughput RNA sequencing has not been possible due to several technical and analytical factors including: 1) the need to account for technical artifacts introduced by varied library size, which currently involves normalization procedures across samples; 2) total mRNA transcripts per cell are confounded with technical artefacts so that normalization procedures adjust for both effects at once, consequently losing the ability to evaluate the downstream global transcriptome feature[16]; and 3) a limited focus on estimating cell proportions by popular methods[17–19].

Using deconvolution to partition tumor and non-tumor cells within the same sample under the same experimental conditions provides a mathematical means to cancel out the effect of technical artefacts while maintaining the effect of cell-type-specific total mRNA counts. We use the DeMixT model[20] to estimate π. For sample i and across any gene g, we have

$$Y_{ig}=\pi_i T'_{ig}+(1-\pi_i)N'_{ig} \qquad \text{Eq.S4}$$

where Y_{ig} represents the scale normalized expression matrix from mixed tumor samples, T'_{ig} and N'_{ig} represent the normalized relative expression of gene g within tumor and surrounding non-tumor cells, respectively. The estimated $\hat{\pi}$ is the quantity desired for Eq.S3.

Computational deconvolution methods, e.g., ASCAT[14] and ABSOLUTE[15], have been developed to perform allele-specific copy number analysis and to estimate tumor purity ρ and ploidy Ψ_T from tumor DNA sequencing data. Such statistical methods jointly model the distribution of $logR$ and B allele (or variant allele) frequency (BAF) across germline SNPs, with tumor purity and allele-specific copy number as parameters of interest. Then the tumor purity and ploidy (the average tumor copy number) can be estimated through minimizing the loss function or maximizing the likelihood. Below, we provide a detailed description for these methods using the ASCAT model as an example.

Sequence read counts at known SNP loci were computed from tumor DNA sequencing data. The $logR_i$ can be computed from the total read counts in the tumor versus normal for the ith SNP, which provides information on the ratio of total copy number between the tumor and the normal. Specifically, $logR_i$ can be expressed as[14]

$$logR_i = \gamma log_2\left(\frac{2(1-\rho)+\rho\left(n_{A,i}+n_{B,i}\right)}{2(1-\rho)+\psi_T}\right), \qquad \text{Eq.S5}$$

where ρ is the tumor purity, ψ_T is the tumor ploidy, γ is a constant depends on which DNA sequencing technology is used. $n_{A,i}$ and $n_{B,i}$ stand for the allele-specific copy number of A allele and B allele for the ith SNP in tumor cells, respectively.

On the other hand, allelic imbalance can be inferred from the BAF_i for ith SNP. The BAF_i can be expressed as[14]

$$BAF_i = \frac{1\text{-}\rho+\rho n_{B,i}}{2(1\text{-}\rho)+\rho(n_{A,i}+n_{B,i})}. \quad \text{Eq.S6}$$

Based on Eq.S5 and Eq.S6, the allele-specific copy number can be expressed as a function of the tumor purity and ploidy. Specifically, we have

$$\hat{n}_{A,i} = \frac{\rho\text{-}1+2^{\frac{logR_i}{\gamma}}(1-BAF_i)(2(1\text{-}\rho)+\Psi_T)}{\rho};$$

$$\hat{n}_{B,i} = \frac{\rho\text{-}1+2^{\frac{logR_i}{\gamma}}BAF_i(2(1\text{-}\rho)+\Psi_T)}{\rho}.$$

Allele-specific piecewise constant fitting (ASPCF)[21] was then applied to both $logR_i$ and BAF_i simultaneously, which enforced the change points to occur at the same genomic locations. Consequently, a segmentation of the genome was obtained, each segment corresponding to a genomic region between two adjacent change points. Using the ASPCF smoothed $logR_i$ and BAF_i, the final values for $\hat{\rho}$ and $\hat{\Psi}_T$ were obtained through the optimization, such that the allele-specific copy number estimates $\hat{n}_{A,i}$ and $\hat{n}_{B,i}$ were as close to nonnegative integers as possible for germline heterozygous SNPs.

2.2. Improved estimation using DeMixT

Many computational deconvolution methods have been developed to estimate cell type proportions through transcriptome data; however, most of them focus on the cellular proportion and not the global gene expression level of each cell type, due to lack of appropriate normalization approaches. The DeMixT[20] model is unique in aiming to estimate the global tumor-specific gene expression level relative to the normal reference in the context of admixed tumor samples. ISOpure[22] is the other model that presents similar objectives as the DeMixT model. The following issues and our proposed solution are generally applicable to both models.

The identifiability of model parameters is a major issue for high dimensional models. Due to technical limitations, given a certain amount and quality of experimental data, not all model parameters are guaranteed for unambiguous estimation. Frequently, only a subset of model parameters are identifiable based on the available data, with the rest of the parameters considered unidentifiable. Confidence intervals can be derived for identifiable parameters, which contain the true value of the parameter with a desired probability[23]. Fortunately, with the DeMixT model, there is hierarchy in model identifiability in which the cell-type specific global gene expression proportions (π) are the most identifiable parameters, requiring only a subset of genes with identifiable expression distributions. Therefore, our goal is to select an appropriate set of genes as input to DeMixT that optimizes the estimation of π. In general, genes are expressed at different levels, which, due to different numerical ranges, can affect estimation of π. We found that including genes that are not differentially expressed between the tumor and non-tumor components within the bulk sample, or genes with large variance in expression within the non-tumor component, can introduce large biases into the estimated π. On the other hand, the tumor component is hidden in the mixed tumor samples, hence preventing a DE analysis between mixed and normal samples from finding the best genes. By applying a profile-likelihood based approach to detect the identifiability of model parameters[24], we systematically evaluated the identifiability for all available genes based on the data, and selected the most identifiable genes for the estimation of π. As a result, the accuracy of the estimated proportions has been improved. As a general method, the profile-likelihood based gene selection strategy can be extended to any method that uses maximum likelihood estimation. We also employed an additional virtual spike-in strategy to balance proportion distributions which further improved model identifiability.

2.2.1. Likelihood model for DeMixT

In the DeMixT model[20] (Eq.S4), we assumed that the observed expression level Y_{ig} is a linear combination of two hidden components T_{ig} (tumor, in place of T'_{ig} from now on) and N_{ig} (non-tumor, in place of N'_{ig} from now on), where gene g = 1,2,…,G, sample i = 1,2,…,M , and π_i is the tumor-specific total mRNA

expression proportions. We assume each hidden component follows the $\log_2$-normal distribution, *i.e.*, $T_{ig} \sim LN\left(\mu_{Tg}, \sigma^2_{Tg}\right)$ and $N_{ig} \sim LN\left(\mu_{Ng}, \sigma^2_{Ng}\right)$.

Fitting the deconvolution model in Eq.S1 can be formally defined as an optimization problem that seeks to identify optimal estimates for sample-level, tumor-specific mRNA proportions π_i, and gene-level parameters. The full parameter set is denoted by $(\boldsymbol{\pi},\boldsymbol{\mu_T},\boldsymbol{\sigma_T})$, where $\boldsymbol{\pi} = (\pi_1,\pi_2,\ldots,\pi_M)$, $\boldsymbol{\mu_T} = (\mu_{T1}, \mu_{T2},\ldots, \mu_{TG})$, $\boldsymbol{\sigma_T} = (\sigma_{T1}, \sigma_{T2},\ldots,\sigma_{TG})$. The full log-likelihood of the DeMixT model can be written as

$$l\left(\boldsymbol{\pi},\boldsymbol{\mu_T},\boldsymbol{\sigma_T}\right)=\sum_{i=1}^{M}\sum_{g=1}^{G} log(f(Y_{ig}|\pi_i,\mu_{Tg},\sigma_{Tg})),$$

where $f\left(Y_{ig}|\pi_i,\mu_{Tg},\sigma_{Tg}\right)=\frac{1}{2\pi\sigma_{Ng}\sigma_{Tg}}\int_0^{Y_{ig}}\frac{1}{t(Y_{ig}-t)}exp\left(-\frac{(log2(t)-\mu_{Ng}-log2(1-\pi_i))^2}{2\sigma^2_{Ng}}-\frac{(log2(Y_{ig}-t)-\mu_{Tg}-log2(\pi_i))^2}{2\sigma^2_{Tg}}\right)dt$.

The DeMixT model applies an optimization method, iterated conditional modes (ICM)[25], to maximize the full log-likelihood function and estimate all distribution parameters $(\boldsymbol{\mu_T},\boldsymbol{\sigma_T})$ and proportions $\boldsymbol{\pi}$.

2.2.2. Optimized model identifiability

Based on the most stringent definition, for a parametric model $l(\boldsymbol{Y} \mid \theta)$, θ is identifiable if, $l(\boldsymbol{Y} \mid \theta_1) = l(\boldsymbol{Y} \mid \theta_2) => \theta_1 = \theta_2$. However, this rigorous identifiability is difficult to validate for a general high-dimensional and non-convex model, which is the case for the DeMixT model. Thus, for a parameter θ, we use the confidence interval $[\theta^-, \theta^+]$ to measure its identifiability[24].

In the DeMixT model, if we select genes with small confidence intervals of μ_{Tg} based on profile likelihood, which indicate high identifiability, the corresponding gene *g* will be more stable and reliable, so will the inferred tumor-specific mRNA proportions ($\boldsymbol{\pi}$). As a result, the length of confidence interval of μ_{Tg} serves as an estimable quantity with which we can evaluate the gene *g*'s identifiability and prioritize genes to increase the estimation quality of π_i, μ_{Tg}, σ_{Tg}.

The profile likelihood is preferred to compute confidence intervals of parameters that often have better small-sample properties than those based on asymptotic standard errors calculated from the full likelihood[26]. Assume the *k*th gene's mean parameter μ_{Tk} is the parameter of interest. The definition of the profile log-likelihood function of μ_{Tk} is:

$$l_{\mu_{Tk}}\left(\mu_{Tk}=x|\boldsymbol{\pi},\boldsymbol{\mu_T},\boldsymbol{\sigma_T}\right) = \max_{\pi_i,\mu_{Tg},\sigma_{Tg},\sigma_{Tk}} \{\sum_{i=1}^{M}[\sum_{g\neq k}^{G} log\ \left(f\left(\pi_i,\mu_{Tg},\sigma_{Tg}\right)\right) +log\ \left(f\left(\pi_i,\mu_{Tk}=x,\sigma_{Tk}\right)\right)\ ]\}$$

The confidence interval of a profile likelihood function can be constructed through inverting a likelihood-ratio test[27]. Assume the null hypothesis as H_0: $\mu_{Tk} = x$, and the maximum likelihood estimator of (π_i, μ_{Tg},

σ_{Tg}) are ($\hat{\pi}_i$, $\hat{\mu}_{Tg}$, $\hat{\sigma}_{Tg}$). The null hypothesis will not be rejected at the α level of significance if and only if $2\left[l(\hat{\boldsymbol{\pi}}, \hat{\boldsymbol{\mu}}_{\boldsymbol{T}}, \hat{\boldsymbol{\sigma}}_{\boldsymbol{T}}) - l_{\mu_{Tk}}(\mu_{Tk} = x \mid \hat{\boldsymbol{\pi}}, \hat{\boldsymbol{\mu}}_{\boldsymbol{T}}, \hat{\boldsymbol{\sigma}}_{\boldsymbol{T}})\right] \leq \chi^2_{1-\alpha}(1)$, where $\chi^2_{1-\alpha}(1)$ stands for $1-\alpha$ percentile of the χ^2 distribution with degrees of freedom equal to 1. Since the maximized likelihood $l(\hat{\boldsymbol{\pi}}, \hat{\boldsymbol{\mu}}_{\boldsymbol{T}}, \hat{\boldsymbol{\sigma}}_{\boldsymbol{T}})$ and model parameters $\hat{\boldsymbol{\pi}}, \hat{\boldsymbol{\mu}}_{\boldsymbol{T}}, \hat{\boldsymbol{\sigma}}_{\boldsymbol{T}}$ can be estimated by running the DeMixT model on all available gene sets, for any given x, we are able to investigate the profile log-likelihood function $l_{\mu_{Tk}}(\mu_{Tk} = x \mid \hat{\boldsymbol{\pi}}, \hat{\boldsymbol{\mu}}_{\boldsymbol{T}}, \hat{\boldsymbol{\sigma}}_{\boldsymbol{T}})$. Consequently, we can estimate the lower and upper bounds of the confidence interval $[\mu^-_{Tk}, \mu^+_{Tk}]$ as

$$\mu^-_{Tk} = \min_x \{x \mid 2\left[l(\hat{\boldsymbol{\pi}}, \hat{\boldsymbol{\mu}}_{\boldsymbol{T}}, \hat{\boldsymbol{\sigma}}_{\boldsymbol{T}}) - l_{\mu_{Tk}}(\mu_{Tk} = x \mid \hat{\boldsymbol{\pi}}, \hat{\boldsymbol{\mu}}_{\boldsymbol{T}}, \hat{\boldsymbol{\sigma}}_{\boldsymbol{T}})\right] \leq \chi^2_{1-\alpha}(1)\}$$
$$\mu^+_{Tk} = \max_x \{x \mid 2\left[l(\hat{\boldsymbol{\pi}}, \hat{\boldsymbol{\mu}}_{\boldsymbol{T}}, \hat{\boldsymbol{\sigma}}_{\boldsymbol{T}}) - l_{\mu_{Tk}}(\mu_{Tk} = x \mid \hat{\boldsymbol{\pi}}, \hat{\boldsymbol{\mu}}_{\boldsymbol{T}}, \hat{\boldsymbol{\sigma}}_{\boldsymbol{T}})\right] \leq \chi^2_{1-\alpha}(1)\}$$

Following the same procedure, we can derive the confidence interval of μ_{Tk} for all available genes.

In real data analysis, calculating the actual profile likelihood function of μ_{Tk} across all 20,000 genes is generally infeasible due to computational limits. An asymptotic approximation is necessary in order to quickly evaluate the profile likelihood function. If the measurement noise is small and the sample size is large enough, asymptotic confidence intervals are good approximations of the actual confidence intervals[24]. The asymptotic profile likelihood function can be derived from the observed Fisher information of the log likelihood, denoted as $H(\hat{\boldsymbol{\pi}}, \hat{\boldsymbol{\mu}}_{\boldsymbol{T}}, \hat{\boldsymbol{\sigma}}_{\boldsymbol{T}})$. Then the asymptotic α level confidence interval of μ_{Tk} can be written as follows[24]

$$\mu_{Tk}^{\pm} = \widehat{\mu_{Tk}} \pm \sqrt{2\chi^2_{1-\alpha}(1)\, H(\hat{\boldsymbol{\pi}}, \hat{\boldsymbol{\mu}}_{\boldsymbol{T}}, \hat{\boldsymbol{\sigma}}_{\boldsymbol{T}})^{-1}_{k,k}}\,. \qquad \text{Eq.S7}$$

Validation. We compared the actual profile likelihood function with the asymptotic profile likelihood function for a random set of 20 genes in real data (the TCGA prostate adenocarcinoma dataset) and observed good performance of the approximate profile likelihoods (**Supplementary Note Figure 5**). With 20 randomly selected genes, we calculated the root mean squared error (RMSE) between the confidence intervals from the true and asymptotic profile likelihoods is 0.05.

Gene selection score. We now introduce a metric, the gene selection score, which for gene k is the width of the asymptotic 95% profile likelihood-based confidence interval of μ_{Tk} for gene k

$$\textit{gene selection score}_k = 2\sqrt{2\chi^2_{0.05}(1)\, H(\hat{\pi}, \hat{\mu}_T, \hat{\sigma}_T)^{-1}_{k,k}}. \qquad \text{Eq.S8}$$

Genes with a lower score have a smaller confidence interval, hence higher identifiability in their corresponding parameters. Genes are ranked based on the gene selection score from the smallest to the largest. A subset of genes that are ranked on top will be used for parameter estimation. In the DeMixT R

package (freely available from Bioconductor), our proposed profile-likelihood based gene selection approach is included as function “DeMixT_GS”.

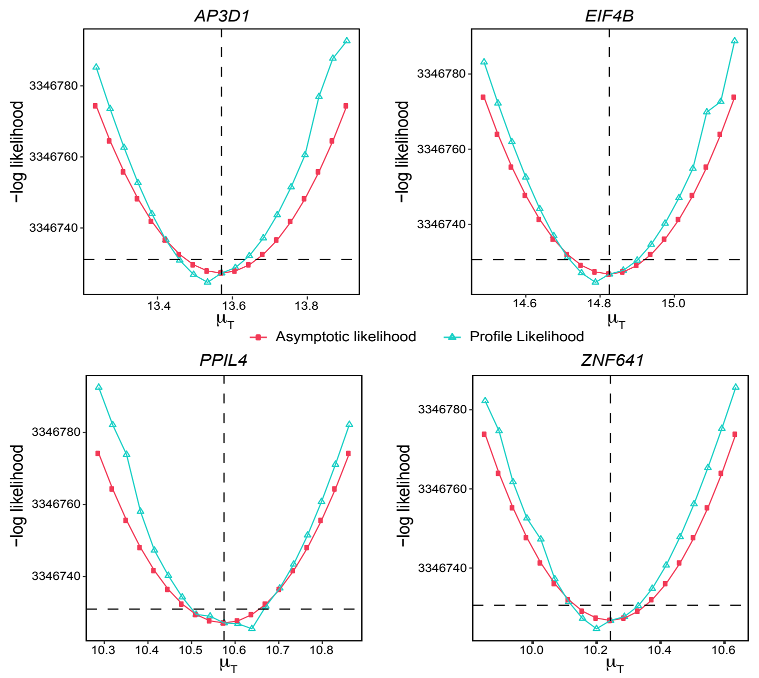

Supplementary Note Figure 5. Asymptotic profile likelihoods for 4 genes using 259 samples from the TCGA prostate cancer dataset. Comparison of asymptotic and actual profile likelihoods of μ_T for 4 randomly selected genes in the TCGA prostate adenocarcinoma data. The red curve shows the true profile likelihood of the corresponding parameter. The blue curve shows an asymptotic approximation of the profile likelihood of the corresponding parameter.

2.2.3. A simulation study for profile-likelihood based gene selection

The DeMixT model assumes every gene g has a shared mean (μ_{Tg}) and variance (σ_{Tg}) parameters across all tumor samples. However, in real data, this assumption will be violated in selected genes, due to the fact that some genes are significantly differentially expressed in different subtypes of the cancer. For example, the PAM50 genes are known to be differentially expressed in different molecular subtypes in breast cancer, e.g., Basal, Her2, LumA, and LumB subtypes. Therefore, our simulation aimed to assess the performance of the proposed gene selection method in finding genes that best follow the DeMixT model. The detailed simulation design is described below.

We simulated gene expression of 269 mixed samples and 100 normal references with 10,000 genes, mimicking the real data scenario presented in the TCGA prostate adenocarcinoma dataset. The true π were set as the tumor cell proportions derived from ASCAT. We generated the expression of 10,000 genes under four scenarios: 1) genes that are consistently differentially expressed between the tumor

and normal components; 2) genes that are differentially expressed across subtypes of tumor samples; 3) genes that are consistently expressed similarly between the tumor and normal components; 4) genes that are expressed with large variances. Specifically, for scenario 1), we generated 6,000 genes for the pure tumor T_{ig} and normal references N_{ig} with distributions $log_2(T_{ig}) \sim N(\mu_{Tg}, \sigma^2_{Tg})$ and $log_2(N_{ig}) \sim N(\mu_{Ng}, \sigma^2_{Ng})$, where i denotes sample, $i=1, \cdots, M$. We simulated $\mu_{Ng}, \mu_{Tg} \sim N(7, 1.5^2)$, and σ_{Ng}, σ_{Tg} were sampled with replacement from the observed standard deviations from the normal samples of TCGA prostate adenocarcinoma. For scenario 2), we generated additional 2,000 subtype genes with samples spit into equal-sized subgroups M_1, M_2, and M_3 with corresponding $\mu_{T_{1g}} \sim N(5, 1.5^2)$, $\mu_{T_{2g}} \sim N(7, 1.5^2)$, $\mu_{T_{3g}} \sim N(9, 1.5^2)$ and $M = M_1 + M_2 + M_3$. Then we generate the expression with $log_2(N_{ig}) \sim N(\mu_{Ng}, \sigma^2_{Ng})$, $log_2(T_{ig}) \sim N(\mu_{T_kg}, \sigma^2_{Tg})$, $k=1, 2, 3$, $i \in M_k$. For scenario 3), we generated 1,000 genes with strictly equal mean expression for pure tumor and normal reference, where $\mu_{Ng} = \mu_{Tg} \sim N(7, 1.5^2)$, $log_2(T_{ig}) \sim N(\mu_{Tg}, \sigma^2_{Tg})$ and $log_2(N_{ig}) \sim N(\mu_{Ng}, \sigma^2_{Ng})$. For scenario 4), we generated the remaining 1,000 genes with large variances, where $\mu_{Ng}, \mu_{Tg} \sim N(7, 1.5^2)$, $\sigma_{Ng} = \sigma_{Tg} = 1.5$, and the expression profile follow $log_2(T_{ig}) \sim N(\mu_{Tg}, \sigma^2_{Tg})$, $log_2(N_{ig}) \sim N(\mu_{Ng}, \sigma^2_{Ng})$. Then we mixed the T_{ig} and N_{ig} component expression linearly at the generated π according to the DeMixT model: $Y_{ig} = \pi_i T_{ig} + (1-\pi_i) N_{ig}$, where $G=10,000$, $M=269$. Our proposed gene selection method ("DeMixT_GS") successfully ranked genes from scenario 1) much higher than others, whereas a routine DE analysis using the two-sided t-test statistic between mixed tumor and normal samples failed to identify these genes (**Extended Data Fig. 4d**). Across simulations where we selected 100, 250, 1500, 2500, and 8000 genes, "DeMixT_GS" always outperformed "DeMixT_DE" in estimating proportions (**Supplementary Note Figure 6a**). The dip test[28] was used to measure the unimodality of the distribution of gene expression. This test statistic was designed to test multimodality of a random variable based on the maximum difference between the empirical distribution and the unimodal distribution of all observed data points (**Supplementary Note Figure 6b**). It suggests that the proposed gene selection method successfully ranked subtype-specific genes lower than the DE method.

Optimal selection of genes. We also observed that the number of genes selected by "DeMixT_GS" influences the performance of DeMixT. The accuracies of π estimation based on 100, 250, 1,500, 2,500 and 8,000 genes selected by the proposed "DeMixT_GS" were compared. **(Supplementary Note Figure 6a, c)**. Accurate π estimation, as measured by the RMSE, was achieved with 1,500 or 2,500 genes. In real data, we used either the top 1,500 or top 2,500 genes to estimate π.

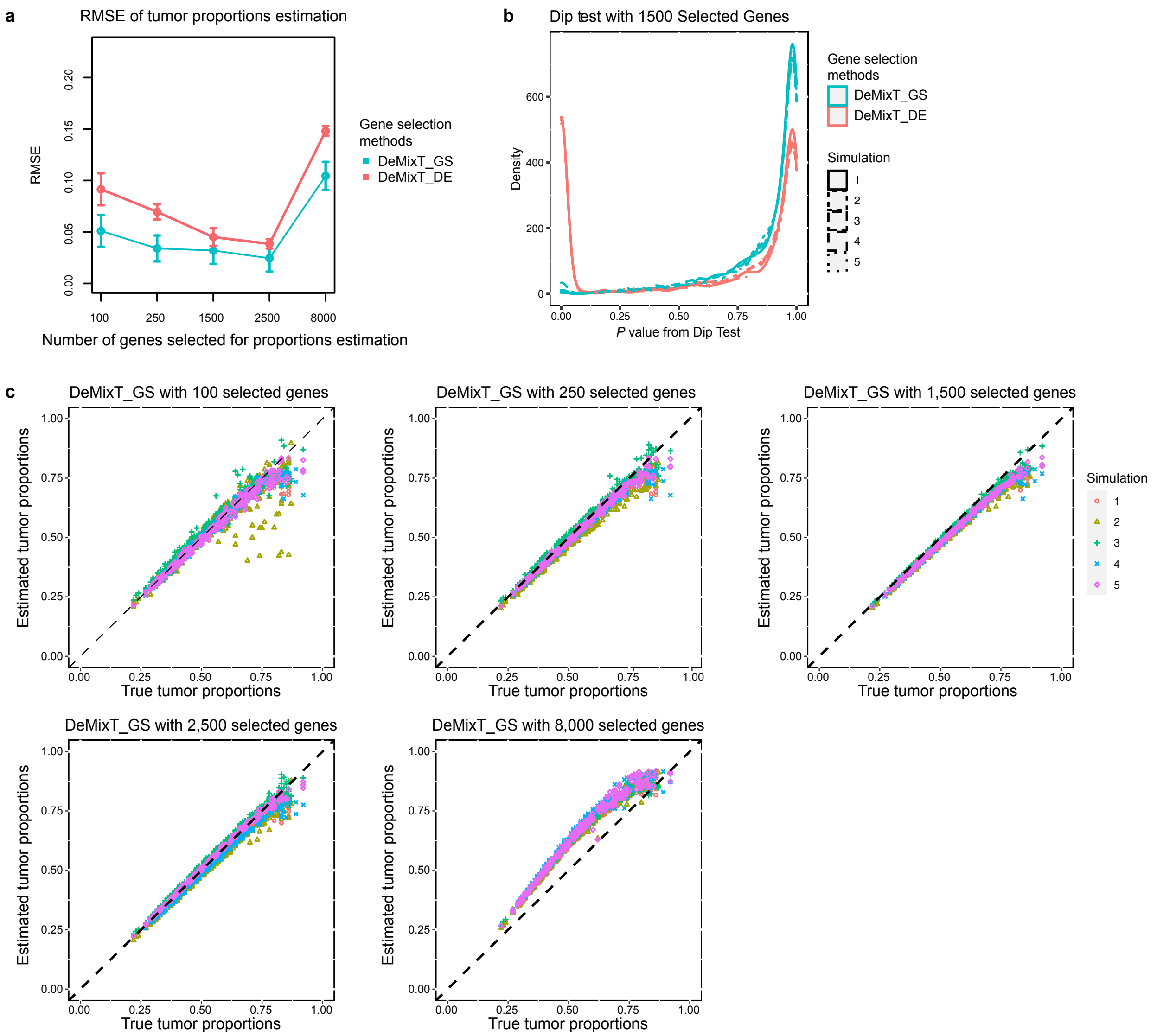

Supplementary Note Figure 6. Profile-likelihood based gene selection (DeMixT_GS) improves tumor-specific total mRNA expression proportions estimation. a, Root Mean Square Error (RMSE) was calculated for 5 simulated scenario. The center points represent the mean and the bound of error bars represent the mean +/- one standard error of RMSE. **b**, Density of *P* values based on a dip test for selected genes by "DeMixT_GS" and "DeMixT_DE" methods. The dip test was applied to indicate the distribution of gene expression for selected genes based on the "DeMixT_GS" and "DeMixT_DE" methods, respectively. A small *P* value of the dip test suggests the corresponding gene is not unimodally distributed, which violates the model assumption of $\log_2$-normal distribution across samples. **c**, Scatter plot of true versus estimated tumor-specific total mRNA expression proportions using "DeMixT_GS" method with different numbers of top-ranking genes with the smallest gene selection score.

2.2.4. Virtual spike-ins to improve identifiability and a simulation study

When the true proportions are skewed towards the high end (*i.e.*, median above 0.5), which is expected to occur frequently in real data (tumor samples with a low percentage of tumor cells are already discarded), the DeMixT estimation procedure, after careful gene selection, tends to slightly underestimate the high proportions. We hypothesize that by shifting the mode of the π distribution close to 0.5, the issue of underestimation will be alleviated. To achieve this, we simulate additional "mixed tumor" samples, *i.e.* spike-ins, with close to 0% of π, so that there are roughly the same number of samples with tumor proportions below and above 50%, *i.e.*, $S_P + |\{i \mid \rho_i < 0.5\}| \cong |\{i \mid \rho_i \geq 0.5\}|$, where S_P represents the number of spike-ins, ρ_i represent tumor purity of sample *i*, and the $|\cdot|$ represent cardinality of a set. For the cancer type whose median tumor purity is below 0.5, we set S_P at 5. The spike-ins are generated based on gene expression profiles observed from the input data of normal reference samples.

We simulated 100 mixed samples and 100 normal reference samples with 8,000 genes based on simulation settings described in **Section 2.2.3** with five replicates. Tumor-specific mRNA proportions were simulated from a normal distribution (mean = 0.55, SD = 0.2) and truncated at endpoints of 0.05 and 0.95. $\mu_{Ng}, \mu_{Tg} \sim N(7, 1.5^2)$ and $\sigma_{Ng}, \sigma_{Tg} \sim U(0.1, 0.8)$. The expression level of spike-ins is denoted as P_{jg}. We simulate $P_{jg} \sim LN(\hat{\mu}_{Ng}, \hat{\sigma}^2_{Ng})$, for gene $g = 1, 2, \ldots, G$ and sample $j = 1, 2, \ldots, S_P$. The spike-ins were then combined with mixed tumor samples. We ran DeMixT on the combined samples while fixing π for the spike-ins at 0.01. We found adding spike-ins can reduce biases in the estimation of tumor-specific mRNA proportions (**Supplementary Note Figure 7a**), as demonstrated by the improved gene selection scores (the smaller the better) for the top-ranking genes (**Supplementary Note Figure 7b**).

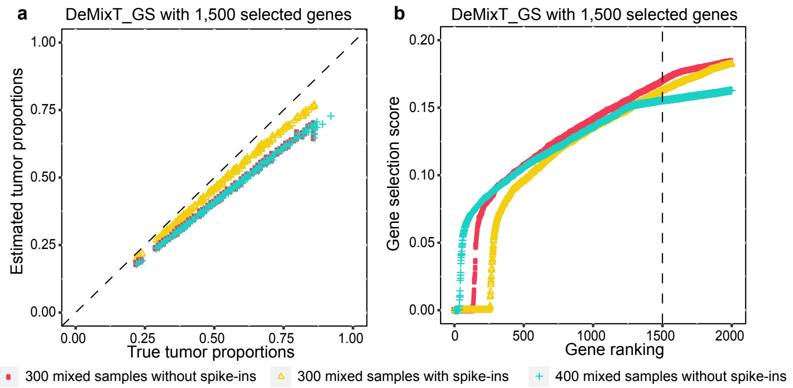

Supplementary Note Figure 7. Adding spike-ins reduces systematic bias of estimated tumor-specific mRNA proportions at a high end. a, Scatter plot of true versus estimated tumor-specific mRNA proportions using "DeMixT_GS" method under different strategies of adding spike-ins. **b**, Distribution of gene selection score to top 2,000 genes under different strategies of adding spike-ins, where the x-axis represents the rank of genes sorted by gene selection score from low to high and the y-axis represents the estimated gene selection score for the corresponding genes.

2.3. Tumor-specific total mRNA expression in patient samples

2.3.1. Datasets

The Cancer Genome Atlas data

Publicly available transcriptome profiling HT-seq raw read counts from 7,054 tumor samples from 15 cancer types in TCGA (breast adenocarcinoma, bladder urothelial carcinoma, colorectal cancer (colon adenocarcinoma + rectum adenocarcinoma), head-and-neck squamous cell carcinoma, kidney chromophobe, kidney renal clear cell carcinoma, kidney renal papillary cell carcinoma, liver hepatocellular carcinoma, lung adenocarcinoma, lung squamous cell carcinoma, pancreatic adenocarcinoma, prostate adenocarcinoma, stomach adenocarcinoma, thyroid carcinoma, uterine corpus endometrial carcinoma) were downloaded from the GDC data portal (v14.0)[29] (https://portal.gdc.cancer.gov/). They were generated through the standard RNAseq analysis pipeline (https://docs.gdc.cancer.gov/Data/Bioinformatics_Pipelines/Expression_mRNA_Pipeline/) by aligning reads to the GRCh38 reference genome and then by quantifying the mapped reads. Clinical annotation data including overall survival (OS), progression-free interval (PFI), pathologic stage, age, and sex of patients across 15 cancer types was downloaded from the GDC data portal (https://gdc.cancer.gov/about-data/publications/pancanatlas). Somatic mutation data of the 15 cancer types were downloaded from the re-annotated mutation annotation file (MAF) format at the GDC (https://gdc.cancer.gov/about-data/publications/mc3-2017). ABSOLUTE tumor purity and ploidy data were downloaded from Aran *et al*[30]. ASCAT tumor purity and ploidy data were downloaded from Alexandrov *et al*[31]. Driver mutation and indels annotation were downloaded from the TCGA pan-cancer driver mutation database: http://intogen.org/download version 2016.5[32]. NarrowPeak format ATAC-seq data for TCGA samples was obtained from Corces *et al*[33]. NarrowPeak files were annotated using the R package chipseeker[34]. Peaks outside of promoter regions (-2kb to 1kb of transcription start sites) were excluded. For breast adenocarcinoma, molecular subtype, triple negative status, status of hormone receptor, were obtained from Koboldt *et al*[35]. Copy number alternation status of *MYC* and *PVT1,* called by GISTIC[36] using the SNP6 DNA microarray data from breast carcinoma in TCGA, were obtained from cBioPortal (https://www.cbioportal.org/)[37]. For prostate adenocarcinoma, Gleason scores were obtained from Abeshouse *et al*[38]. For head and neck squamous cell carcinoma, HPV status was obtained from Lawrence *et al*[39]. In renal papillary carcinoma, molecular subtypes were obtained from Linehan *et al*[40].

International Cancer Genome Consortium – Early-Onset Prostate Cancer data[41]

Matched RNAseq and whole genome sequencing (WGS) data from 121 tumor samples and 9 adjacent normal samples from 96 patients, the corresponding clinical data including biochemical recurrence (BCR), and Gleason scores were downloaded from an early-onset (treatment age < 55) prostate cancer patient cohort (ICGC-EOPC)[41]. Among these 96 patients, there were 13 with Gleason score = 3+3, 58 with

Gleason score = 3+4, 11 with Gleason score = 4+3, 1 with Gleason score = 4+4, 6 with Gleason Score = 4+5, 6 with Gleason score = 5+4, and 1 with Gleason Score = 5+5.

For the ICGC-EOPC dataset, the gene expression read counts from the ultra-deep total RNAseq and relevant clinical data of 121 tumors samples from 96 patients were obtained from Gerhauser *et al*[41]. RNA reads were aligned to the human GRCh37 reference genome using BWA and SAMtools. Uniquely mapped reads were annotated using Ensembl v62. DNA library preparation and WGS was performed on Illumina sequencers[42] with a median insert size of 310 bp (SD = 57 bp) and a median WGS coverage of 61-fold for tumor and 38-fold for germline control samples. WGS data was aligned to the GRCh37 reference genome using BWA-MEM[43] according to Pan Cancer Analysis of Whole Genomes (PCAWG) protocol (https://doi.org/10.1101/161638).

METABRIC data

RNA expression arrays profiled by Illumina HT-12 v3 and matched DNA arrays profiled by Affymetrix SNP 6.0 from tumor tissues of 1,992 female patients with breast cancer in the METABRIC[44] cohort were downloaded from EGA (https://ega-archive.org) with Study ID EGAS00000000083. This cohort were split into a discovery set (997 patients) and a validation set (995 patients) by the original study[44]. 144 adjacent normal tissue expression arrays, each from one patient, are also available in this cohort. Clinical information including disease free survival and treatment was downloaded from cBioPortal (https://www.cbioportal.org/study/summary?id=brca_metabric).

TRACERx data

Matched RNAseq and WES data from multi region tumor samples of 64 patients in TRACER cohort were obtained[45–47]. WES data was performed on DNA samples, see details in Jamal-Hanjani *et al.*[45]. For RNA sequencing data, the STAR package[48] (version 2.5.2.b) was used to perform alignment and map reads to human hg19 reference genome. RNA count data was generated and quantified by the RSEM package[49] (version 1.3.0). Clinical information including disease free survival was downloaded from Jamal-Hanjani *et al.*[45].

2.3.2. TCGA

2.3.2.1. Data processing

To estimate the tumor-specific mRNA proportions (π) for each sample, we used the two-component mode of DeMixT for 15 TCGA cancer types where sufficient normal reference samples were available (the minimum number of normal samples is seven). For each cancer type, the following quality control was performed on both the tumor and normal samples to remove any suspicious samples. For each gene, we first used the Wilcoxon rank-sum test to test for differential expression between normal and tumor samples. The top 1,000 genes with the smallest P values were selected as the feature genes. The first two principal component scores of the feature genes were extracted for hierarchical clustering using Euclidean distance and the Ward method. We separated samples into two groups using the “cutree” function. In general, one cluster contained tumor samples and the other contained normal samples. Any samples that were clustered outside of its general group label, e.g., tumor samples clustered within the normal sample cluster or normal samples in the tumor cluster, were filtered out (**Supplementary Note Figure 8, Supplementary Note Table 4**).

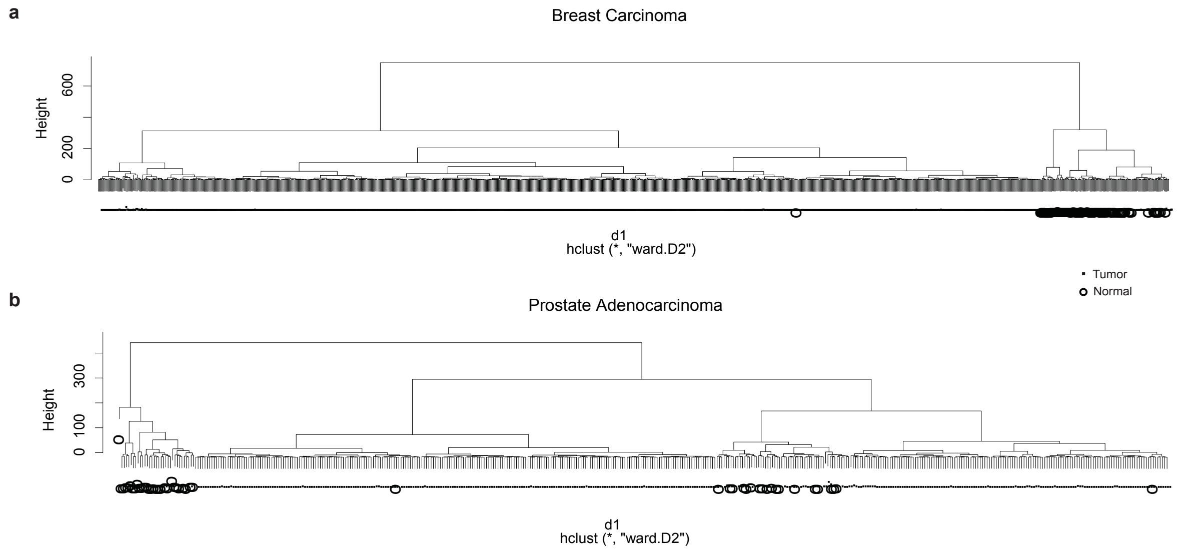

Supplementary Note Figure 8. Hierarchical clustering of read count data from adjacent normal and mixed tumor samples for breast and prostate adenocarcinoma in TCGA as examples. Dendrograms of the hierarchical clustering results based on the top 1,000 differentially expressed feature genes for breast carcinoma (**a**) and prostate adenocarcinoma (**b**), respectively. Circles represent adjacent normal samples and dots represent mixed tumor samples.

Supplementary Note Table 4. Summary of sample sizes for 15 TCGA cancer types.

Cancer type	Original number of normal samples	Original number of tumor samples	Number of normal samples after quality control	Number of tumor samples after quality control
Bladder urothelial carcinoma	19	401	17	385
Breast carcinoma	113	1074	98	1032
Colorectal carcinoma	51	633	43	598
Head & neck squamous cell carcinoma	44	495	31	494
Renal chromophobe	24	64	23	64
Renal clear cell carcinoma	72	513	66	495
Renal papillary carcinoma	32	277	26	276
Hepatocellular carcinoma	50	362	50	362
Lung adenocarcinoma	59	455	57	446
Lung squamous cell carcinoma	49	488	48	486
Pancreatic adenocarcinoma	4	150	7*	142
Prostate adenocarcinoma	52	406	47	295
Stomach adenocarcinoma	32	352	32	299
Thyroid papillary carcinoma	57	498	55	418
Endometrial carcinoma	35	526	26	524

*Pancreatic adenocarcinoma is the only cancer type with increased normal samples. This is due to the addition of pseudo-normal samples, which are tumor samples of stromal tissue with scant tumor presence.

Scale normalization at the seventy-fifth percentile based on the DSS package[50] was then applied to the post quality-control tumor and normal samples. Next, we applied two criteria to filter out spurious genes. First, we filtered out genes with a zero count in either the mixed tumor or normal samples. Second, we filtered out genes with a large variance ($\hat{\sigma}^2_{Ng} > 0.6$) in the normal reference samples. Here, the standard deviation of a gene is calculated as $\hat{\sigma}^2_{Ng} = sd(log_2(R_{.g}))$, where $R_{.g}$ is the normalized expression of gene g for normal reference samples.

For each cancer type, we applied the "DeMixT_DE" to the quality-controlled expression data together with simulated spike-ins as input data to generate initial tumor-specific mRNA proportions π_0. We used ASCAT estimated tumor purities as an informed prior to calculate a reasonable number for S_P. With other datasets in general, we set $S_P = max(50, 0.3*Sample\ size)$, as the default option of the "DeMixT_GS" function. Results from the TCGA datasets across 15 cancer types were largely consistent with small to moderate changes from the addition of spike-ins (**Supplementary Note Figure 9).**

We then used these $\pi'_0 s$ as initial values in the profile likelihood calculation on all genes to calculate gene selection scores. We ranked all genes based on their gene selection scores from smallest to largest. Based on a simulation study and observed distributions of gene selection scores in real data, we chose the top 1,500 or 2,500 genes to ensure accuracy in proportion estimation (**Supplementary Note Figure**

6a). Within each cancer type, we used the spike-ins as benchmarking samples and evaluated the RMSE of the estimated proportions of the spike-ins with either the top 1,500 or top 2,500 genes ($\hat{\pi}_{1500}(Sp)$ and $\hat{\pi}_{2500}(Sp)$). If RMSE($\hat{\pi}_{1500}(Sp)$-0)<RMSE($\hat{\pi}_{2500}(Sp)$-0), we used the results of the top 1,500 genes, *i.e.*, the tumor proportions $\pi=\pi_{1500}$; otherwise, $\pi=\pi_{2500}$. In general, the RMSEs were small (median = 0.02 across 15 cancer types), and the two sets of tumor proportions, $\hat{\pi}_{1500}$ and $\hat{\pi}_{2500}$, were consistent within each cancer type. We additionally removed samples with extreme estimates of π, >85% or ranked at the top 2.5 percentile of all samples within each cancer type, to mitigate the remaining underestimation when π is close to 1 and control the estimation bias in high values of TmS.

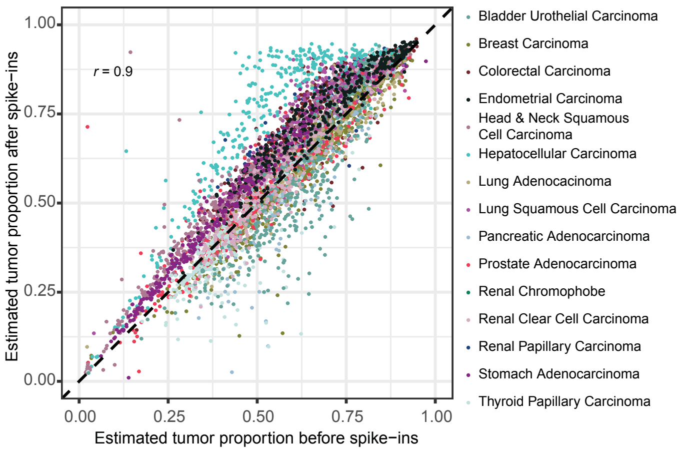

Supplementary Note Figure 9. Comparison of tumor-specific mRNA proportions with and without spike-ins across 15 TCGA cancer types. A scatter plot of estimated tumor-specific mRNA proportions using the "DeMixT_GS" method for 4,897 TCGA samples across 15 cancer types with and without spike-ins. The x axis represents the estimated tumor-specific mRNA proportions without spike-ins and the y-axis represents the estimated tumor-specific mRNA proportions with spike-ins.

2.3.2.2. Consensus TmS estimation

We first calculated TmS values for 5,295 TCGA samples with matched tumor-specific mRNA proportions and ABSOLUTE or ASCAT derived tumor purity and ploidy estimates. We then fitted a linear regression model on $\log_2$-transformed TmS calculated by ASCAT using $\log_2$-transformed TmS calculated by ABSOLUTE as a predictor variable. We removed samples with a Cook's distance ≥ 4/*n* (*n*=5,295) (**Extended Data Fig. 3f-h**), and for the remaining samples, which were the majority, we calculated the final TmS as: $TmS=\sqrt{TmS_{ASCAT} \times TmS_{ABSOLUTE}}$. These TmS estimates were used throughout the paper (**Supplementary Note Table 5**). A CONSORT diagram (**Supplementary Note Figure 10)** demonstrates the sample exclusion for TmS in TCGA**.**

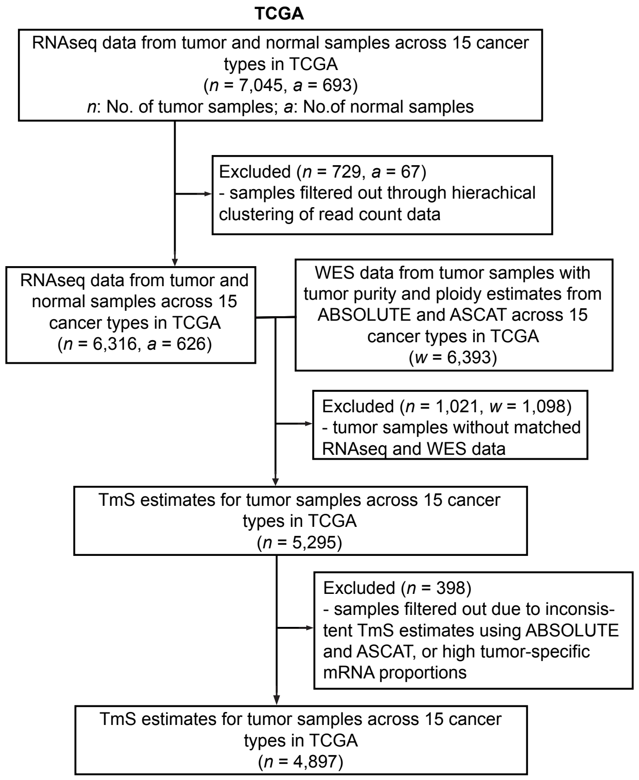

Supplementary Note Figure 10. CONSORT diagrams for data exclusions in TCGA.

Supplementary Note Table 5. Summary of sample sizes for 15 TCGA cancer types before and after consensus TmS estimation.

Cancer type	Number of samples before consensus analysis	Number of samples after consensus analysis	Number of samples removed
Bladder urothelial carcinoma	350	328	22
Breast carcinoma	932	916	16
Colorectal carcinoma	499	490	9
Head & neck squamous cell carcinoma	449	443	6
Renal chromophobe	59	59	0
Renal clear cell carcinoma	299	295	4
Renal papillary carcinoma	192	169	23
Hepatocellular carcinoma	333	317	16
Lung adenocarcinoma	399	395	4
Lung squamous cell carcinoma	440	431	9
Pancreatic adenocarcinoma	105	101	4
Prostate adenocarcinoma	266	259	7
Stomach adenocarcinoma	272	265	7
Thyroid papillary carcinoma	297	202	95
Endometrial carcinoma	403	361	42

2.3.2.3. Intrinsic tumor signature genes

For each cancer type, we selected the top 1,500 or 2,500 genes based on a gene selection score (Eq.S8), ranked from smallest to largest, as the intrinsic tumor signature gene (**Supplementary Note Figure 11a**). We found the genomic locations of the selected genes covered 22 autosomes and the X chromosome (**Supplementary Note Figure 11b**) across 15 cancer types, which is expected for an unbiased gene set to measure global gene expression. For each cancer type, as well as consistently across 15 cancer types, we find that 54-68% (mean = 62%) of intrinsic tumor signature genes are housekeeping genes[51] or essential genes[52], and 3.5-7.2% (mean = 5.4%) are *MYC* targets genes (**Supplementary Note Figure 11c**). The common pan-cancer essential genes are derived from a total of 147 cancer cell lines and 16,733 genes that were screened independently by both the Sanger and Broad institutes[52].

We conducted gene set enrichment analyses on Hallmark pathways and KEGG pathways[53] using GSEA[54] and g:Profiler[55]. For each cancer type, the genes were ranked according to their gene selection scores from the smallest to the largest. For GSEA, we adopted permutation tests (1,000 times) to generate a normalized enrichment score (NES), the null distribution and an adjusted *P* value for each candidate pathway[54]. g:Profiler detects statistically significantly enriched pathways for the given gene list by implementing hypergeometric tests. For each candidate pathway a nominal *P* value is calculated by

the hypergeometric test and adjusted for multiple testing by the BH method. As a result, the minimum NES of significantly enriched Hallmark pathways and KEGG pathways are above 1.74 and 1.70, respectively.

We further evaluated the chromatin accessibility of signature genes using ATAC-seq data TCGA samples[33]. For each sample, peak scores (-log10(p-value)) were scaled by dividing each individual peak score by the sum of all of the peak scores in the given sample divided by 1 million. These scaling values ranged from 1.4 to 67.4 across cancer types. The 75th percentile of normalized peak scores across all peaks within the promoter region were selected for each gene as representative peak scores, and genes with normalized peak scores less than 1 were excluded. A total of 7.1% to 20.4% of genes were excluded across cancer types (**Supplementary Note Figure 11d**). For each sample, we calculated the mean of

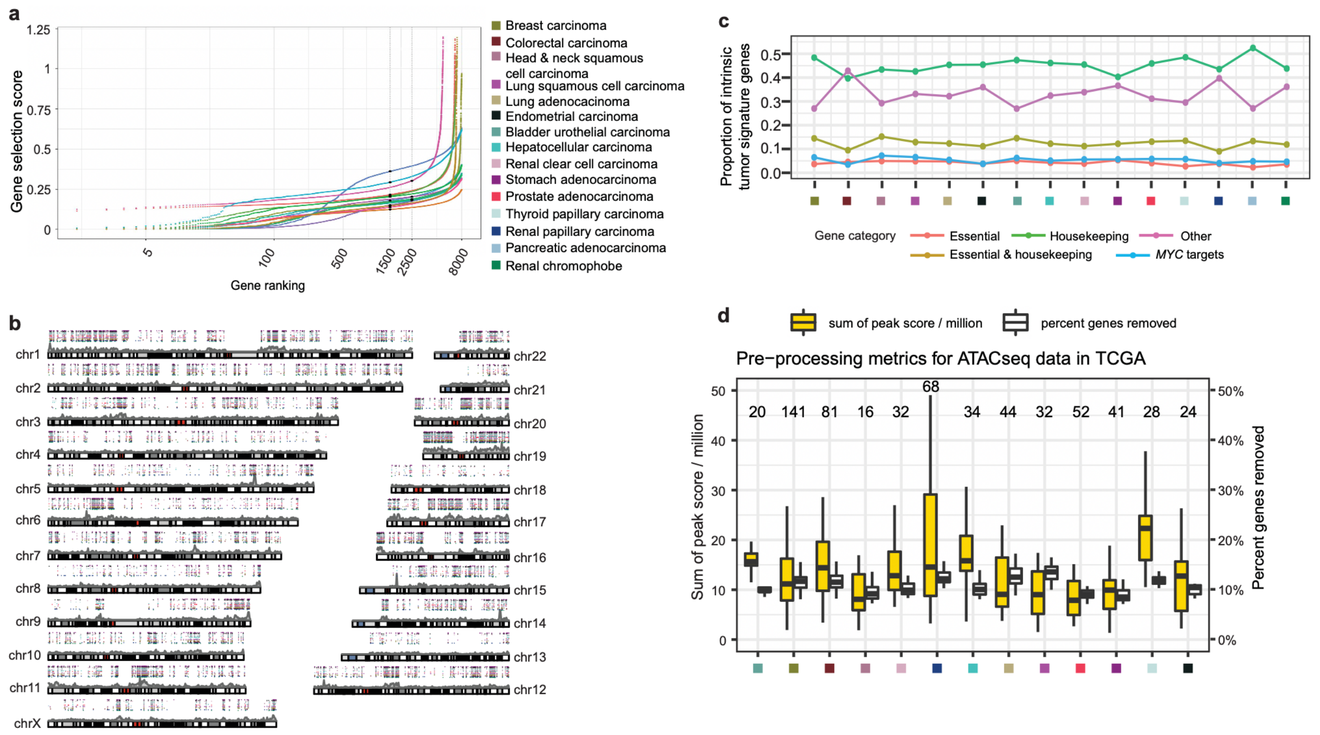

Supplementary Note Figure 11. Validation of gene selection to represent intrinsic tumor signature across cancer types. a, Ordered gene selection scores of all genes from low to high across 15 cancer types. The black solid dots represent the cutoffs for gene selection in the corresponding cancer type. **b**, Karyotype plots showing the genomic locations of signature genes for each cancer type. Signature genes are presented as dots colored by cancer type. An overall gene density track is shown in gray shades underneath the dots. The density of signature genes is consistent with the overall gene density. **c**, Proportions of the intrinsic tumor signature gene categories across 15 cancer types. **d**, Boxplots showing sums of peak scores across samples / 1 million (yellow) and percentages of genes removed per sample (white). In the boxplots, whiskers represent the maximum and minimum values, the middle line in the box denotes median and the bounds of the box stand for upper and lower quartiles. The sums of peak scores were used as scaling factors to normalize the ATAC-seq data. The genes with normalized scores <1 were removed from downstream analysis. For a,b,c,e, all 15 TCGA cancer types are annotated using colored squares as shown in the legend of a. For each cancer type, the number of samples used is indicated on the top.

the peak scores of all signature genes. A null distribution of mean peak scores was generated by calculating means from 1,000 random subsets of genes with the matching number of the signature genes from all genes. *P* values for signature genes were calculated as the percentile of the permuted means being greater than or equal to the observed mean. *P* values were adjusted for multiple testing by the BH method.

2.3.2.4 Association of TmS with genetic alterations

For each cancer type within TCGA, we searched among driver mutations (including nonsense, missense and splice-site SNVs and indels)[32] over all genes for the 15 cancer types to identify those that were significantly associated with TmS. For each cancer type, we considered genes which had driver mutations in at least 10 samples. For each of these genes, samples were labelled as “with driver mutation” if they carried at least one driver mutation in that gene or “without driver mutation” otherwise. We investigated 24 cancer-gene pairs for the driver mutation analysis. We applied a Wilcoxon rank-sum test to each candidate gene to compare the distributions of TmS of the samples with driver mutations versus without driver mutations. The *P* values of each gene were adjusted for multiple testing using BH correction across all candidate genes within the corresponding cancer type.

We also implemented an agnostic search among non-synonymous mutations (including SNVs and indels) over all genes for the 15 cancer types to identify those that were significantly associated with TmS. For each cancer type, we considered a gene as a candidate gene if there were at least 10 samples containing non-synonymous mutations in that gene. We investigated 32,894 cancer-gene pairs for non-synonymous mutation analysis. We applied two statistical tests to evaluate the difference between the “with non-synonymous mutation” and “without non-synonymous mutation” samples. We first applied a Wilcoxon rank-sum test for each candidate gene to evaluate the difference between the distribution of TmS of the two group of samples. We then fitted a linear regression model using $\log_2$-transformed TmS as the dependent variable and mutation status as a predictor: $log_2(TmS)=b_0+b_1\,log_2(TMB)+b_2MUT$, where TMB represents tumor mutation burden. $MUT=1$ if the sample has at least one non-synonymous mutation in the candidate gene, and $MUT=0$ otherwise. The *P* values were calculated by a t-test of the regression coefficient b_2. The *P* values of each gene based on Wilcoxon rank-sum test and t-test were adjusted by BH correction based on the number of candidate genes within the corresponding cancer type.

We find 5 overlapping pairs out of 6 statistically significant pairs produced from each interrogation (adjusted *P* values < 0.01). The same significant associations with *PIK3CA* and *TP53* mutations in TmS were found in the TCGA breast cancer study (adjusted *P* values <0.001). The additional pair found through the agnostic search (*FGFR3* in bladder carcinoma in TCGA) was not identified in the driver mutation analysis due to a limited sample size. These associations in breast, lung, thyroid, and bladder

cancers show that TmS can capture changes in tumor phenotypes induced by driver mutations in a cancer type-specific manner[32]. This is consistent with previous findings that the same driver mutations may not have the same prognostic effect across cancers[56,57], and their effects are modified by additional tumor and/or treatment-related factors. In this context, our results indicate that TmS can be used to prognostically stratify tumors beyond driver mutation status.

Tumor mutation burden (TMB) was calculated by counting the total number of all somatic mutations based on the consensus mutations calls (MC3)[58]. Chromosomal Instability (CIN) scores were calculated as the ploidy-adjusted percent of genome with an aberrant copy number state. ASCAT was used to calculate allele-specific copy numbers[21]. For samples present in both TCGA and PCAWG, the consensus copy number was derived from published results[59]. We calculated the Spearman correlation coefficients between TmS and /TMB/CIN scores for each cancer type in TCGA (**Supplementary Note Table 6**).

Supplementary Note Table 6. Spearman correlation coefficients between TmS and TMB/CIN scores across 15 TCGA cancer types.

Cancer type	TMB	CIN
Breast carcinoma	0.35	0.46
Lung adenocarcinoma	0.3	0.23
Thyroid papillary carcinoma	0.066	0.1
Pancreatic adenocarcinoma	0.082	0.1
Renal clear cell carcinoma	0.17	0.35
Lung squamous cell carcinoma	0.04	0.051
Bladder urothelial carcinoma	0.21	0.24
Renal papillary carcinoma	-0.21	0.18
Colorectal carcinoma	-0.021	0.056
Prostate adenocarcinoma	0.072	0.3
Endometrial carcinoma	-0.062	0.16
Hepatocellular carcinoma	-0.066	-0.095
Head & neck squamous cell carcinoma (HPV+)	0.32	0.27
Head & neck squamous cell carcinoma (HPV-)	-0.017	-0.081
Stomach adenocarcinoma	-0.073	-0.15
Renal Chromophobe	0.13	0.23

2.3.2.5 Association of TmS with expressions of pluripotency and proliferation genes, and patient characteristics

We found that TmS is mostly uncorrelated with the expression levels of canonical pluripotency genes *SOX2*, *MYC*, *KLF4* and *POU5F1*[60] in bulk tissue samples (**Supplementary Note Figure 12**). The

corresponding correlation coefficients are much lower than those of TmS with the proliferation marker genes (*MKI67* and *PCNA*) (**Supplementary Note Figure 12**). This observation does not necessarily rule out any close relationship between these genes and tumor-cell transcriptome variations, as their expression levels from non-tumor cells, which could be major confounders, were also profiled in the bulk samples.

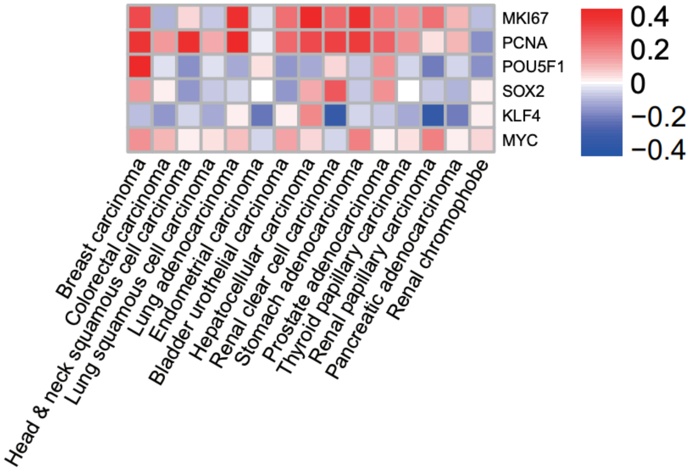

Supplementary Note Figure 12. Spearman correlation coefficients between TmS and the expression levels of *MKI67* and *PCNA* and pluripotency genes across 15 cancer types.

We also observed that TmS is unassociated with clinical characteristics of patients, including age and sex, across the 15 TCGA cancer types (**Supplementary Note Figure 13a**). We investigated the correlation between TmS and the Tumor-Node-Metastasis (TNM) stage which was dichotomized into early and advanced groups. Since prostate adenocarcinoma, endometrial carcinoma and renal chromophobe did not have TNM stage information, and pancreatic adenocarcinoma had very imbalanced sample distribution in early (*n*=91) *vs.* advanced (*n*=4) stages, they were removed from this analysis. In 4 (stomach adenocarcinoma, bladder urothelial carcinoma, renal clear cell carcinoma and renal papillary carcinoma) of the remaining 11 cancer types from TCGA, high TmS is associated with advanced stage; no correlation between TmS and TNM stage was observed in other cancer types (**Supplementary Note Figure 13b**).

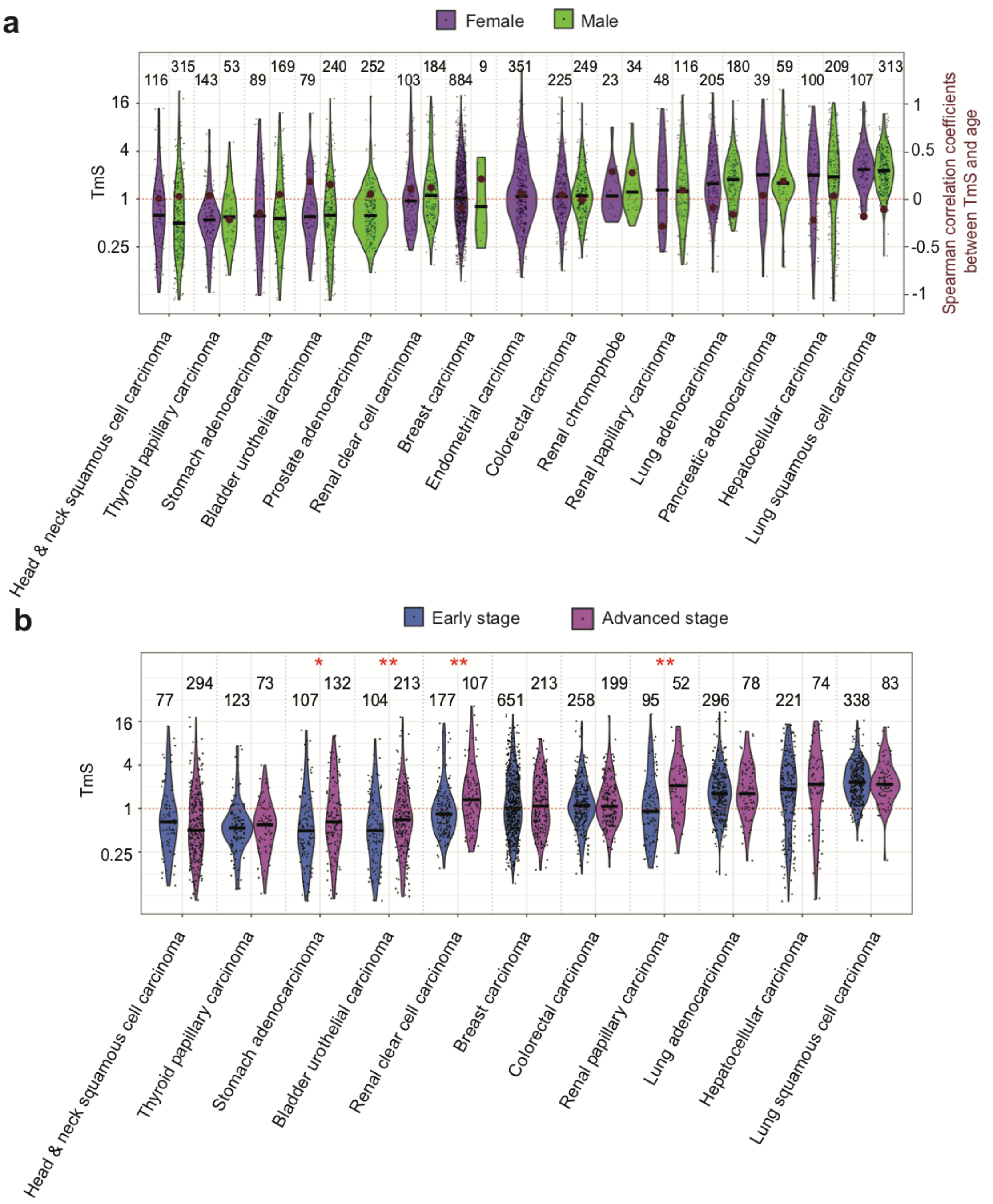

Supplementary Note Figure 13. Associations between TmS and patient characteristics. a, Distribution of TmS for female and male patient samples in TCGA across 15 cancer types. None of the adjusted *P* values of two-sided Wilcoxon rank-sum tests comparing TmS between the two groups reached significance at a confidence level of 0.05. Brown circles (read out on the right y-axis) represent Spearman correlation coefficients between TmS and age within the same sex and cancer type. The red dotted horizontal line represents TmS equal to 1 (left y axis) and correlation equal to 0 (right y axis). None of the adjusted *P* values for correlation tests reached significance at a confidence level of 0.05. **b,** Distribution of TmS for early (stage I and II) *vs*. advanced (stage III and IV) pathological stages across 15 cancer types. BH adjusted *P* values of two-sided Wilcoxon rank-sum tests are indicated by asterisks (**P* < 0.05, ** < 0.01, *** < 0.001).

2.3.3. ICGC-EOPC

For this dataset, DNAseq-based purity and ploidy estimates for 113 samples from 89 patients were determined by Sequenza[61]. We used the 9 available adjacent normal samples as the normal reference to run DeMixT. The RNAseq data came from three batches - batch 1 (17 patients, 25 samples), batch 2 (42 patients, 52 samples), and batch 3 (37 patients, 44 samples). We have conducted a comprehensive comparison for deconvolution with and without batch effect correction, and concluded that we will report both TmS values estimated with and without batch effect correction (see details in **Supplementary Note 3.1.1**). A CONSORT diagram is provided for this dataset to demonstrate the sample filtering steps (**Supplementary Note Figure 14**).

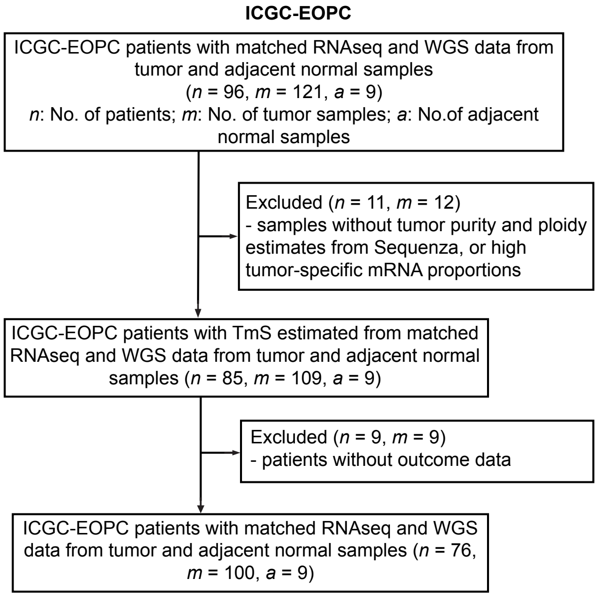

Supplementary Note Figure 14. CONSORT diagram for data exclusions in ICGC-EOPC.

2.3.4. METABRIC

Tumor purity and ploidy for each of the 1,992 patient samples was estimated using both ASCAT[14] and Sequenza[61] based on the LogR and B Allele Frequency (BAF) data processed from the Affymetrix CEL files by the PennCNV library[62]. For each expression array, we first annotated the probes using the Illumina HumanHT12v3 annotation data, and only kept 29,438 probes that have matched gene symbols for downstream analyses. We applied the DeMixT deconvolution pipeline to the expression arrays of the combined discovery and validation sets, after batch effect correction by limma[63], to estimate tumor-specific proportions using the adjacent normal samples as the reference. The consensus TmS strategy was applied to obtain robust TmS estimations. 1,664 patient samples with TmS remained after the above steps. We additionally removed 118 patient samples due to missing follow-up information of biochemical

recurrence intervals or the PAM50 subtypes. A final cohort of 1,546 patient samples from both the discovery and validation sets were kept for downstream analyses. A CONSORT diagram is provided for this dataset to demonstrate the sample filtering steps (**Supplementary Note Figure 15**). See details in batch effect correction in **Supplementary Note 3.1.1.**

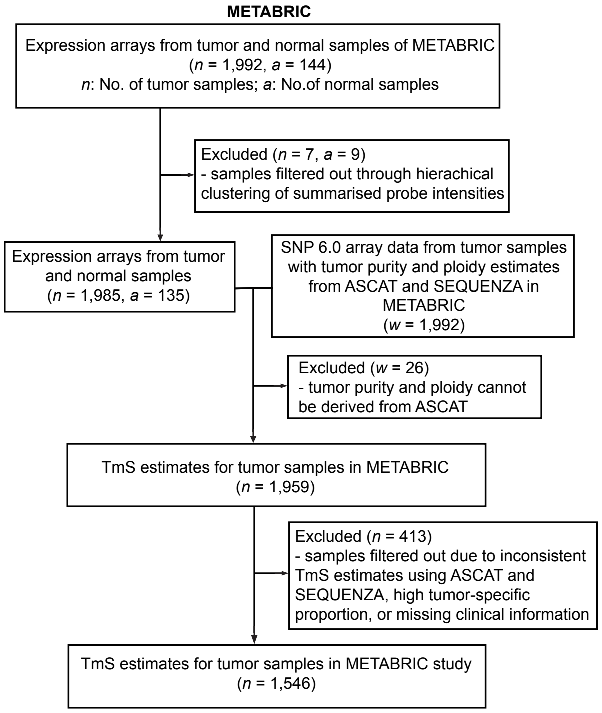

Supplementary Note Figure 15. CONSORT diagram for data exclusions in METABRIC.

2.3.5. TRACERx

This dataset does not contain RNAseq data from adjacent normal samples, which is required for running DeMixT. Instead, we used RNAseq data from normal lung samples which are available in the GTEx[64] study. To mitigate the technical artefacts, such as batch effects, scale normalization was applied before deconvolution. The tumor-specific mRNA expression proportions for 159 tumor samples from 64 patients were estimated using the “DeMixT_DE” mode with the top 1,500 genes[20]. The other 168 tumor samples from 36 patients with only DNAseq data and no matching RNAseq data were removed. DNA-based tumor purity and ploidy were estimated by Sequenza[61]. In the end, we focus on 30 patients (19 with lung adenocarcinoma and 11 with lung squamous cell carcinoma) with multi-region samples (m = 94; m denotes the number of tumor samples) and 52 patents with both single and multi-region samples (m = 116) for the downstream analysis. A CONSORT diagram (**Supplementary Note Figure 16)** demonstrates the sample exclusion for TmS in TRACERx**.**

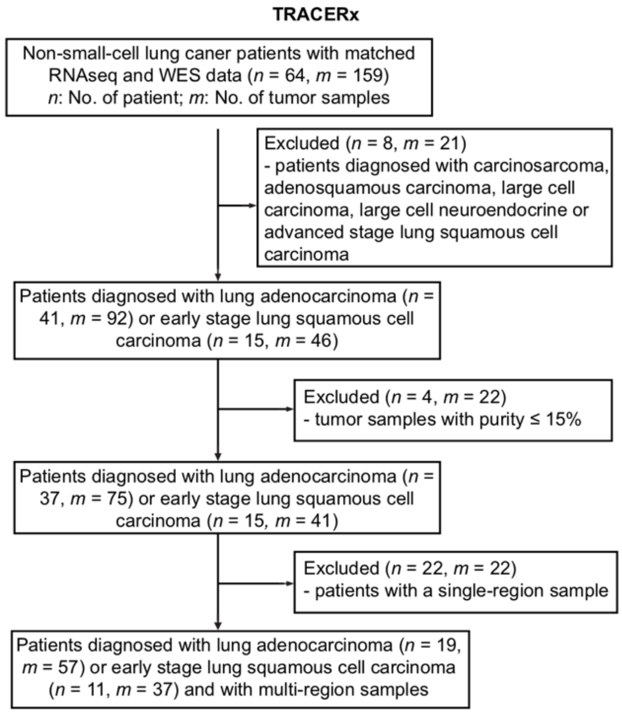

Supplementary Note Figure 16. CONSORT diagram for data exclusions in TRACERx.

Deconvolution using normal reference samples from GTEx

We conducted a series of experiments across cancer types to evaluate the impact of technical artefacts such as batch effects to the proportion estimation when using a different cohort. We first applied GTEx expression data[64] from normal prostate samples as the normal reference to deconvolute the TCGA prostate cancer samples. Even though the overall performance of deconvolution was negatively impacted, the estimated proportions showed a reasonable correlation (Spearman correlation coefficient = 0.65) with those generated using TCGA normal prostate samples as the normal reference (**Supplementary Note Figure 17a**). We repeated this experiment on the deconvolution of TCGA thyroid papillary carcinoma samples using RNAseq data from TCGA normal and GTEx normal thyroid samples as the reference, respectively. Again, the two sets of estimated tumor-specific mRNA expression proportions were highly correlated (Spearman correlation coefficient = 0.65) (**Supplementary Note Figure 17b**). For the EOPC tumor samples where RNAseq data from 9 normal samples were available, we observed a higher correlation (Spearman correlation coefficient = 0.77) between the estimated tumor-specific mRNA expression proportions using EOPC normal and TCGA prostate normal samples on the deconvolution of

EOPC tumor samples, respectively (**Supplementary Note Figure 17c**). Furthermore, for the deconvolution of TRACERx tumor samples, we also observed a high correlation (Spearman correlation coefficient = 0.83) between the estimated tumor proportions using TCGA and GTEx normal lung samples as the reference, respectively. (**Supplementary Note Figure 17d**). We calculated TmS values for all regions (median number of regions per patient = 2, ranging from 1 to 6) in the TRACERx dataset.

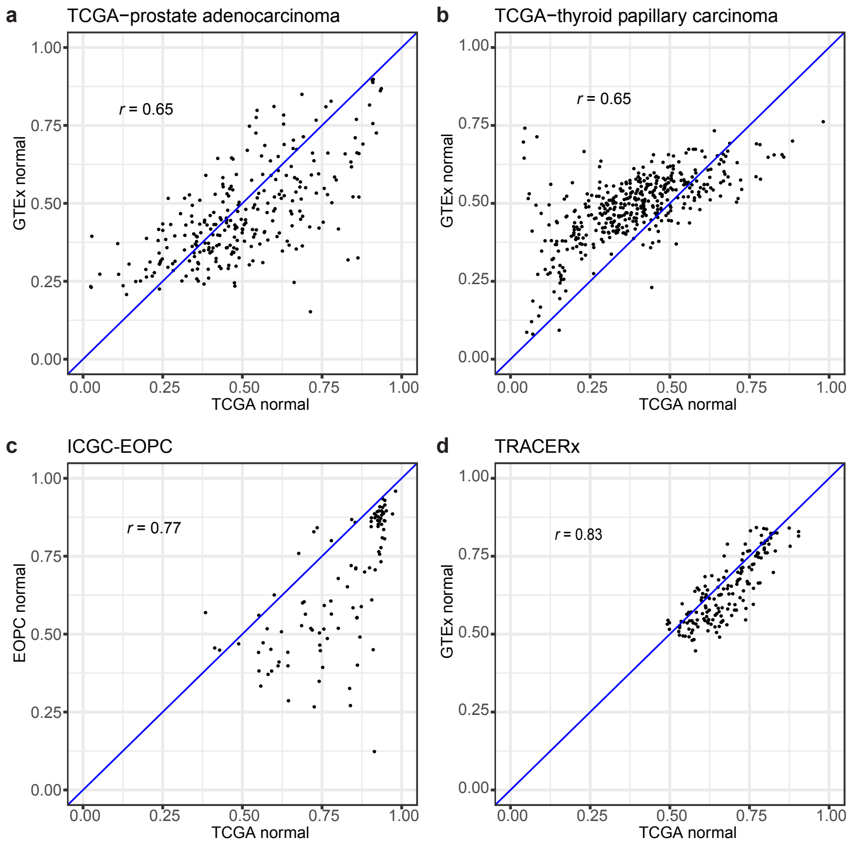

Supplementary Note Figure 17. DeMixT deconvolution using normal reference from different studies. a, Scatter plot of DeMixT estimated tumor proportions for TCGA-prostate adenocarcinoma samples using GTEx normal (y axis) or TCGA normal (x axis) samples. **b**, Scatter plot of DeMixT estimated tumor proportions of EOPC using EOPC normal (y axis) and TCGA normal (x axis) samples. **c**, Scatter plot of DeMixT estimated tumor proportions of TCGA-thyroid papillary carcinoma samples using GTEx normal (y axis) and TCGA normal (x axis) samples. **d**, Scatter plot of DeMixT estimated tumor proportion of TRACERx samples using GTEx normal (y axis) and TCGA normal (x axis) samples. Spearman correlation coefficients (*r*) between the two sets of tumor proportion estimates are shown on the top of each panel.

3. STATISTICAL ANALYSIS

3.1. Robustness of TmS

3.1.1. Batch effect correction

For RNAseq data from multiple batches, we applied batch effect correction using ComBat[65] and limma to combine RNAseq data in one pool before estimating tumor-specific mRNA proportions. The TmS estimates after the batch effect correction were used in the downstream analyses.

For the ICGC-EOPC dataset, the RNAseq data came from three batches - batch 1 (17 patients, 25 samples), batch 2 (42 patients, 52 samples), and batch 3 (37 patients, 44 samples). We first evaluated batch effects using PCA. We observed a moderate batch effect across the three batches (**Supplementary Note Figure 18a**) which was removed after correction using Combat (**Supplementary Note Figure 18b**). To evaluate the impact of batch effects, we applied the DeMixT deconvolution pipeline

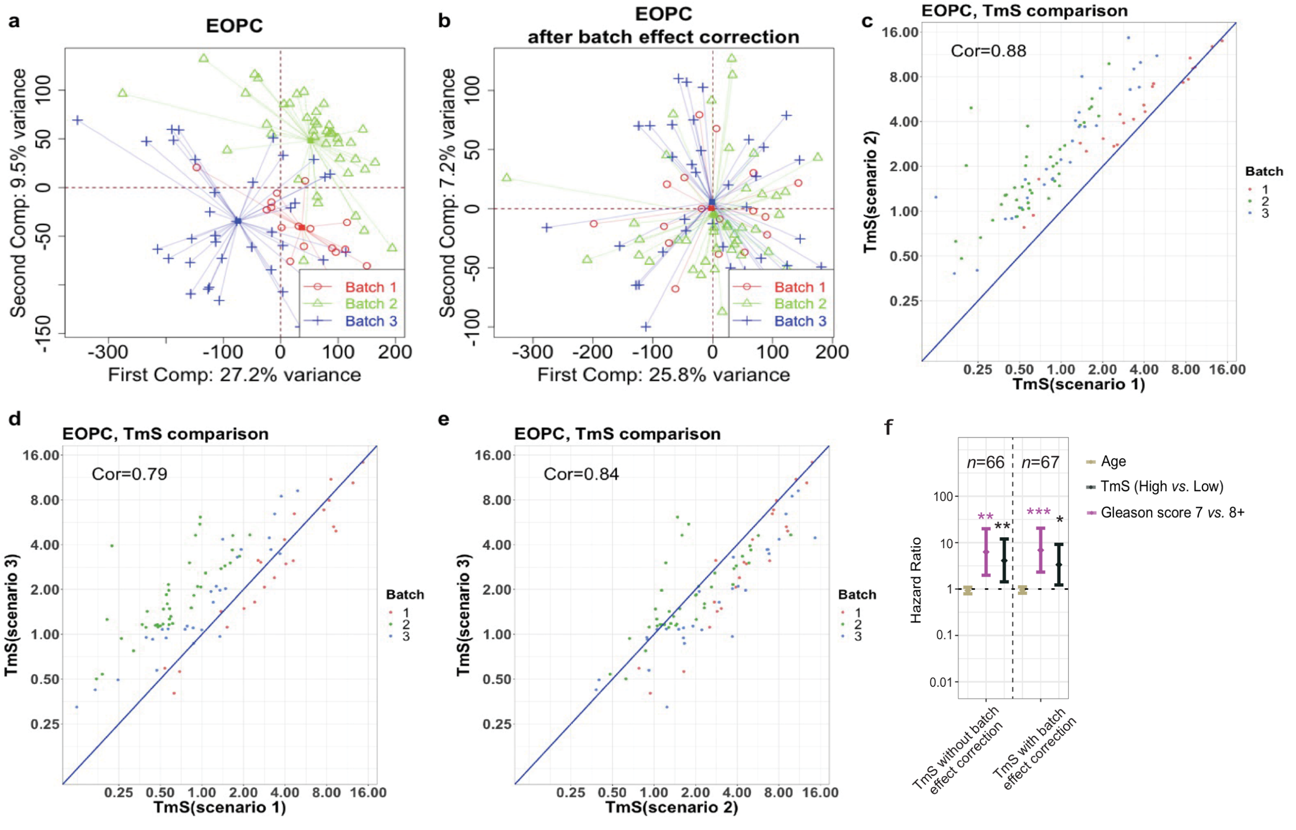

Supplementary Note Figure 18. a, PC1 *vs*. PC2 of the EOPC samples from 3 batches before batch effect correction. **b**, PC1 *vs*. PC2 of EOPC samples from 3 batches after batch effect correction. **c**, A scatter plot of estimated TmS under scenario (1) *vs*. scenario (2). **d**, A scatter plot of estimated TmS under scenario (1) *vs*. scenario (3). **e**, A scatter plot of estimated TmS under scenario (2) *vs*. scenario (3). The Spearman correlation coefficients between the TmS estimates was shown in each figure. **f**, Forest plot of hazard ratios (center points) and 95% CIs (error bars) of multivariate Cox proportional hazard models with Age, TmS and Gleason score as predictors for PFI in EOPC.

in three scenarios: (1) all samples together; (2) each batch separately; (3) all samples together using batch effect corrected data. The Spearman correlation coefficients between TmS obtained in pairwise comparisons were high: 0.88, 0.79, and 0.84 (**Supplementary Note Figure 18c-e**). In addition, the survival analysis using the TmS with batch effect correction are consistent and robust comparing to those without correction (**Supplementary Note Figure 18f**). Any further data manipulation could potentially introduce unwanted variations. We made further observation that TmS values without correction are closer to those from the TCGA-PRAD data. We therefore chose TmS estimates without batch effect correction as our main output.

For the METABRIC dataset, the microarray data came from two batches, i.e., the discovery set and the validation set. We observed a significant batch effect between the two batches (**Supplementary Note Figure 19a**). The batch effect was removed using both limma and ComBat (both show consistent results) (**Supplementary Note Figure 19b**). To demonstrate the impact of batch effects, we applied the DeMixT deconvolution pipeline in the following scenarios: (1) each batch separately; (2) all samples together using batch effect corrected expression data. The TmS estimates under the two scenarios are highly correlated (Spearman *r*=0.95, **Supplementary Note Figure 19c**). In summary, TmS estimates were consistent before and after batch effect correction.

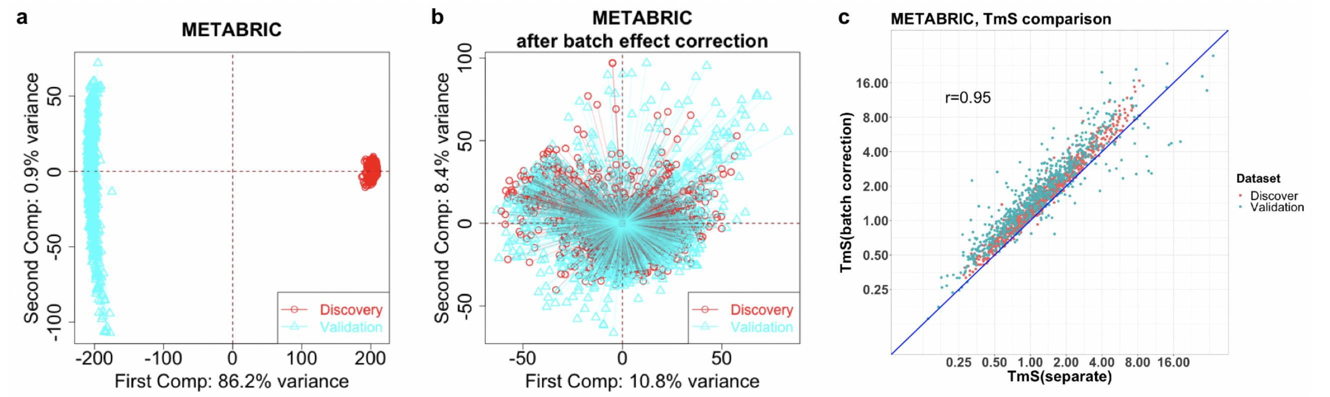

Supplementary Note Figure 19. a, PC1 *vs*. PC2 of METABRIC samples from discovery and validation cohorts without batch effect correction **b,** PC1 *vs*. PC2 of METABRIC samples after batch effect correction. **c**, A scatter plot of estimated TmS after batch effect correction (y axis) *vs*. estimated TmS for discovery and validation cohort separately (x axis).

Furthermore, we found TmS results were consistent across technological platforms, e.g., in breast cancer between the microarray-based METABRIC data and the TCGA RNAseq data, across PAM50 subtypes (**Supplementary Note Figure 20a**). Q-Q plots showed similar distributions of TmS between the two platforms for the Luminal B, Her2 and Basal subtypes (**Supplementary Note Figure 20b**). There is an elevation of TmS in the METABRIC-LumA subtype, which may be explained by a discrepancy in the clinical ascertainment of the study cohorts, potential mistakes in annotating ER/PR status, or some unknown technical effects.

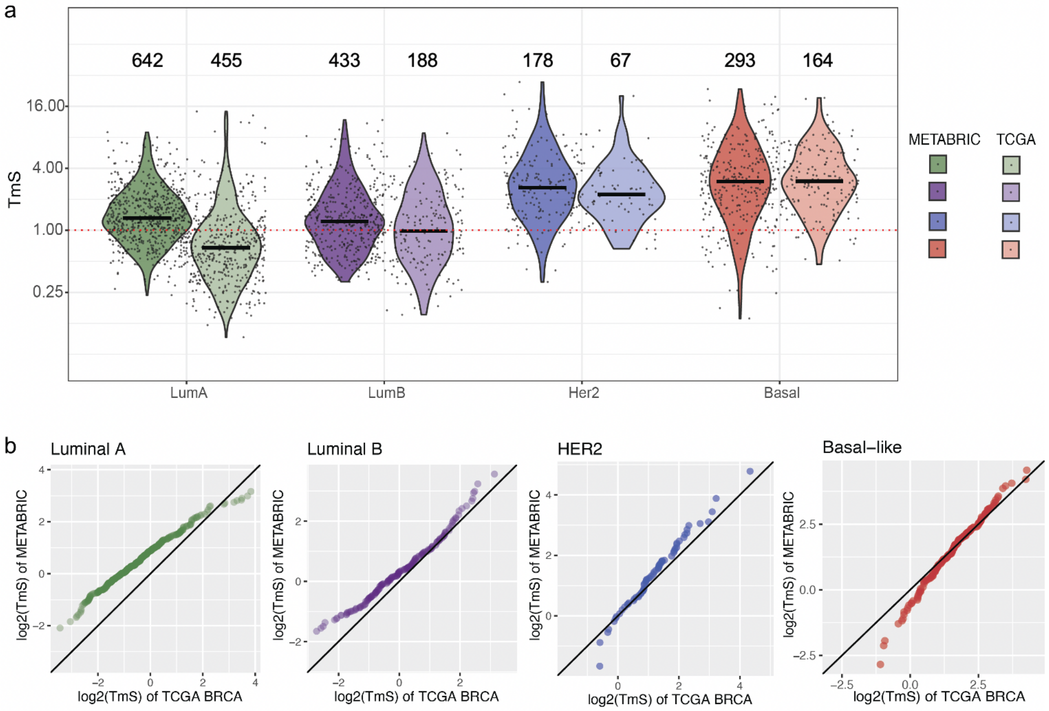

Supplementary Note Figure 20. TmS for each PAM50 subtype across TCGA and METABRIC. a, Distributions of TmS across PAM50 subtypes in TCGA BRCA and METABRIC. Numbers on top represent sample sizes. **b**, Q-Q plots for TmS in TCGA versus METABRIC for each PAM50 subtype.

3.1.2. Adjustment for focal copy number alterations

TmS may potentially be influenced by the boost in RNA content arising from focal amplifications and loss of RNA content from focal deletions. To evaluate this, we re-estimated TmS of 4,897 tumor samples across 15 cancer types in TCGA after excluding the expression data from genes with focal amplifications and homozygous deletions in the estimation of TmS. First, we identified all focal amplifications and homozygous deletion events in each tumor sample using the tumor-specific copy number profiles estimated by ASCAT (**Supplementary Note Figure 21**). We define a focal event as a CNA smaller than 10MB. For samples without or with a whole genome duplication event, a gene is defined as amplified if the tumor-specific copy number is greater than or equal to 5 or 9, respectively. For genes with amplification or homozygous deletion events occurring in more than 10 samples, we removed their corresponding expression data from all samples. For genes presenting focal CNA events occurring in less than 10 samples, we replaced their expression data in these samples with the median expression from all samples. After making these adjustments, we re-estimated TmS from the TCGA datasets across 15 cancer types and found that they were consistent with original TmS estimations reported in the manuscript (the median of the difference in TmS estimates is 0.07, the median absolute deviation of the difference in TmS estimates is 0.2, also see **Supplementary Note Figure 22**).

Using the re-estimated TmS with adjustment to focal CNAs, we fitted multivariate Cox models with Age, Stage and TmS as the baseline model, and the interaction term between TmS and Stage (TmS x Stage), as the candidate predictor for the response variable of overall survival (OS) or progression free interval (PFI). The results from the re-estimated TmS are again consistent with those obtained from the original TmS (**Supplementary Note Figure 23**).

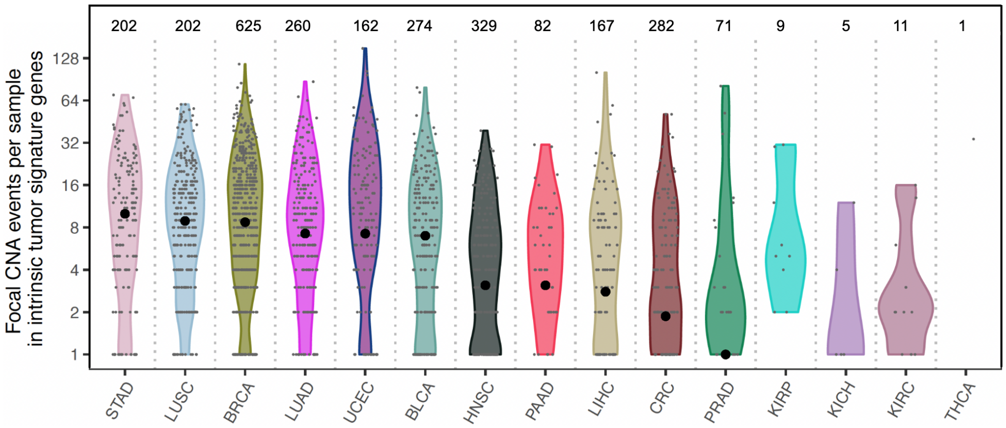

Supplementary Note Figure 21. Distribution of focal CNA events per sample in intrinsic tumor signature genes across 15 cancer types in TCGA. Large points represent mean focal CNA events per sample in the corresponding cancer type. For KIRP, KICH, KIRC, and THCA, the mean values of focal CNA events per sample are smaller than 1 (not shown in figure).

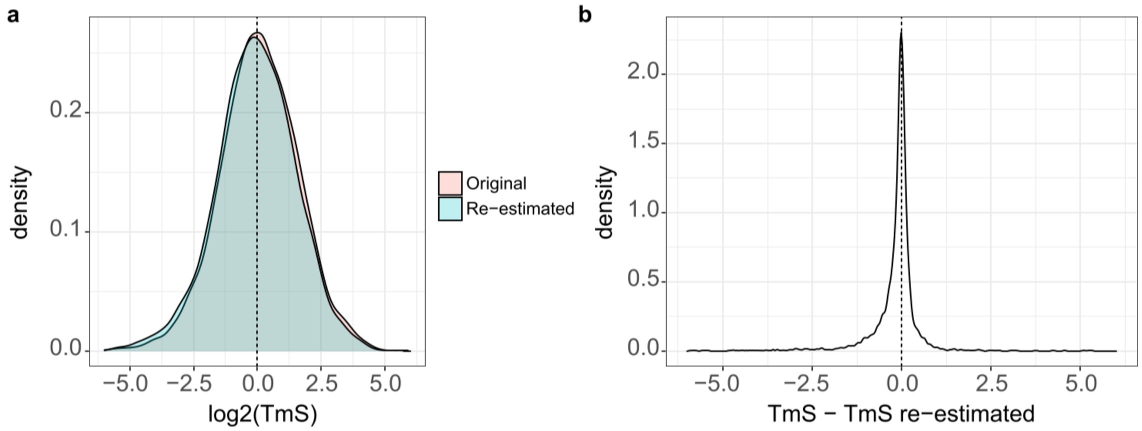

Supplementary Note Figure 22. Density plots of the original and the re-estimated TmS (**a**) and their differences (**b**).

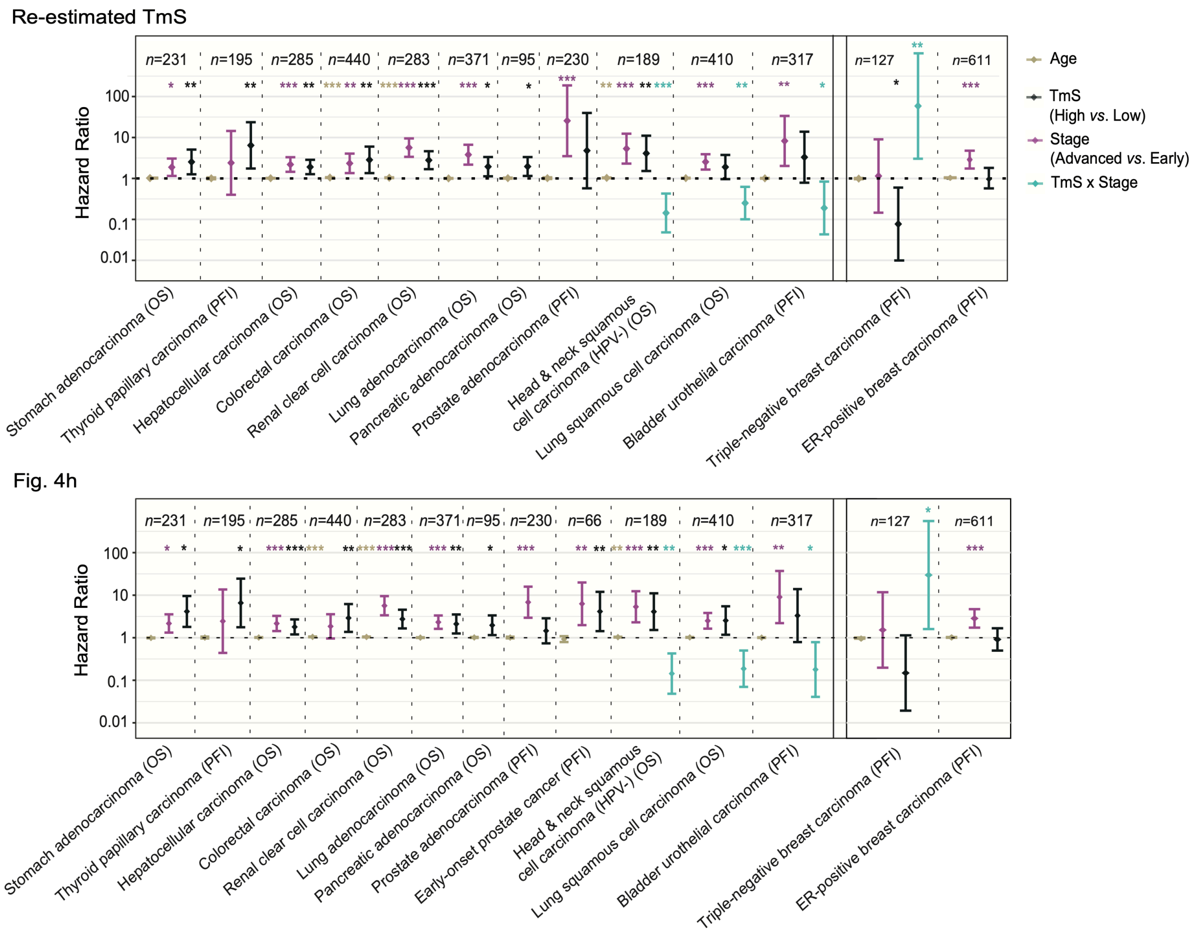

Supplementary Note Figure 23. Forest plots of hazard ratios (center point) and 95% of CIs (error bar) of multivariate Cox proportional hazard models using re-estimated TmS in comparison with **Fig. 4h**. *P* values of two-sided Wald tests for the covariates are indicated by asterisks (* $P < 0.05$, ** < 0.01, *** < 0.001). For each cancer type, the number of samples is indicated on the top.

3.2. Survival analysis

3.2.1. Association analysis of TmS in survival outcomes

For the TCGA datasets, we used clinical data that passed at least one of the three quality control steps introduced from the TCGA pan-cancer clinical paper[66]. We used two survival outcomes, the OS and PFI. To ensure sufficient sample size in each category, we combined the pathologic stages into two categories: early stage and advanced stage. The early stage includes Stage I, Stage IA, Stage IB, Stage IC, Stage II, Stage IIA, Stage IIB, and Stage IIC, while the advanced stage consists of Stage III, Stage IIIA, Stage IIIB, Stage IIIC, Stage IV, Stage IVA, Stage IVB, and Stage IVC. With prostate cancer, we used Gleason score (Gleason Score = 7 versus Gleason Score ≥ 8) instead of early and advanced stage. The

CONSORT diagram that demonstrates the sample exclusion for survival analysis in TCGA is shown in **Supplementary Note Figure 24.**

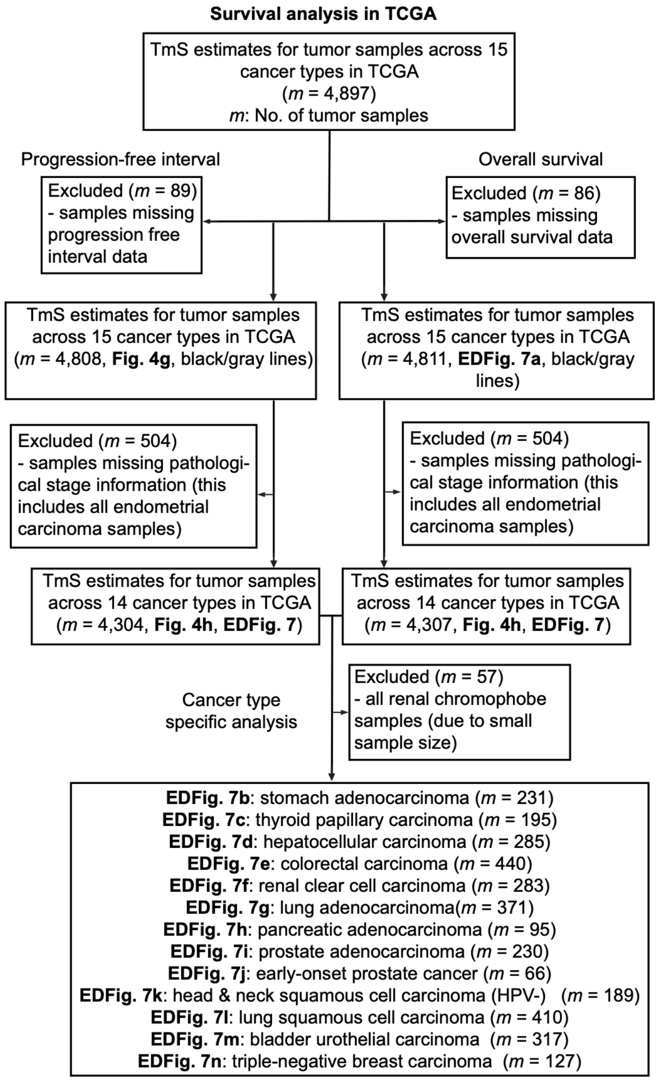

Supplementary Note Figure 24. CONSORT diagram for data exclusions in TCGA survival analysis.

Due to the potential nonlinear relationship between TmS and survival outcomes, we used a recursive partitioning survival tree model, rpart[67], to find an optimized TmS cutoff that best differentiates survival outcomes within each of the two stages as defined above in each cancer type. The splitting criteria were Gini index, and the maximum tree depth was set to 2. The TmS cutoffs of early/advanced stage across cancers are shown in **Supplementary Note Table 7**.

Supplementary Note Table 7. Summary of TmS cutoffs for early/advanced stage across cancers in TCGA and ICGC (a), breast cancers in METABRIC (b) and lung cancers in TRACERx (c).

a. TCGA and ICGC

Cancer type	Overall survival		Progression-free interval	
	Early stage	Advanced stage	Early stage (Gleason score = 7 for prostate cancers)	Advanced stage (Gleason score ≥ 8 for prostate cancers)
Pan-Cancer (14 cancer types)	1.10	1.72	1.65	1.72
Bladder urothelial carcinoma	0.15	NA	0.15	0.60
Triple-negative breast carcinoma	4.11	1.80	3.02	2.97
Colorectal carcinoma	1.94	4.52	NA	4.14
Head & neck squamous cell carcinoma (HPV-)	1.00	0.26	0.14	0.26
Renal clear cell carcinoma	0.54	1.78	0.33	1.67
Hepatocellular carcinoma	0.16	1.81	NA	0.64
Lung adenocarcinoma	0.81	0.97	0.51	8.66
Lung squamous cell carcinoma	6.67	2.08	5.67	6.37
Pancreatic adenocarcinoma	1.83	NA	1.83	NA
Stomach adenocarcinoma	0.40	0.15	0.28	0.31
Thyroid papillary carcinoma	NA	NA	0.57	1.25
Prostate adenocarcinoma	NA	NA	0.50	0.48
Early-onset prostate cancer (ICGC-EOPC)	NA	NA	1.25	0.84

b. METABRIC

Breast carcinoma subtype	Disease-free survival
Triple-negative breast carcinoma	1.73
Triple-negative breast carcinoma treated with chemotherapy	1.73
ER-positive breast carcinoma	1.30
ER-positive breast carcinoma treated with chemotherapy	1.30

c. TRACERx (TmS_{max})

Cancer type	Disease-free survival
Lung adenocarcinoma and lung squamous cell carcinoma	3.48

We have evaluated the predictive power of different dichotomizations of TmS (rpart, median, lower tertile, upper tertile) as well as the continuous form of $\log_2$(TmS) for survival outcomes. We fitted multivariate Cox proportional hazard models with Age, TmS (High vs. Low) and Stage (Advanced vs. Early) as predictors, and with overall survival (OS) or progression-free interval (PFI) as response variable across five cancer types (**Supplementary Note Figure 25**, for simplification, only the hazard ratios of TmS are shown). Endometrial cancer was excluded in the main analysis due to the lack of stage information, although we also performed Cox regression without Stage for this cancer type (**Supplementary Table 5**). Using the criteria that the number of samples within the early and the advanced-stage groups respectively should be greater than 30 and the number of events/number of samples should be greater than 10%, we further excluded renal chromophobe and renal papillary carcinoma from the main analysis as presented in **Fig. 4h**.

We observed the direction of hazard ratios (HRs) of the four types of binarized TmS and the continuous $\log_2$(TmS) are consistent, although the statistical significance of median, lower tertile and upper tertile binarized TmS and $\log_2$(TmS) decreased as expected. We also observed, as expected, that when the cutoff values deviate from the rpart-based cutoff to become the tertiles or median, the corresponding effect size of the hazard ratios reduces in accordance with the degree of deviation. Overall, depending

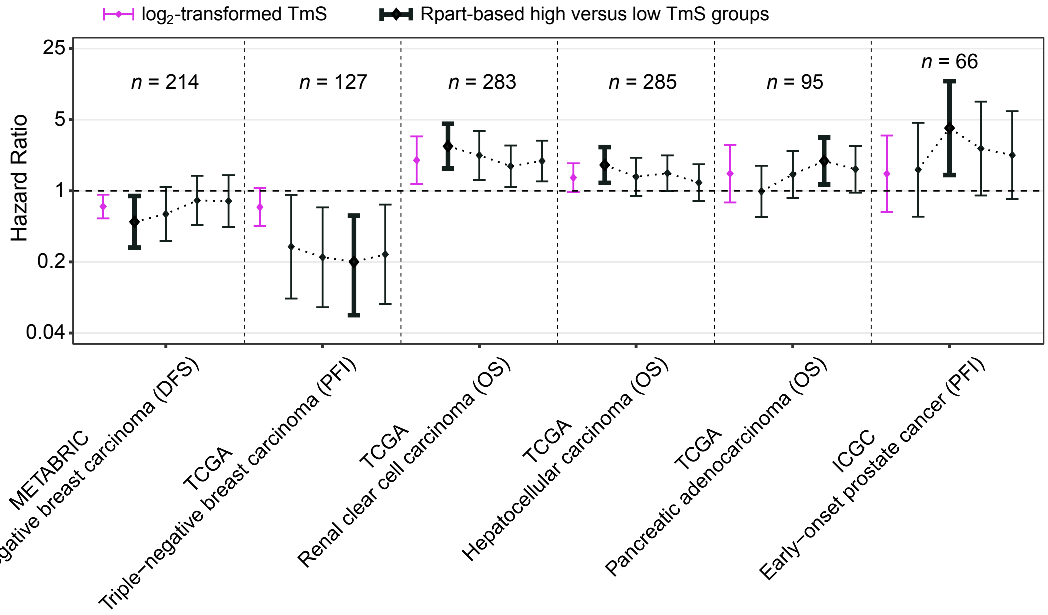

Supplementary Note Figure 25. Forest plots of hazard ratios (center points) and 95% of CIs (error bars) of four types of binarized TmS and the continuous $\log_2$(TmS) across six datasets from five cancer types. The HRs in pink are for $\log_2$(TmS). HRs using four different cutoffs are ordered from the lowest to the highest cutoff values: lower tertile, median, upper tertile and rpart. The rpart result is shown in bold, whose location with regard to the other three cutoffs vary across cancer types). For each cancer type, the number of samples is indicated on the top.

on the characteristics of the patient population of interest, tertile or median cutoffs may also be used to study the prognostication effect of TmS.

Using the TCGA datasets from 12 cancer types, we fitted multivariate CoxPH models with Age, TmS, and Stage as the baseline model, and the interaction of TmS and Stage, a cell cycle score[68], expression levels of *POU5F1, KLF4, SOX2, MYC*[69], as candidate predictors of OS or PFI. To select the optimal predictors of the Cox model, we implemented a stepwise model selection method with a forward-backward search for the Cox model based on the Bayesian Information Criterion (BIC). Across all cancer types, the cell cycle score and *MYC* expression were never selected. Consistent with previous reports, *KLF4* was selected in lung adenocarcinoma[70], *POU5F1* was selected in bladder urothelial carcinoma[71], and *SOX2* was selected in renal clear cell carcinoma[72] and triple negative breast carcinoma. The main effect of TmS in prognostication remains statistically significant after these features are included.

We further tested the hypothesis whether the varying sample sizes indeed biased the clinical outcomes associated with TmS. Across the 15 cancer types, we set a consistent sample size around its median value, 300, bootstrapped 300 samples within each cancer type, and evaluated the prognostication effect of TmS in these 4,500 pan-cancer samples. This procedure was repeated 1,000 times. **Supplementary Note Fig. 26** shows the pan-cancer results from one bootstrapped set, supporting our finding that high TmS is associated with worse prognosis across cancers. Across all iterations, we obtain the hazard ratios (HRs) of TmS and the corresponding 95% Confidence Intervals (CI), and found all of them to be significantly different from 1. For OS, we find the following HR for TmS across all samples: median 1.67,

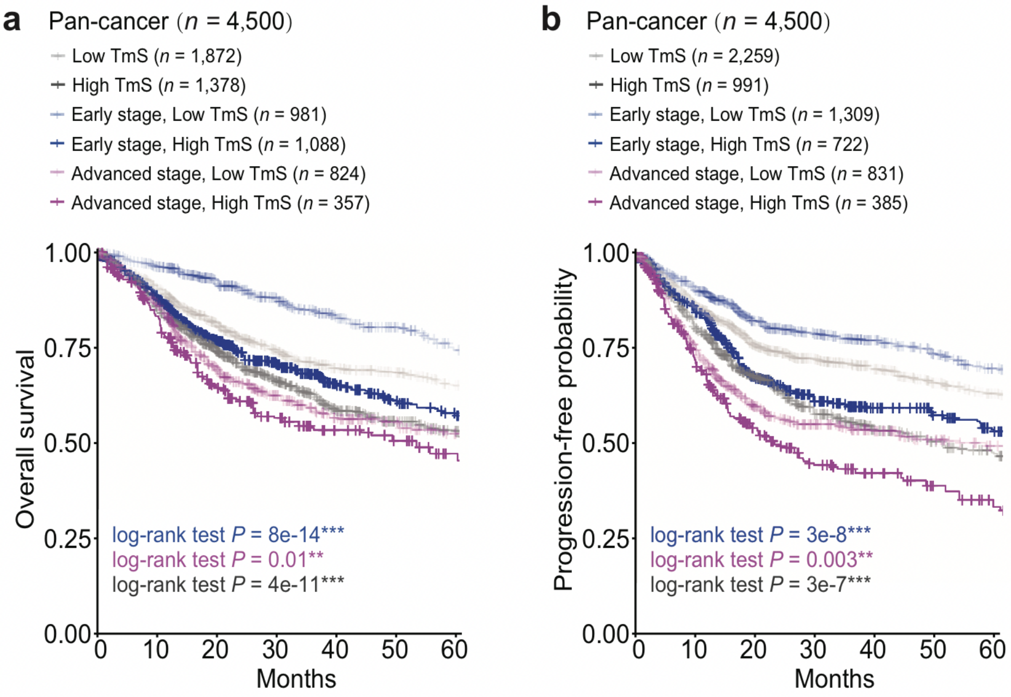

Supplementary Note Figure 26. Kaplan-Meier curves of overall survival (**a**) and progression-free probability (**b**) for one bootstrap sample from 15 cancer types in TCGA.

95%CI [1.38, 1.87]; across early stage samples: median 2.34, 95%CI [2.02, 2.73]; across advanced stage samples: median 1.18, 95%CI [1.00, 1.40]. For the PFI, we find the following HR for TmS across all samples: median 1.64, 95%CI [1.34, 1.83]; across early stage samples: median 2.16, 95%CI [1.84, 2.49]); across advanced stage samples: median 1.25, 95%CI [1.06, 1.46]. We conclude that the pan-cancer analysis with balanced sample sizes gives the same results as the original analysis.

3.2.2. Identification of patients treated without systemic therapy in TCGA

We identified a cohort of patients where chemotherapy and/or radiotherapy are generally not indicated using NCCN guidelines as well as expert opinion for each cancer type (https://www.nccn.org/guidelines/category_1) (**Supplementary Table 6**) in TCGA. We performed Cox regression analysis in six cancer types where the number of events / number of samples > 10%: lung adenocarcinoma, lung squamous cell carcinoma, renal papillary carcinoma, renal clear cell carcinoma, renal chromophobe, and hepatocellular carcinoma. Patients of cancer types except hepatocellular carcinoma subgroup are mainly with early-stage, we therefore used PFI for these cancer types.

3.3. Regional TmS analysis in TRACERx

We calculated the percentage of copy number alteration burden per region, the percentage of subclonal copy number alteration (CNA) per region, and the percentage of subclonal copy number alteration per patient. For each chromosomal segment *i* in tumor region *k*, we use an indicator function I_{ik} to represent the copy number alteration (gain and loss) event[43]:

$$I_{ik}=\begin{cases} 1 & \text{if } \alpha>\log_2(2.5/2) \text{ or } \alpha<\log_2(1.5/2) \\ 0 & \text{otherwise} \end{cases},$$

where $\alpha=\frac{cnTotal_{ik}}{Ploidy_k}$ and $cnTotal_{ik}$ is the integer total copy number of this segment[43].

We then define the percentage of CNA burden for each region as the percentage of genome affected by copy number alterations,

$$\text{percentage of CNA burden}_k=\frac{\sum_{i=1}^{nS} D_i \times I_{ik}}{\sum_{i=1}^{nS} D_i} \times 100\%,$$

where nS and D_i denotes the number of shared segments, *i.e.,* segments of the genome where copy number status is available across all regions, and the length of shared segment *i* across regions, respectively.

Further, for each region, whether the segment *i* has a subclonal CNA event is defined as

$$S_{ik}=\begin{cases} 1 & I_{ik}=1 \text{ and } \sum_{k=1}^{K} I_{ik} \neq K \\ 0 & \text{Otherwise} \end{cases},$$

where K is the total number of regions for a given tumor sample.

Further, we define T_i as an indicator function which denotes whether there is an CNA event (including clonal and subclonal) on shared segment i.

$$T_i=\begin{cases} 1 & 0 < \sum_{k=1}^{K} I_{ik} \leq K \\ 0 & Otherwise \end{cases}.$$

Therefore, the percentage of subclonal CNA for region k (percentage of subclonal CNA per region) is defined as

$$percentage\ of\ subclonal\ CNA_k=\frac{\sum_{i=1}^{nS} D_i \times S_{ik}}{\sum_{i=1}^{nS} D_i \times T_i} \times 100\%.$$

We then introduce S_i as an indicator function representing the union of subclonal CNA events on shared segment i across regions: $S_i = \bigcup_{k=1}^{K} S_{ik}$. Correspondingly, the percentage of subclonal CNA for each patient is defined as

$$percentage\ of\ subclonal\ CNA=\frac{\sum_{i=1}^{nS} D_i \times S_i}{\sum_{i=1}^{nS} D_i \times T_i} \times 100\%.$$

Across regions, the Spearman correlation coefficient between log_2(TmS) and percentage of subclonal CNA per region is 0.44; the Spearman correlation coefficient between log_2(TmS) and copy number aberration burden per region is 0.26. The difference between these two correlation coefficients between is statistically significant (bootstrapping 1,000 times, mean difference = 0.2, 95% confidence interval: [0.04, 0.37]).

Two subclonal structures in two regions can be linearly related to each other, or have a common ancestor, but develop a branching relationship, which is more common in this dataset. For example, a linear relationship can be described as a parent and child relationship, where two subclonal structures share overlapped segments and one structure evolves further than the other. For a branching relationship, two subclonal structures usually share a common node (ancestor), and two structures evolve in different directions. The subclonal structures of 5 out of the 30 patients are defined as linear relationships; others are defined as branching relationships (**Supplementary Note Table 8**). For each evolutionary relationship per patient sample, we defined the range of TmS = log_2(maximum TmS)-log_2(minimum TmS) across regions. We observed a strong correlation between *log2*(TmSmax) and percentage of subclonal CNA among 30 patients with multi-region sequencing data (Spearman correlation coefficient r = 0.69). To further explore the underlying relationship between *log2*(TmSmax) and all variables (*e.g.*, percentage of subclonal CNA, number of regions, range of TmS, evolutionary relationship and their interactions) across patients, we fit linear regression models by taking TmSmax as the response variable and others as predictors. The best model was selected by stepwise adding or dropping one predictor that achieves the best BIC (Bayesian Information Criteria) (**Supplementary Note**

Table 9a). We also adopted a logistic regression model by taking the evolutionary relationship as the response variable, and after the model selection (likelihood ratio test), percentage of subclonal CNA and range of TmS were chosen separately as predictor variables (**Supplementary Note Table 9b-c, Supplementary Note Figure 27**).

Supplementary Note Table 8. Evolutionary relationships for 30 TRACERx patients with multi-region samples.

Patient	Histology	Evolutionary Relationships	Region with Maximum TmS	Maximum TmS	Region with Minimum TmS	Minimum TmS	Range of TmS
CRUK0005	LUAD	Branching	R4	3.5	R3	3.4	0.050
CRUK0013	LUAD	Branching	R2	3.0	R3	1.6	0.90
CRUK0017	LUAD	Branching	R4	1.9	R1	1.3	0.58
CRUK0018	LUAD	Branching	R4	3.4	R2	0.88	2.0
CRUK0021	LUAD	Branching	R1	1.8	R2	1.7	0.050
CRUK0023	LUAD	Branching	R4	2.7	R1	0.80	1.7
CRUK0024	LUAD	Branching	R1	4.1	R4	2.2	0.89
CRUK0025	LUAD	Branching	R3	1.8	R1	0.87	1.0
CRUK0029	LUAD	Branching	R2	4.0	R6	2.2	0.85
CRUK0030	LUAD	Linear	R2	2.7	R3	2.4	0.14
CRUK0033	LUAD	Linear	R1	1.3	R2	0.85	0.58
CRUK0036	LUAD	Branching	R4	7.4	R2	5.4	0.47
CRUK0037	LUAD	Branching	R2	7.5	R3	1.5	2.3
CRUK0039	LUAD	Branching	R1	2.3	R2	2.0	0.19
CRUK0041	LUAD	Branching	R4	2.5	R1	1.8	0.48
CRUK0046	LUAD	Branching	R2	2.5	R1	1.6	0.65
CRUK0047	LUAD	Branching	R2	2.7	R1	2.4	0.16
CRUK0050	LUAD	Linear	R4	1.1	R3	0.98	0.19
CRUK0057	LUAD	Branching	R1	2.7	R2	2.0	0.40
CRUK0062	LUSC	Branching	R7	4.0	R2	1.7	1.2
CRUK0065	LUSC	Branching	R3	3.9	R1	1.7	1.2
CRUK0067	LUSC	Branching	R1	2.2	R3	1.3	0.73
CRUK0069	LUSC	Branching	R1	3.5	R3	0.81	2.1
CRUK0070	LUSC	Branching	R6	1.4	R1	0.85	0.72
CRUK0076	LUSC	Linear	R2	2.9	R4	2.6	0.16
CRUK0077	LUSC	Branching	R1	3.7	R2	1.4	1.5
CRUK0079	LUSC	Branching	R1	3.5	R3	2.0	0.81
CRUK0083	LUSC	Branching	R3	3.7	R1	1.2	1.6
CRUK0084	LUSC	Branching	R2	0.91	R3	0.72	0.34
CRUK0090	LUSC	Linear	R1	1.3	R2	1.0	0.30

Supplementary Note Table 9. Summary of regression models

a. linear regression model with maximum TmS as response variable. *P* values of two-sided t tests are indicated by asterisks (* $P < 0.05$, ** < 0.01, *** < 0.001).

Variable	Coefficient	T-statistics	Standard Error	*P* value
Intercept	0.3	0.7	0.5	0.5
% Subclonal CNA	2.9	4.7	0.6	$8x10^{-05}$ ***
Range of TmS	0.3	0.4	0.6	0.7
No. of Regions	-0.2	-1	0.1	0.2
% Subclonal CNA * Range of TmS	-1.8	-2.5	0.7	0.02*
Range of TmS * No. of Region	0.3	2.4	0.1	0.03*
F-statistics	R-squared	Adjusted R-squared	RMSE	*P* value
10.2 on 5 and 24 DF	0.7	0.6	0.4	$3x10^{-05}$ ***

b. Logistics regression model with Range of TmS as predictor and Evolutionary Relationships (Branching = 1, Linear = 0) as response variable. *P* values of two-sided z tests are indicated by asterisks (* $P < 0.05$, ** < 0.01, *** < 0.001).

Variables	Coefficient	Z-statistics	Standard Error	*P* value
Range of TmS	3.3	2.7	1.3	0.008**

c. Logistics regression model with % Subclonal CNA as predictor and Evolutionary Relationships (Branching = 1, Linear = 0) as response variable. *P* values of two-sided z tests are indicated by asterisks (* $P < 0.05$, ** < 0.01, *** < 0.001).

Variables	Coefficient	Z-statistics	Standard Error	*P* value
% Subclonal CNA	4.3	3.1	1.4	0.002**

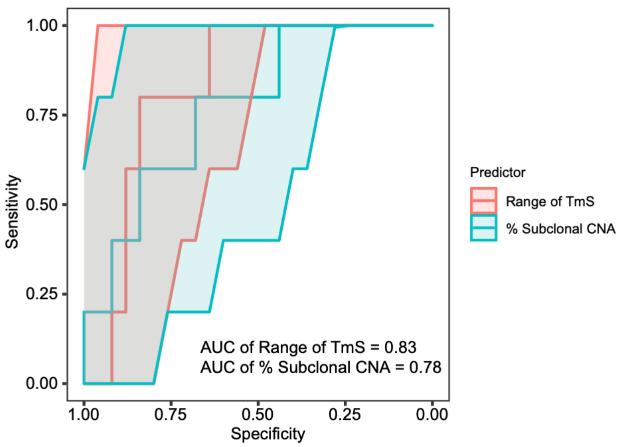

Supplementary Note Figure 27. ROC curves for predicting evolutionary relationships: branching versus linear. Two logistic models were used (**Supplementary Note Table 9b-c**), with either the range of TmS or the percentage of subclonal CNA as the predictor. The 95% confidence intervals and area under the ROC curves (AUC) are provided.

References

1. Ma, L. *et al.* Tumor Cell Biodiversity Drives Microenvironmental Reprogramming in Liver Cancer. *Cancer Cell* **36**, 418–430 (2019).

2. Lambrechts, D. *et al.* Phenotype molding of stromal cells in the lung tumor microenvironment. *Nat. Med.* **24**, 1277–1289 (2018).

3. Lee, J. J. *et al.* Elucidation of Tumor-Stromal Heterogeneity and the Ligand-Receptor Interactome by Single-Cell Transcriptomics in Real-world Pancreatic Cancer Biopsies. *Clin. Cancer Res.* **27**, 5912–5921 (2021).

4. Satija, R., Farrell, J. A., Gennert, D., Schier, A. F. & Regev, A. Spatial reconstruction of single-cell gene expression data. *Nat. Biotechnol.* **33**, 495–502 (2015).

5. Li, H. *et al.* Reference component analysis of single-cell transcriptomes elucidates cellular heterogeneity in human colorectal tumors. *Nat. Genet.* **49**, 708–718 (2017).

6. Puram, S. V. *et al.* Single-Cell Transcriptomic Analysis of Primary and Metastatic Tumor Ecosystems in Head and Neck Cancer. *Cell* **171**, 1611-1624.e24 (2017).

7. Peng, J. *et al.* Single-cell RNA-seq highlights intra-tumoral heterogeneity and malignant progression in pancreatic ductal adenocarcinoma. *Cell Res.* **29**, 725–738 (2019).

8. Hashimoto, K. *et al.* Single-cell transcriptomics reveals expansion of cytotoxic CD4 T cells in supercentenarians. *Proc. Natl. Acad. Sci. U. S. A.* **116**, 24242–24251 (2019).

9. Wang, Y. J. *et al.* Comparative analysis of commercially available single-cell RNA sequencing platforms for their performance in complex human tissues. Preprint at https://www.biorxiv.org/content/10.1101/541433v1 (2019).

10. Zack, T. I. *et al.* Pan-cancer patterns of somatic copy number alteration. *Nat. Genet.* **45**, 1134–1140 (2013).

11. Kim, C. *et al.* Chemoresistance Evolution in Triple-Negative Breast Cancer Delineated by Single-Cell Sequencing. *Cell* **173**, 879–893 (2018).

12. Benjamini, Y. & Hochberg, Y. Controlling for the False Discovery Rate: a Practical and Powerful Approach to Multiple Testing. *J. R. Stat. Soc. Ser. B* **57**, 289–300 (1995).

13. Ruan, H. *et al.* Single-cell transcriptome analysis of diffuse large B cells in cerebrospinal fluid of central nervous system lymphoma. *iScience* **24**, 102972 (2021).

14. Van Loo, P. *et al.* Allele-specific copy number analysis of tumors. *Proc. Natl. Acad. Sci. U. S. A.* **107**, 16910–16915 (2010).

15. Carter, S. L. *et al.* Absolute quantification of somatic DNA alterations in human cancer. *Nat. Biotechnol.* **30**, 413–421 (2012).

16. Lovén, J. *et al.* Revisiting global gene expression analysis. *Cell* **151**, 476–482 (2012).

17. Newman, A. M. *et al.* Robust enumeration of cell subsets from tissue expression profiles. *Nat. Methods* **12**, 453–457 (2015).

18. Aran, D., Hu, Z. & Butte, A. J. xCell: Digitally portraying the tissue cellular heterogeneity landscape. *Genome Biol.* **18**, 1–14 (2017).

19. Li, B. *et al.* Comprehensive analyses of tumor immunity: Implications for cancer immunotherapy. *Genome Biol.* **17**, 1–16 (2016).

20. Wang, Z. *et al.* Transcriptome Deconvolution of Heterogeneous Tumor Samples with Immune Infiltration. *iScience* **9**, 451–460 (2018).

21. Van Loo, P. *et al.* Allele-specific copy number analysis of tumors. *Proc. Natl. Acad. Sci. U. S. A.* **107**, 16910–16915 (2010).

22. Quon, G. *et al.* Computational purification of individual tumor gene expression profiles leads to significant improvements in prognostic prediction. *Genome Med.* **5**, 29 (2013).

23. Lehmann, E. L. & Casella, G. Theory of Point Estimation, Second Edition. (Springer, 1998).

24. Raue, A. *et al.* Structural and practical identifiability analysis of partially observed dynamical models by exploiting the profile likelihood. *Bioinformatics* **25**, 1923–1929 (2009).

25. Besag, J. On the Statistical Analysis of Dirty Pictures. *J. R. Stat. Soc. Ser. B* **48**, 259–279 (1986).

26. Cox, D. R. & Reid, N. A Note on the Calculation of Adjusted Profile Likelihood. *J. R. Stat. Soc. Ser. B* **55**, 467–471 (1993).

27. Venzon, D. J. & Moolgavkar, S. H. A Method for Computing Profile-Likelihood-Based Confidence Intervals. *Appl. Stat.* **37**, 87–94 (1988).

28. Hartigan, J. A. & Hartigan, P. M. The Dip Test of Unimodality. *Ann. Stat.* **13**, 70–84 (1985).

29. Grossman, Robert L., Heath, Allison P., Ferretti, Vincent, Varmus, Harold E., Lowy, Douglas R., Kibbe, Warren A., Staudt, L. M. Toward a Shared Vision for Cancer Genomic Data. *N. Engl. J. Med.* **375**, 1109–1112 (2016).

30. Aran, D., Sirota, M. & Butte, A. J. Systematic pan-cancer analysis of tumour purity. *Nat. Commun.* **6**, 1–12 (2015).

31. Alexandrov, L. B. *et al.* Mutational signatures associated with tobacco smoking in human cancer.

Science **354**, 618–622 (2016).

32. Tamborero, D. *et al.* Cancer Genome Interpreter annotates the biological and clinical relevance of tumor alterations. *Genome Med.* **10**, 25 (2018).

33. Corces, M. R. *et al.* The chromatin accessibility landscape of primary human cancers. *Science* **362**, eaav1898 (2018).

34. Yu, G., Wang, L. G. & He, Q. Y. ChIPseeker: an R/Bioconductor package for ChIP peak annotation, comparison and visualization. *Bioinformatics* **31**, 2382–2383 (2015).

35. Koboldt, D. C. *et al.* Comprehensive molecular portraits of human breast tumours. *Nature* **490**, 61–70 (2012).

36. Mermel, C. H. *et al.* GISTIC2.0 facilitates sensitive and confident localization of the targets of focal somatic copy-number alteration in human cancers. *Genome Biol.* **12**, R41 (2011).

37. Cerami, E. *et al.* The cBio Cancer Genomics Portal: An open platform for exploring multidimensional cancer genomics data. *Cancer Discov.* **10**, 401–404 (2012).

38. Abeshouse, A. *et al.* The Molecular Taxonomy of Primary Prostate Cancer. *Cell* **163**, 1011–1025 (2015).

39. Lawrence, M. S. *et al.* Comprehensive genomic characterization of head and neck squamous cell carcinomas. *Nature* **517**, 576–582 (2015).

40. Linehan, W. M. *et al.* Comprehensive Molecular Characterization of Papillary Renal-Cell Carcinoma. *N. Engl. J. Med.* **374**, 135–145 (2016).

41. Gerhauser, C. *et al.* Molecular Evolution of Early-Onset Prostate Cancer Identifies Molecular Risk Markers and Clinical Trajectories. *Cancer Cell* **34**, 996–1011 (2018).

42. Weischenfeldt, J. *et al.* Integrative Genomic Analyses Reveal an Androgen-Driven Somatic Alteration Landscape in Early-Onset Prostate Cancer. *Cancer Cell* **23**, 159–170 (2013).

43. Li, H. Aligning sequence reads, clone sequences and assembly contigs with BWA-MEM. Preprint at https://arxiv.org/abs/1303.3997 (2013).

44. Curtis, C. *et al.* The genomic and transcriptomic architecture of 2,000 breast tumours reveals novel subgroups. *Nature* **486**, 346–352 (2012).

45. Jamal-Hanjani, M. *et al.* Tracking the evolution of non-small-cell lung cancer. *N. Engl. J. Med.* **376**, 2109–2121 (2017).

46. Rosenthal, R. *et al.* Neoantigen-directed immune escape in lung cancer evolution. *Nature* **567**,

479–485 (2019).

47. Biswas, D. *et al.* A clonal expression biomarker associates with lung cancer mortality. *Nat. Med.* **25**, 1540–1548 (2019).

48. Dobin, A. *et al.* STAR: Ultrafast universal RNA-seq aligner. *Bioinformatics* **29**, 15–21 (2013).

49. Li, B. & Dewey, C. N. RSEM: Accurate transcript quantification from RNA-Seq data with or without a reference genome. *BMC Bioinformatics* **12**, 323 (2011).

50. Wu, H., Wang, C. & Wu, Z. A new shrinkage estimator for dispersion improves differential expression detection in RNA-seq data. *Biostatistics* **14**, 232–243 (2013).

51. Eisenberg, E. & Levanon, E. Y. Human housekeeping genes, revisited. *Trends Genet.* **29**, 569–574 (2013).

52. Dempster, J. M. *et al.* Agreement between two large pan-cancer CRISPR-Cas9 gene dependency data sets. *Nat. Commun.* **10**, 1–14 (2019).

53. Liberzon, A. *et al.* The Molecular Signatures Database Hallmark Gene Set Collection. *Cell Syst.* **1**, 417–425 (2015).

54. Subramanian, A. *et al.* Gene set enrichment analysis: A knowledge-based approach for interpreting genome-wide expression profiles. *Proc. Natl. Acad. Sci. U. S. A.* **102**, 15545–15550 (2005).

55. Reimand, J. *et al.* g:Profiler-a web server for functional interpretation of gene lists (2016 update). *Nucleic Acids Res.* **44**, W83–W89 (2016).

56. Pang, B. *et al.* Prognostic role of PIK3CA mutations and their association with hormone receptor expression in breast cancer: A meta-analysis. *Sci. Rep.* **4**, 6255 (2014).

57. Agrawal, N. *et al.* Integrated Genomic Characterization of Papillary Thyroid Carcinoma. *Cell* **159**, 676–690 (2014).

58. Uno, H., Cai, T., Tian, L. & Wei, L. J. Evaluating prediction rules for t-year survivors with censored regression models. *J. Am. Stat. Assoc.* **102**, 527–537 (2007).

59. Gerstung, M. *et al.* The evolutionary history of 2,658 cancers. *Nature* **578**, 122–128 (2020).

60. Takahashi, K. & Yamanaka, S. Induction of pluripotent stem cells from mouse embryonic and adult fibroblast cultures by defined factors. *Cell* **126**, 663–676 (2006).

61. Favero, F. *et al.* Sequenza: Allele-specific copy number and mutation profiles from tumor sequencing data. *Ann. Oncol.* **26**, 64–70 (2015).

62. Wang, K. *et al.* PennCNV: An integrated hidden Markov model designed for high-resolution copy number variation detection in whole-genome SNP genotyping data. *Genome Res.* **17**, 1665–1674 (2007).

63. Ritchie, M. E. *et al.* Limma powers differential expression analyses for RNA-sequencing and microarray studies. *Nucleic Acids Res.* **43**, e47 (2015).

64. Ardlie, K. G. *et al.* The Genotype-Tissue Expression (GTEx) pilot analysis: Multitissue gene regulation in humans. *Science* **348**, 648–660 (2015).

65. Johnson, W. E., Li, C. & Rabinovic, A. Adjusting batch effects in microarray expression data using empirical Bayes methods. *Biostatistics* **8**, 118–127 (2007).

66. Liu, J. *et al.* An Integrated TCGA Pan-Cancer Clinical Data Resource to Drive High-Quality Survival Outcome Analytics. *Cell* **173**, 400–416 (2018).

67. Therneau, T. M. & Atkinson, E. J. *An Introduction to Recursive Partitioning Using the RPART Routines*. Technical report no. 61 (Mayo Clinic, section of statistics, Minnesota, 1997).

68. Lundberg, A. *et al.* A pan-cancer analysis of the frequency of DNA alterations across cell cycle activity levels. *Oncogene* **39**, 5430–5440 (2020).

69. Lorenzin, F. *et al.* Different promoter affinities account for specificity in MYC-dependent gene regulation. *Elife* **5**, e15161 (2016).

70. Arora, S. *et al.* Comprehensive integrative analysis reveals the association of klf4 with macrophage infiltration and polarization in lung cancer microenvironment. *Cells* **10**, 2091 (2021).

71. Moad, M. *et al.* A novel model of urinary tract differentiation, tissue regeneration, and disease: Reprogramming human prostate and bladder cells into induced pluripotent stem cells. *Eur. Urol.* **64**, 753–761 (2013).

72. Grimm, D. *et al.* The role of SOX family members in solid tumours and metastasis. *Semin. Cancer Biol.* **67**, 122–153 (2020).
