# Supplementary material for: Estimation of tumor cell total mRNA expression in 15 cancer types predicts disease progression
Source: Nat Biotechnol. 2022 Jun 13;40(11):1624–33. doi: 10.1038/s41587-022-01342-x (PMC9646498; doi:10.1038/s41587-022-01342-x)
Supplement: Supplementary file 2 — Reporting Summary [file 41587_2022_1342_MOESM2_ESM.pdf]

nature research

Corresponding author(s): Wenyi Wang

Last updated by author(s): Apr 26, 2022

Reporting Summary

Nature Research wishes to improve the reproducibility of the work that we publish. This form provides structure for consistency and transparency in reporting. For further information on Nature Research policies, see our Editorial Policies and the Editorial Policy Checklist.

Statistics

For all statistical analyses, confirm that the following items are present in the figure legend, table legend, main text, or Methods section.

n/a	Confirmed	
☐	☒	The exact sample size (*n*) for each experimental group/condition, given as a discrete number and unit of measurement
☐	☒	A statement on whether measurements were taken from distinct samples or whether the same sample was measured repeatedly
☐	☒	The statistical test(s) used AND whether they are one- or two-sided<br>*Only common tests should be described solely by name; describe more complex techniques in the Methods section.*
☐	☒	A description of all covariates tested
☐	☒	A description of any assumptions or corrections, such as tests of normality and adjustment for multiple comparisons
☐	☒	A full description of the statistical parameters including central tendency (e.g. means) or other basic estimates (e.g. regression coefficient) AND variation (e.g. standard deviation) or associated estimates of uncertainty (e.g. confidence intervals)
☐	☒	For null hypothesis testing, the test statistic (e.g. *F*, *t*, *r*) with confidence intervals, effect sizes, degrees of freedom and *P* value noted<br>*Give P values as exact values whenever suitable.*
☒	☐	For Bayesian analysis, information on the choice of priors and Markov chain Monte Carlo settings
☐	☒	For hierarchical and complex designs, identification of the appropriate level for tests and full reporting of outcomes
☐	☒	Estimates of effect sizes (e.g. Cohen's *d*, Pearson's *r*), indicating how they were calculated

Our web collection on statistics for biologists contains articles on many of the points above.

Software and code

Policy information about availability of computer code

Data collection	No commercial, open source, or custom software or code was used for data collection.
Data analysis	Individual software components are as follows:<br>R(v 4.1.2), DeMixT (v 1.2.2), ASCAT (v2.5.2), sequenza (v 3.0.0), Seurat (v 3.0), Monocle 2 (v 2.14.0), CytoTRACE (v 0.3.3)<br>Downstream analyses and statistical analyses were performed using package as follows:<br>rpart (v 4.1.15), survival (v 3.2.0), survminer (v 0.4.9), gprofiler2 (v 0.2.0), fgsea (v 1.14.0), DSS (v 2.42.0), ChIPseeker (v 3.11), ComBat (v 3.20.0), limma (v 3.50.1), GeneOverlap (v 1.24.0), ComplexHeatmap (v 2.4.3), RColorBrewer (v1.1.2)

For manuscripts utilizing custom algorithms or software that are central to the research but not yet described in published literature, software must be made available to editors and reviewers. We strongly encourage code deposition in a community repository (e.g. GitHub). See the Nature Research guidelines for submitting code & software for further information.

Data

Policy information about availability of data

All manuscripts must include a data availability statement. This statement should provide the following information, where applicable:

- Accession codes, unique identifiers, or web links for publicly available datasets
- A list of figures that have associated raw data
- A description of any restrictions on data availability

The UMI counts of the hepatocellular carcinoma single cell RNA sequencing data were downloaded from the Gene Expression Omnibus (GEO) with the accession code GSE125449. The UMI counts and cell type annotations of the lung adenocarcinoma single cell RNA sequencing data were downloaded from the ArrayExpress under accessions E-MTAB-6149. The UMI counts of the colorectal adenocarcinoma single cell RNA sequencing data are available at http://crcmoonshot.org/?page_id=189. FASTQ files of single-cell RNA sequencing data from pancreatic cancer will be publicly available on the GEO with the

accession code GSE156405.
Raw read counts from the mixed cell-line study were downloaded from GEO with accession code GSE121127.
Raw read counts of RNA sequencing data, clinical data, and somatic mutations from 7,054 tumor samples across 15 TCGA cancer types are available for download from the Genomic Data Commons Data Portal (https://portal.gdc.cancer.gov/). ATACseq data for TCGA samples were downloaded from https://science.sciencemag.org/content/362/6413/eaav1898/tab-figures-data.
Clinical information of ICGC-EOPC was downloaded from https://www.sciencedirect.com/science/article/pii/S1535610818304823?via%3Dihub#gs1.
All primary METABRIC data including Affymetrix SNP 6.0 CEL files and Illumina HT 12 gene expression arrays, are available at the EGA (EGAS00000000083), and may be downloaded from https://ega-archive.org/studies/EGAS00000000083. Clinical information of METABRIC was downloaded from https://www.cbioportal.org/study/clinicalData?id=brca_metabric.
Clinical information of TRACERx was downloaded from https://www.nejm.org/doi/full/10.1056/NEJMoa1616288#article_supplementary_material.
WES data of TRACERx was downloaded from https://ega-archive.org/studies/EGAS00001002247.
RNAseq data of TRACERx was downloaded from https://ega-archive.org/studies/EGAS00001003458.
TmS values of all samples and the identified intrinsic tumor signature genes for this study are available for download at https://github.com/wwylab/TmS.

Field-specific reporting

Please select the one below that is the best fit for your research. If you are not sure, read the appropriate sections before making your selection.

☒ Life sciences ☐ Behavioural & social sciences ☐ Ecological, evolutionary & environmental sciences

For a reference copy of the document with all sections, see nature.com/documents/nr-reporting-summary-flat.pdf

Life sciences study design

All studies must disclose on these points even when the disclosure is negative.

Sample size	No statistical methods were used to predetermine sample size. Sample size was determined by the availability of the data.
Data exclusions	Samples with missing RNAseq data or DNA sequencing data were excluded from the analysis.
Replication	All attempts at replication were successful.
Randomization	There are no experimental groups in this study.
Blinding	There are no experimental groups in this study.

Reporting for specific materials, systems and methods

We require information from authors about some types of materials, experimental systems and methods used in many studies. Here, indicate whether each material, system or method listed is relevant to your study. If you are not sure if a list item applies to your research, read the appropriate section before selecting a response.

Materials & experimental systems

n/a	Involved in the study	
☒	☐	Antibodies
☒	☐	Eukaryotic cell lines
☒	☐	Palaeontology and archaeology
☒	☐	Animals and other organisms
☐	☒	Human research participants
☒	☐	Clinical data
☒	☐	Dual use research of concern

Methods

n/a	Involved in the study	
☒	☐	ChIP-seq
☒	☐	Flow cytometry
☒	☐	MRI-based neuroimaging

Human research participants

Policy information about studies involving human research participants

Population characteristics	Demographically, the single-cell RNA sequencing datasets from three colorectal patients includes 1 male at age 45, 1 male at 37 and 1 female at age 63.
Recruitment	The three colorectal adenocarcinoma patients with single-cell RNA sequencing data were identified prior to surgery or biopsy and were asked to prospectively sign consent for participation on the IRB approved protocol LAB10-0982 after discussion of risks and benefits. Prospective consenting of patients was required due to the intent to utilize fresh tumor tissue. No known selection bias other than the fact that these patients went through surgery at MD Anderson.

Ethics oversight

The three colorectal adenocarcinoma patient samples were obtained with informed consent and were approved by the Human Subjects Protection Office, Clinical Research Committee as well as five separate Institutional Review Boards (IRB) at the MD Anderson Cancer Center, in accordance with the Declaration of Helsinki.

Note that full information on the approval of the study protocol must also be provided in the manuscript.
