# Supplementary material for: Estimation of tumor cell total mRNA expression in 15 cancer types predicts disease progression
Source: Nat Biotechnol. 2022 Jun 13;40(11):1624–33. doi: 10.1038/s41587-022-01342-x (PMC9646498; doi:10.1038/s41587-022-01342-x)
Supplement: Supplementary file 8 — Summary of patients without systemic therapy across cancers [file 41587_2022_1342_MOESM8_ESM.docx]

**Supplementary Table 6. Summary of patients without systemic therapy across cancers.**

| Cancer type | Untreated (surgery only) | Sample size | Outcome type | Number of events |
| --- | --- | --- | --- | --- |
| Head & neck squamous cell carcinoma (HPV-) | T1N0 or T2N0 and oral cavity | 26 | PFI | 4 |
| Lung adenocarcinoma | Stage I | 205 | PFI | 65 |
| Lung squamous cell carcinoma | Stage I | 194 | PFI | 41 |
| Colorectal carcinoma | Stage I | 85 | PFI | 4 |
| Renal papillary carcinoma | Stage I,II,III | 133 | PFI | 24 |
| Renal clear cell carcinoma | Stage I,II,III | 245 | PFI | 44 |
| Renal chromophobe | Stage I,II,III | 52 | PFI | 7 |
| Hepatocellular carcinoma | All samples | 305 | OS | 98 |
| Prostate adenocarcinoma | GS=7 & pT<=T2c | 72 | PFI | 5 |
| Early-onset prostate cancer (ICGC-EOPC) | GS=7 & pT<=T2c | 41 | PFI | 3 |
